# Excess mortality in Ukraine during the course of COVID-19 pandemic in 2020–2021

**DOI:** 10.1038/s41598-023-33113-2

**Published:** 2023-04-27

**Authors:** Aleksandr Shishkin, Pema Lhewa, Chen Yang, Yuriy Gankin, Gerardo Chowell, Michael Norris, Pavel Skums, Alexander Kirpich

**Affiliations:** 1 Department of Population Health Sciences, School of Public Health, Georgia State University, Atlanta, GA USA; 2 Department of Biology, Georgia State University, Atlanta, GA USA; 3 Quantori, Cambridge, MA USA; 4 Department of Geography, University of Florida, Gainesville, FL USA; 5 Emerging Pathogens Institute, University of Florida, Gainesville, FL USA; 6 Department of Computer Science, Georgia State University, Atlanta, GA USA

**Keywords:** Infectious diseases, Health care, Disease prevention, Health care economics, Health policy, Public health

## Abstract

In this work, the COVID-19 pandemic burden in Ukraine is investigated retrospectively using the excess mortality measures during 2020–2021. In particular, the epidemic impact on the Ukrainian population is studied via the standardized both all-cause and cause-specific mortality scores before and during the epidemic. The excess mortality counts during the pandemic were predicted based on historic data using parametric and nonparametric modeling and then compared with the actual reported counts to quantify the excess. The corresponding standardized mortality *P*-score metrics were also compared with the neighboring countries. In summary, there were three “waves” of excess all-cause mortality in Ukraine in December 2020, April 2021 and November 2021 with excess of 32%, 43% and 83% above the expected mortality. Each new “wave” of the all-cause mortality was higher than the previous one and the mortality “peaks” corresponded in time to three “waves” of lab-confirmed COVID-19 mortality. The lab-confirmed COVID-19 mortality constituted 9% to 24% of the all-cause mortality during those three peak months. Overall, the mortality trends in Ukraine over time were similar to neighboring countries where vaccination coverage was similar to that in Ukraine. For cause-specific mortality, the excess observed was due to pneumonia as well as circulatory system disease categories that peaked at the same times as the all-cause and lab-confirmed COVID-19 mortality, which was expected. The pneumonias as well as circulatory system disease categories constituted the majority of all cases during those peak times. The seasonality in mortality due to the infectious and parasitic disease category became less pronounced during the pandemic. While the reported numbers were always relatively low, alcohol-related mortality also declined during the pandemic.

## Introduction

The first cases of a novel coronavirus infection were initially reported in Wuhan, China in December 2019^1^. This virus was mainly spread by contact and aerosols airborne transmissions between humans^2^. After further investigation and metagenomic RNA sequencing^1^ this virus was proved to be 79% identical to the previously known SARS-CoV^3,4^. Therefore, the new virus was named SARS-CoV-2^5^ while the corresponding disease became known as COVID-19. The World Health Organization (WHO)^6^ declared a COVID-19 pandemic on March 11, 2020 just 3 months after SARS-CoV-2 was initially detected. As the pandemic wore on the virus spread from region to region and country to country at varying rates, impacting some countries more than others^7^. For example, the first case of COVID-19 in Western Europe was confirmed on January 24, 2020^8^ while the first confirmed case in Ukraine was reported on March 3, 2020^9^. The epidemic burden in various countries was also different for multiple reasons such as epidemic intensity^7^, overall preparedness of the authorities^10^ and scope of implemented public health measures.

Prior to development and implementation of effective vaccines and therapeutic regimens, mandatory non-pharmaceutical interventions (NPIs) constituted the main strategy to control the pandemic and to reduce the number of cases^11^. The introduced NPIs had a goal to limit the virus transmissions and included social distancing, self-quarantine, travel restrictions, mandatory or recommended mask-wearing in public places and even complete or partial lock-downs of non-essential public services and business activities for a period of time^12^. NPIs were used separately or in combination with each other with the expectation to limit transmission and reduce the number of COVID-19 cases^11^.

Typically, symptomatic COVID-19 patients present with a respiratory syndrome including fever, shortness of breath, cough, and viral pneumonia in severe cases^13^. The diagnostics of such severe infections and related deaths are straightforward to perform and are well-reported. The infections however, are not always severe or even symptomatic. Those mild or asymptomatic individuals at the same time can be both affected by the virus as well as to infect the others^14,15^. The corresponding individuals with very mild or no symptoms, however, typically remain undiagnosed and hence underreported.

As the pandemic progressed beyond early 2020, multiple new variants of SARS-CoV-2 emerged due to the evolution of the virus^16,17^ and, despite the NPIs, successful development of multiple vaccines^18^ and corresponding mass vaccinations, worldwide transmission has continued with multiple waves^7^ as of September 2022.

In summary, the COVID-19 transmission and progression within populations comprises a complex phenomenon which is affected by multiple aspects such as NPIs, viral evolution, vaccination campaign intensities, environmental seasonality^19–21^ and possibly other factors. The studies of all those aspects over a period of 2 years is difficult, especially if the comparison of the epidemic burden between regions with different populations is desired. In this situation, comparing “overall or all-cause mortality” in a given region before or during the pandemic and comparison between the two can be very informative. Once standardized by the corresponding population sizes, the corresponding mortality counts can also be compared between regions or countries with different populations.

In this work the epidemic burden in Ukraine was studied via excess mortality for a period of 2 years (2020–2021). Since the beginning of the pandemic, multiple regions of the world as well as some specific countries have been studied for the various impacts that the pandemic has caused. However, the attention that has been given to Eastern Europe in general and Ukraine in particular has been limited, even though it is the 2nd largest European country by area and 7th largest by the population. The available Ukrainian studies included general epidemic response studies^22,23^, disease transmission modeling for case counts for different stages^9,24–26^, genomic epidemiology analyses^9,27–29^, as well as the COVID-19 vaccination campaign studies including relevant limitations such as vaccination hesitancy^30,31,31–34^. The overall impact of the pandemic in Ukraine as determined via excess mortality studies has only been done in the global context with no country specifics or only during the early pandemic stages^35–37^. The aim of this work is to complement the global excess mortality studies and to fill the possible COVID-19 knowledge gaps for Ukraine.

Despite the relatively high level of sociodemographic and economic uniformity, the region of Eastern Europe is characterized by a significant diversity of implemented anti-COVID measures^22,38^. In this context, the Ukrainian COVID-19 pandemic is characterized by a number of unique features. In particular, Ukraine introduced strict NPIs from the very beginning of the pandemic on March 12, 2020^22,24^. The implementation and strictness of those NPIs, however, varied regionally within the country^39,40^ including the potential lack of compliance^41^. The vaccination campaign in Ukraine at the same time had a limited scope and by February 2022 only about 34% of the Ukrainian population had been fully vaccinated^42^. There were multiple reasons for that. In particular, The COVID-19 mass vaccination campaign was initially announced on February 1, 2021^34^ and officially started on February 24, 2021^43–45^. This was much later than in many other European countries^34^. The other reason has been a widespread vaccine hesitancy in the Ukrainian population which slowed down the pace of the country’s COVID-19 vaccination program^30–33^.

It is also worth noting that the reporting frequency, level of details and availability of COVID-19 incidence and related mortality data together with the all-cause and other cause-specific mortality data available regarding Ukraine has been superior in comparison to some other neighboring countries^9,46^. That allows a more thorough analysis of the all-cause as well as cause-specific excess mortality during the first 2 years of the COVID-19 pandemic in Ukraine^47,48^. The obtained mortality statistics provided a robust measure of the epidemic burden over the course of the pandemic and can be aligned in time to other pandemic-related events happening at the same time in Ukraine. In particular, this work presents: (1) the analysis of the all-cause excess mortality over the epidemic period in terms of standardized scores obtained from parametric and nonparametric models, (2) the detailed analysis of the cause-specific excess mortality by the available reported death categories, (3) the incorporation of aging characteristics of the Ukrainian population into the considered models to study their effect on the mortality trends, and (4) the studies of the auxiliary Google Trends mortality-related data queries and their relationship with the actual mortality data during the pandemic period.

## Data sources

### Mortality and demographics

The all-cause and cause-specific monthly mortality data from January 2015 until December 2021 from the State Statistics Service of Ukraine^47^ were aggregated by the third-party web service MinfinMedia (Minfin.com.ua)^48^; the latter provides an interface allowing for convenient retrieval and processing of data. The mortality causes were available for 34 categories. The confirmed COVID-19-specific mortality data (i.e. with the assigned code U07.1^49^ according to the ICD-10 classification^50,51^) formed a separate subset category within the all-cause mortality during each pandemic period^48^. The following causes of mortality (with the corresponding ICD-10 codes^50,51^) were also available for the analysis (after translated from the Ukrainian and Russian languages): infectious and parasitic diseases, with two subcategories: tuberculosis (A15–A19) and AIDS caused by HIV (B20–B24); tumors with “malignant” subcategory (C00–C97); blood and marrow diseases; endocrine diseases with diabetes (E10–E14) subcategory; mental and behavior diseases, including “alcohol-induced” (F10) category; diseases of nervous system; diseases of the circulatory system, including coronary artery disease (I20–I25), alcoholic cardiomyopathy (I42.6) and cerebrovascular disease (I60–I69) subcategories; respiratory system diseases with “flu and pneumonia” (J10–J18) subcategory; digestion system diseases and cirrhosis (K70) category; skin diseases; diseases of the musculoskeletal system; diseases of the genitourinary system; pregnancy and childbirth complications; perinatal conditions; birth defects and chromosome anomalies; symptoms not included in previous categories; outer causes with following subcategories: traffic accidents (V01–V99), drowning (W65–W74), fire and smoke (X00–X09), poisoning (except alcohol poisoning) (X40–X44, X46–X49), alcohol poisoning (X45), suicide (X60–X84), and violence (X85–X99, Y00–Y09).

The annual demographics data which included the population size and the age structure at the beginning of each year were downloaded from the Ukrainian Population Census website^52^.

No individual level data were collected or used in this study. Only aggregate population counts available over time were used with no personal information.

### Google trends

The Google Trends data^53^ contained monthly search query summaries of specific keywords monthly over the study period. The trends data provided auxiliary information about the individual’s search preferences and allowed observation of changes in such preference over time during the pandemic. Therefore, Google Trends service forms an additional source of data to get more details on epidemic progression and social impact over the two year study duration. For the Google Trends data analysis search queries in Ukrainian and Russian languages were used. In particular, the following keywords and key phrases were considered: (1) “ труна” (“truna”) and “ гроб” (“grob”)—Ukrainian and Russian keywords for coffin, (2) “поминки” (“pomynky”) and “поминки” (“pominki”)—Ukrainian and Russian keyword for memorial services and 3) “ритуальні послуги” (“rytualni posluhy”) and “ритуальные услуги” (“ritualnie uslugi”)—Ukrainian and Russian key phrases for funeral services. The individual queries data were obtained using geographic filter “Ukraine” for the proprietary Google Trends interface^53^.

## Methods

### Excess mortality

The *excess mortality* during the pandemic for a given calendar period (i.e. month) is defined as the difference between the reported mortality during that period and the “predicted” mortality for the same calendar period based on the historic data that preceded the pandemic. The predictions based on the historic data may or may not include the demographic characteristics of the population based on the availability of such data and based on whether the model used for prediction can incorporate such data^54^. The Ukrainian population has been aging during the study period^55^ and the aging demographics trend data were incorporated into the specific modeling framework^46^.

### Standardized scores

While excess mortality value for a given period is certainly a useful indicator of the epidemics impact for a given region, it does not allow the direct comparison of such excesses between regions with different populations. To approach this problem, the excess mortality is standardized to allow comparisons between regions with various population sizes and corresponding differences in mortalities. In particular, the *standardized scores* called *P*-scores^54,56^ are used for that purpose. Those statistics allow the comparison of not only regions with different population sizes, but also comparison of different time periods within the same region after the population change. There are two main ways to define the scores: nonparametric and parametric^46,54^. In this study, both versions of the score are used to compare mortalities between regions and also over time.

The nonparametric score $P(t_i)$ for a given period $t_i$ across $i=1,2,\dots ,I$ distinct time periods is defined as:

1 $$\begin{aligned} P(t_i) = \frac{x(t_i) - \bar{x}(t_i)}{\bar{x}(t_i)}\, \text{ for } \, i=1,2, \dots , I, \end{aligned}$$

where $\bar{x}(t_i)$ is the averaged mortality from analogous time periods within a year computed from the past *n* years which precede the time period of interest $x(t_i)$.

The parametric score $\mathscr {P}(t_i)$ for a given period $t_i$ across $i=1,2,\dots ,I$ distinct time periods is defined as:

2 $$\begin{aligned} \mathscr {P}(t_i) = \frac{x(t_i) - \hat{\mu }(t_i)}{\hat{\mu }(t_i)}\, \text{ for } \, i=1,2, \dots , I, \end{aligned}$$

where $\hat{\mu }(t_i)$ is the expected mortality for the time period $t_i$ which is predicted by a selected parametric model using *n* years of data before the period of study.

The described scores (1, 2) are not limited to the all-cause mortality data and can also be applied to any other auxiliary data. In this work, both parametric ($\mathscr {P}$) and nonparametric (*P*) scores are calculated for auxiliary data which include cause-specific mortality series and Google Trends query data. The score values are typically presented as percentages for easier interpretation.

In the presented analysis different approaches for modeling of the mortality counts series and estimation of the scores were utilized for the same data. The ultimate goal of using multiple approaches was to investigate how variable and comparable the modeled counts and estimated scores were *between* the considered approaches. This allows to evaluate the agreement between various methods.

The Google Trends search queries data are studied in the presented analysis solely as the *complement* to the original mortality data. Such search queries data are *always* available within a very short period of time with no delays in reporting. This can go in a sharp contrast with mortality data which may only be available with a long delay in reporting or not available at all^46^. As a result, the quick comparisons between two data times series (i.e. mortality and a search query) allows to study the corresponding associations and whether such trends can be used as a source of information when the actual mortality counts are not (yet) available.

### Prediction models

The mortality trend over time comprises a complex phenomena which is affected by multiple factors. The noticeable factors include seasonality of mortality during the calendar years^57,58^ as well as the long-term trends such as changing population structure over time^55^ which both at least have to be accounted for. In that regard, the aging of the Ukrainian population is particularly important for the COVID-19 pandemic analysis since people in the $65+$ age group are especially vulnerable to the disease^46,59^.

For this study, two known and previously used time series models^46^ were chosen for comparison purposes. Each incorporates *both* annual seasonality and aging population dynamics. These models are the well-established autoregressive integrated moving average (ARIMA) model^60^ as well as the more recent Prophet model for time series data^61^. Both models were fitted to the Ukrainian all-cause and available cause-specific mortality data prior to the pandemic and used for predictions during the pandemic period for the computation of the standardized parametric $\mathscr {P}$-scores (2). Two versions of each model were considered for comparison purposes i.e. with and without the number of individuals in the elderly age group (i.e. $65+$) to check the importance of such a predictor for the mortality counts. The analogous computations were performed individually for all six available Google Trends query series but only without demographic characteristics i.e. to those time series only. The R programming language for statistical computing^62^ was used for the entire analysis and the corresponding analysis code has been made publicly available online^63^.

The autoregressive integrated moving average (ARIMA) model is an established tool for time series analysis^64^. The model is an extension of the autoregressive (AR) and autoregressive moving average (ARMA) models and is characterized by three sets of parameters with sizes (*p*, *d*, *q*) that define the model. The parameter *p* controls the amount of previous series values $Y(t-1), Y(t-2), \dots , Y(t-p)$ which precede the current value *Y*(*t*) and which are used to model (predict) the current value i.e. the model lag parameter. If the modeled data are non-stationary, the ARIMA model cannot be applied to time series data directly, but rather to differences between the original series values^64^. The parameter *d* defines the order of such differences, meaning how many times the original series has to be subtracted so that for the resulting data the stationarity assumption can be utilized. For example, for $d=0$ no differences are taken i.e. the fitted value is $y(t) = Y(t)$, for $d=1$ it will be $y(t) = Y(t) - Y(t-1)$, for $d=2$ the fitted value will be $y(t) = \Big [ Y(t) - Y(t-1) \Big ] - \Big [ Y(t-1) - Y(t-2) \Big ]$, and so on. The parameter *q* defines the number of moving averages i.e. the size of the set of parameters which allow to capture periodicity within the studied data. The precise ARIMA model formulation has the form:

3 $$\begin{aligned} y(t) = \mu + \sum _{i=1}^{p} \varphi _i\, y(t-i) + \sum _{j=1}^{q} \theta _j\, \varepsilon _{t-j} + \varepsilon _{t}, \end{aligned}$$

where $\mu$ is the overall mean parameter, $\varphi _i$ for $i=1,2,\dots , p$ are autoregressive coefficients, $\theta _j$ for $j=1,2,\dots , q$ are moving average parameters and $\varepsilon _{t-j}$ for $j=0,1,2,\dots , q$ are errors for *q* preceding observations for the current time *t*. The ARIMA implementation from the forecast package in R^65^ was used for the analysis.

The Prophet model is a more recent development which has been proposed as a specialized tool for time series predictions and released by Facebook’s Core Data Science team^61^. In particular, this model has also been successfully used for COVID-19 pandemic studies^66,67^. The model contains three components which are expected to capture multiple processes which are happening in the data over the studied period. The captured processes include the non-periodical changes over time (i.e. data overall trend) and denoted as *g*(*t*), periodic changes over time (i.e. seasonal, monthly or weekly trend) and denoted by *s*(*t*) as well as irregular changes (i.e. holidays) are accommodated as *h*(*t*). The precise model formulation has the form:

4 $$\begin{aligned} y(t) = g(t) + s(t) + h(t) + \varepsilon _t, \end{aligned}$$

where $\varepsilon _t$ is the modeling error at time *t*. The Prophet model implementation was used from the original prophet package in R^61,68,69^.

### Standardized scores calculation

The nonparametric *P*-scores (1) were calculated based on the averaged monthly values $\bar{x}(t_i)$ for each calendar month $i=1,2,\dots ,12$ from 2015–2019 data. No historic data prior to 2015 were used, since in 2014 the Ukrainian government lost control over some of its territories^70^ and the corresponding population that lived there; as a result, the prior data would not be comparable with the later data. The same time interval (January 2015–December 2019) and the same approach have been used for each Google Trends query time series dataset.

The parametric $\mathscr {P}$-scores (2) were calculated based on the model-predicted values $\hat{\mu }(t_i)$ for $t_i$ $i=1,2,\dots ,22$ which predicted the 22 months of the pandemic years (from March 2020 to December 2021). The scores were not only computed from the pandemic years but also for the period from January 2015 to December 2021 for visualization and comparison. For the prepandemic time period model fits were used for $\hat{\mu }(t_i)$-s for $i=1,2,\dots ,62$ monthly periods.

Each model was fitted with and without the age-adjustment covariate which resulted in total *four* predicted values $\hat{\mu }(t_i)$ and the corresponding $\mathscr {P}(t_i)$ for each considered month $i=1,2,\dots ,74$ of the studied period. For the Prophet model fitting the default package settings were used for model fitting for both scenarios. For the ARIMA model fitting the following set parameters (12, 0, 12) were used for (*p*, *d*, *q*) to account for annual seasonality of mortality^57,58^.

### Google trends and mortality relationship

The mortality time series data are individually compared with each available Google Trends query series data to identify the possible associations between those series. The ultimate goal is to inspect whether the studied Google Trends data can serve as an indirect source of information about mortality trends during COVID-19.

To study such associations, all time series data were independently “standardized” by dividing each series by the median values of that series during the studied period (January 2015–December 2021) and by multiplying by 100 for easier comparison. For the resulting series smoothed analogs were also produced for easier comparison and noise removal using the loess build-in function in R with parameter span set to 0.25.

The resulting time series were compared both empirically via visualizations and more formally using the Granger causality test *with dependent* records^71,72^ using lmtest package in R.

## Results

### Raw data visualization

**Figure 1:**
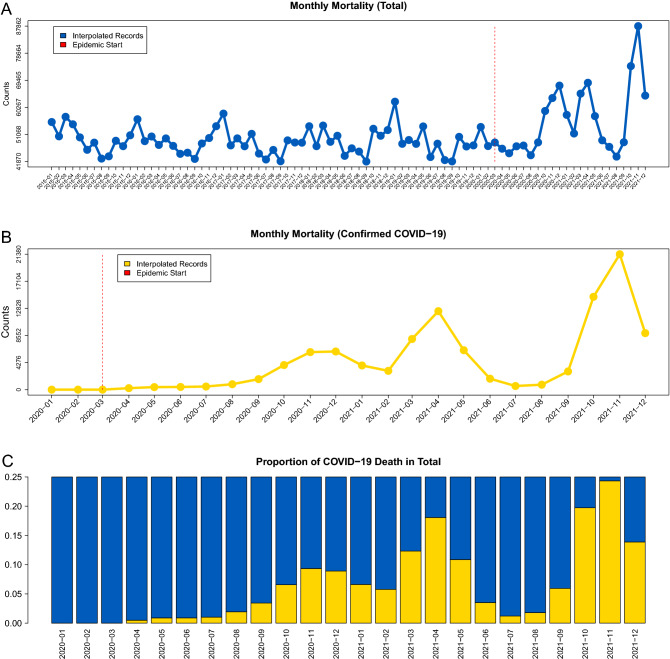
The visual summaries of the raw data for the all-cause and COVID-19-related mortality: (**A**) total monthly death counts joined by a linear interpolator. The start of the epidemic is marked with a red vertical line. (**B**) Confirmed COVID-19-related monthly deaths counts joined by a linear interpolator. The start of the epidemic is marked with a red vertical line. (**C**) Monthly proportions of the confirmed COVID-19-related deaths among the total deaths.

The confirmed COVID-19-related monthly mortality counts and the all-cause monthly mortality count for 2015–2020 are presented in Fig. 1 panels A and B, together with their corresponding proportional overlap (panel C). In particular, for the all-cause mortality there are three distinctive “peaks” in December 2020, April 2021 and November 2021 and those peaks occur at the same time as the COVID-19 mortality peaks (panels A and B in Fig. 1). Panel C from Fig. 1 illustrates that confirmed COVID-19-related mortality reached a maximum all-cause monthly death count of 25% in November, 2021. However, the all-cause mortality counts in November 2021 increased substantially more than 25% from historical prepandemic November counts. Thus, the increase in the all-cause mortality during the COVID-19 pandemic is only partially explained by the confirmed COVID-19-related mortality.

The analogous visual summaries of the raw data from the selected Google Trends queries are presented in Supplementary Fig. S1 (the Ukrainian language) and Fig. S2 (the Russian language). The query data had high variability as well as some differences between the data from each selected query.

### All-cause mortality nonparametric *P*-scores

**Figure 2:**
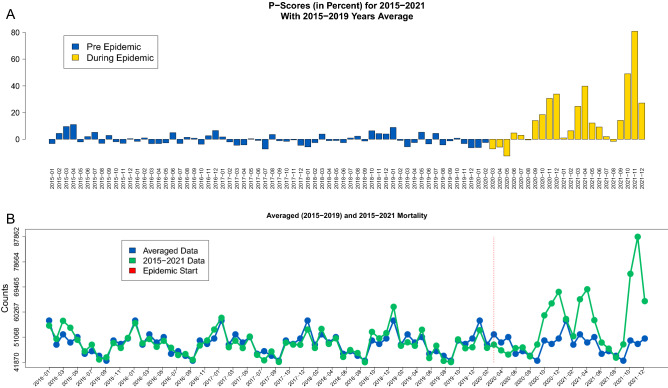
(**A**) Nonparametric all-cause mortality *P*-scores where prepandemic scores are colored in blue and during pandemic scores are colored in yellow. (**B**) Raw counts during 2015–2021 (green) together with the averaged all-cause monthly mortality computed based on 2015–2019 data (blue). The vertical red line in panel (**B**) indicates the start of the pandemic.

The values of nonparametric *P*-scores for all-cause mortality from January 2015 until December 2021 are presented in panel A of Fig. 2 where prepandemic scores are colored in blue and during pandemic scores are colored in yellow. The corresponding raw counts (green) during 2015–2021 together with the averaged monthly all-cause mortality (blue) computed based on 2015-2019 are presented in panel B of Fig. 2. The largest values of *P*-scores are observed during the three peak mortality months (December 2020, April 2021 and November 2021) as in Fig. 1. In particular, the highest value of *P*-score was observed in November 2021 with the value 81%.

The analogous visual summaries of the nonparametric *P*-Scores for the Google Trends queries are presented in the Supplementary Figs. S3 (the Ukrainian language) and S4 (the Russian language). For the Ukrainian language, there was a general increase in mortality-related searches (Supplementary Fig. S3), however, such growth has not been universal across all trends. For the coffin (i.e. “truna”) search query the significant increase has been observed across the pandemic period except in May 2021 while the maximum increase has been observed in September 2020–April 2021. For memorial service (i.e. “pomynky”) search query variability was high with no clearly pronounced trend. The noticeable increases have been observed in January 2021 and in May 2021. For the funeral services (i.e. “rytualni posluhy”) search query there was a uniform increase in searches with peak times around April–May 2021. For the Russian language, there was a general increase in mortality-related searches (Supplementary Fig. S4), however, such growth was not universal across all trends. For the coffin (i.e. “grob”) search query the significant increase was observed in April 2020 with a slow decay in the following months. For memorial service (i.e. “pominki”) the results were identical to the Ukrainian language since this word is spelled the same in the Cyrillic alphabet for both languages, with no possibility to distinguish between the two due to the same spelling i.e. the peaks were observed in January 2021 and in May 2021. For funeral services (i.e. “ritualnie uslugi”) search query there was a uniform increase in searches with peak times in June 2020 then April and October of 2021.

### All-cause mortality parametric $\mathscr {P}$-scores

**Figure 3:**
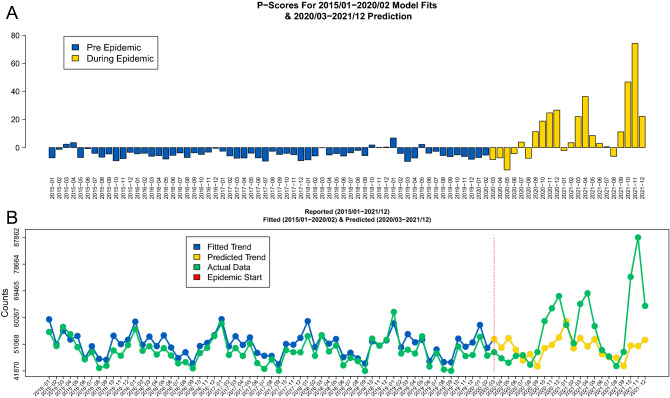
(**A**) Parametric all-cause mortality $\mathscr {P}$-scores based on the Prophet model *without* demographic characteristics where prepandemic scores are colored in blue and during pandemic scores are colored in yellow. (**B**) Raw counts during 2015–2021 (green) together with the fitted (blue) and predicted during the pandemic (yellow) values by Prophet model *without* demographic characteristics. The vertical red line in panel (**B**) indicates the start of the pandemic.

The parametric $\mathscr {P}$-score values produced by either the Prophet model or the ARIMA model with or without the demographic (i.e. 65+ covariate) produced very similar results. In particular, the predictions for 2020–2021 from the Prophet model fitted to 2015–2019 *without* the demographic (i.e. 65+ covariate) and the corresponding parametric $\mathscr {P}$-scores are summarized in Fig. 3. While the fitted values slightly overestimate the actual counts (panel B of Fig. 3) the trend is well captured and the corresponding predictions during the pandemic times are significantly below the pandemic all-cause mortality maximums. The corresponding all-cause mortality parametric $\mathscr {P}$-scores are up to 80% higher than expected for certain months (i.e. in November 2021).

The analogous visual summaries for the Prophet model with the demographic characteristic (i.e. 65+ covariate) as well as for ARIMA model with or without the demographic characteristic (i.e. 65+ covariate) are summarized in the Supplementary Figs.  S5, S6, and S7. The comparisons of each model fits and predictions with and without demographic characteristics are also provided in the Supplementary Fig. S8 for the Prophet model and in Fig. S9 for the ARIMA model. While the Prophet models seem to provide a slightly better visual fit, the model comparisons indicate that the models with and without demographic characteristics produce very similar model fits and predictions. Consequently, the incorporation of the demographic changes does not affect the model and is not an important factor in explaining the mortality increase during the pandemic in Ukraine.

The visual summaries of the parametric $\mathscr {P}$ scores for the Google Trends queries are presented in the Supplementary Fig. S10 (the Ukrainian language) and S11 (the Russian language). Both for the Ukrainian and the Russian languages, there has been a general increase in mortality-related searches (Supplementary Figs. S3, S4). Such growth, however, has not been universal across all trends. Such growth was less pronounced in the parametric models and the corresponding parametric scores than in the analogous non-parametric estimates and corresponding scores. This likely happened because of high variability in trends data across queries and across years within each query and the slight overestimation of the actual historic trend by the parametric models (Supplementary Figs. S3,  S4).

### Estimated excess of all-cause mortality by different models

While the models were very similar in their score estimates, the Prophet model captured the data seasonality and variability in the best way based on the visualizations of the model’s fits. For a numeric comparison, the estimated monthly summaries of the all-cause excess mortality counts for the utilized nonparametric and parametric approaches have been grouped in Table 1 for 2020 and in Table 2 for 2021. The summaries for each modeling approach are provided in a separate row of the corresponding table. The estimated excess in all-cause mortality counts in those tables is defined as the actual observed counts minus model-predicted counts and can be either positive or negative. In particular, negative estimates indicate the *deficiency* in observed mortality counts in comparison to the model-predicted (i.e. expected) mortality counts for a given month. In the same way, positive estimates indicate the *excess* in observed mortality counts in comparison to the model-predicted (i.e. expected) mortality counts for a given month. The corresponding values of such estimates quantify the values of deficiency or excess in mortality for that given month. The last table columns indicate the annual estimates (i.e. sums of positive and negative estimates across all months) for a given year.

The presented summaries allow to quantify how *different* the predicted mortality excess counts are based on the selected approach. While the utilized methods are different, the presented results are comparable. In particular, while the monthly all-cause mortality for pre-COVID-19 years in Ukraine mostly varied in the range from 40 to 60 thousand^48^ the disagreements between the model predictions were in a much smaller range. For those months, when the excess mortality was the largest (i.e. November and December of 2020 and March, April, October, November and December of 2021) the predicted numbers were fairly close to each other. The annual numbers were especially close for ARIMA and non-parametric approaches while for the Prophet model they were slightly lower. This indicates that the corresponding mortality scores will be relatively robust and do not depend that much on the selected approach.

**Table 1:** The estimated excess in all-cause mortality counts (i.e. actual counts-model-predicted counts) for 2020 based on the used modeling methods.

Method	Jan	Feb	Mar	Apr	May	Jun	Jul	Aug	Sep	Oct	Nov	Dec	Total
Nonparametric	− 3534	− 1184	− 3760	− 2881	− 6416	2085	1373	− 222	5942	9205	14,863	17,099	32,569
Prophet	− 4141	− 2711	− 4578	− 3417	− 8570	− 2048	1909	− 3477	4765	9408	12,767	14,180	14,087
Prophet & 65+	− 4040	− 2740	− 4295	− 3349	− 8282	− 1888	1930	− 3401	4884	9341	12,656	14,244	15,061
ARIMA	− 611	172	− 4680	− 2171	− 7395	4158	− 766	− 1105	− 280	9474	15,521	18,806	31,123
ARIMA & 65+	− 1674	576	− 2923	− 2232	− 5971	5635	− 1979	776	840	8460	15,820	18,397	35,726

Negative values indicate that observed mortality counts were less than predicted counts.

**Table 2:** The estimated excess in all-cause mortality counts (i.e. actual counts-model-predicted counts) for 2021 based on the used modeling methods.

Method	Jan	Feb	Mar	Apr	May	Jun	Jul	Aug	Sep	Oct	Nov	Dec	Total
Nonparametric	577	3079	12,852	19,517	6197	4119	927	− 692	5995	24,440	39,287	13,700	129,997
Prophet	− 1180	1610	11,691	18,390	4618	1665	361	-3203	4686	23,767	37,543	11,726	111,675
Prophet & 65+	− 1096	1762	11,838	18,568	4546	1492	390	− 3016	4430	23,673	37,465	11,693	111,744
ARIMA	4235	2524	13,187	23,045	5917	4816	− 1433	− 1336	− 174	25,478	39,125	14,942	130,325
ARIMA & 65+	4020	2809	14,316	21,628	7899	6264	− 2439	479	436	24,635	39,595	14,802	134,445

Negative values indicate that observed mortality counts were less than predicted counts.

Similarly to Tables 1 and 2, the corresponding summaries of nonparametric (*P*) and parametric ($\mathscr {P}$) scores for the utilized methods are grouped in Table 3 for 2020 and in Table 4 for 2021. The summaries for each modeling approach are provided in a separate row of the corresponding table. The scores are derived from the estimated excess in all-cause mortality counts from Tables 1 and 2 and can also be either positive or negative. In particular, negative scores quantify by what percentages there is a *deficiency* in observed mortality in comparison to the model-predicted (i.e. expected) mortality for a given month. In the same way, positive scores quantify by what percentages there is an *excess* in observed mortality in comparison to the model-predicted (i.e. expected) mortality for a given month. For example, for all approaches the largest scores were observed for December of 2020 and April and November of 2021. The largest scores were in the range from 74.34% to $82.03\%$ indicating the corresponding estimated excess in mortality above the expected values.

**Table 3:** The values of parametric ($\mathscr {P}$) and nonparametric (*P*) scores for 2020 based on the used modeling methods.

Method	Jan	Feb	Mar	Apr	May	Jun	Jul	Aug	Sep	Oct	Nov	Dec
Nonparametric	− 6.18	− 2.45	− 7.22	− 5.87	− 12.56	4.64	2.99	− 0.50	14.00	18.47	30.60	33.82
Prophet	− 7.29	− 5.65	− 8.58	− 6.93	− 15.71	− 4.16	3.63	− 7.84	10.96	18.99	24.81	26.74
Prophet & 65+	− 7.01	− 5.49	− 8.16	− 6.76	− 15.64	− 3.86	4.25	− 7.17	11.23	18.79	24.92	26.67
ARIMA	− 1.13	0.37	− 8.83	− 4.49	− 14.20	9.69	− 1.59	− 2.45	− 0.57	19.11	32.39	38.49
ARIMA & 65+	− 3.03	1.24	− 5.70	− 4.61	− 11.79	13.61	− 4.02	1.79	1.77	16.72	33.22	37.34

Negative values indicate that observed mortality counts were less than predicted counts.

**Table 4:** The values of parametric ($\mathscr {P}$) and nonparametric (*P*) scores for 2021 based on the modeling methods used.

Method	Jan	Feb	Mar	Apr	May	Jun	Jul	Aug	Sep	Oct	Nov	Dec
Nonparametric	1.01	6.37	24.68	39.75	12.13	9.16	2.02	− 1.56	14.13	49.03	80.88	27.09
Prophet	− 2.06	3.44	22.16	36.98	8.72	3.41	0.74	− 6.62	10.65	47.03	74.75	21.80
Prophet & 65+	− 1.86	3.55	22.29	37.10	8.62	3.14	0.84	− 6.48	10.07	46.78	74.34	22.24
ARIMA	7.92	5.17	25.48	50.56	11.52	10.88	− 2.97	− 2.98	− 0.36	52.20	80.28	30.29
ARIMA & 65+	7.49	5.78	28.28	46.02	15.99	14.63	− 4.95	1.11	0.91	49.62	82.03	29.93

Negative values indicate that observed mortality counts were less than predicted counts.

In summary, regardless of the utilized approach (nonparametric (*P*) vs parametric ($\mathscr {P}$)) or the model choice (i.e. Prophet or ARIMA) the summaries indicate the excess in all-cause mortality during the pandemics with three peaks. The incorporation of age structure into the parametric $\mathscr {P}$-scores modeling did not have a significant impact on the mortality scores.

### Google trends and mortality relationship

The visual summaries of the standardized mortality counts versus the monthly standardized Google trends data together with the corresponding smoothers are presented in Supplementary Fig. S12 for the Ukrainian language and in Supplementary Fig. S13 for the Russian language. The summaries are provided for the entire studied period from January 2015 until December 2021 to evaluate the relationships between the over prepandemic and pandemic time periods. From the graphs there is some support for the hypothesis that Google Trends queries series are associated with the all-cause mortality count series.

The corresponding *p* values of the Granger causality test for the ability of standardized Google trends data to forecast the standardized all-cause mortality counts are presented in Supplementary Table S1 for the Ukrainian language and in Supplementary Table S2 for the Russian language. The *p* values for the standardized series are presented in the first columns ($p_{GR}$) of those tables and the corresponding *p* values for the standardized and smoothed versions of the series are presented in the second columns ($p_{GR(Sm)}$) of those tables. Overall, there is some evidence of the associations between the series for certain keywords and key phrases. In particular, there are such evidences for the standardized series for the Ukrainian queries coffin (i.e. “truna”, $p_{GR} = 0.07$), memorial service (i.e. “pomynky”, $p_{GR} <0.01$) and funeral services (i.e. “rytualni posluhy”, $p_{GR} = 0.03$) and the Russian query memorial service (i.e. “pominki”, $p_{GR} <0.01$). There are also such evidences for the standardized and smoothed series for the Ukrainian query of funeral services (i.e. “rytualni posluhy” $p_{GR(Sm)} < 0.01$) and the Russian query coffin (i.e. “grob” $p_{GR(Sm)} < 0.01$).

### Cause-specific mortality summaries

**Figure 4:**
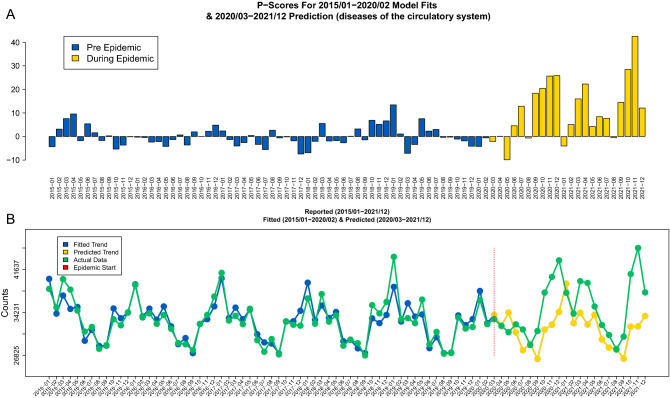
The visual summaries of parametric mortality $\mathscr {P}$-scores for circulatory system diseases (I20–I25) based on the Prophet model *without* demographic characteristics are presented on panel (**A**). The yellow bars represent the epidemic period. The corresponding Prophet model predictions (blue) based on the 2015–2020 data (panel **B**) are presented along the reported data (green). The vertical red line indicates the epidemic start period.

**Figure 5:**
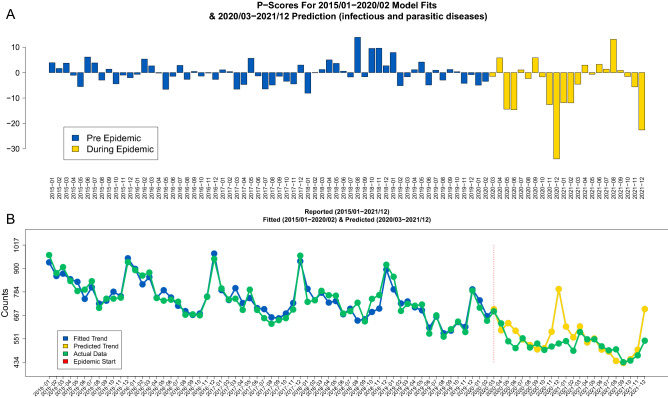
The visual summaries of parametric mortality $\mathscr {P}$-scores for infectious and parasitic diseases based on the Prophet model *without* demographic characteristics are presented on panel (**A**). The yellow bars represent the epidemic period. The corresponding Prophet model predictions (blue) based on the 2015–2020 data (panel **B**) are presented along the reported data (green). The vertical red line indicates the epidemic start period.

The visual summaries of the nonparametric scores (*P*) as well as parametric scores ($\mathscr {P}$) based on the Prophet model without the age covariates have been produced for each of the 34 total available cause-specific mortality categories (excluding the COVID-19 diagnosis). The visual summaries are provided in Figs. 4, 5 and the rest in Supplement. Based on those summaries there are indications that many of those cause-specific mortality series had a long increasing or decreasing trend during the studied period which are hard to capture by nonparametric scores which rely on averaged values only.

The most noticeable change was observed in the categories of flu and pneumonia as well as circulatory system diseases. For the respiratory system diseases category *including* its “flu and pneumonia” subcategory (J10–J18) there was a substantial increase (presumably due to COVID-19 infections and corresponding mortality) which is illustrated in Supplementary Figs. S63 and S64 respectively. The “flu and pneumonia” (J10–J18) specific mortality *only* also increased and is summarized in Supplementary Figs. S30 and S64. For the circulatory system diseases including its subcategories of coronary artery disease (I20–I25) and cerebrovascular disease (I60–I69) it is important to emphasize that mortality peaks over time were similar to the one of COVID-19 mortality with peaks in December 2020, April 2021 and November 2021. The last peak was also significantly higher than the previous two which also agreed with the COVID-19 mortality patterns. In particular, in Fig. 4 the parametric $\mathscr {P}$-sores are displayed along the actual data and the Prophet model fit and predictions.

The other observation is the decreasing trend for the “infectious and parasitic diseases” category (which does not include COVID-19) as well as for the corresponding “AIDS caused by HIV” (B20–B24) subcategory for which the parametric $\mathscr {P}$-sores are displayed along the actual data and the Prophet model fit and predictions in Fig. 5 and Supplementary Fig. S16.

As for the other categories, there was some decline in tuberculosis (A15–A19) mortality (Supplementary Fig. S49). Some decline was also observed in alcohol-related categories. For example, alcohol poisoning (X45) (Supplementary Fig. S45) “lost” its usual peaks around New Year’s Eve in December 2020 while alcohol-induced mental diseases (F10) (Supplementary Fig. S57) had declined substantially. Cirrhosis (K70) and alcoholic cardiomyopathy (I42.6) had slightly decreased, although mortality counts for both of those groups have been low and, consequently, such decline could be attributed to rare event effects (Supplementary Figs. S66, S61 respectively). Cancer-related mortality (C00–C97) slightly decreased during the pandemics (Supplementary Fig. S51) while digestive system disease mortality (Supplementary Fig. S65) exceeded the predicted values based on historic data. Mortality counts for digestive system disease was relatively low and, consequently, the observed decline could be attributed to rare event effects. The trends of other categories are either unchanged (other causes, not related to alcohol), or have high variability due to relatively low death counts for each such category which makes the corresponding conclusions less robust and reliable. The summaries for all available categories and subcategories are provided in the Supplement.

## Discussion

### Ukraine and its neighbors

For Ukraine the calculated nonparametric *P*-scores were similar to the corresponding parametric $\mathscr {P}$-scores and they had similar patterns with nonparametric *P*-scores in neighboring countries of Poland, Romania, Hungary and Moldova which were computed earlier and made publicly available^73^. At the same time, neighboring Russia and Belarus have different patterns of nonparametric *P*-scores over time. It is worth noting here, that Belarus stopped reporting its mortality soon after the pandemic started^46^ and as of September 2022 no all-cause mortality past June 2020 has been reported. The scores for Russia summarize mortalities over a huge geographical territory with highly variable populations and population densities which may limit the utility of the corresponding aggregate statistics. When comparing Ukraine with other neighbors, for example, one can see that Ukraine has a (substantial) increase in mortality during the COVID-19 waves similar to its neighbor Romania, where the proportion of vaccinated individuals has been one of the lowest in Europe as of October 2021^74^. This is in agreement with relatively low vaccination rates in Ukraine of 34–36%^42,75^ as well as with relevant vaccination hesitancy^31,34^. At the same time, neighboring Poland with the proportion of the vaccinated population equal to 60%^75^ has had downward *P*-score trends between the first and the third waves. Ukraine can also be compared to the more distant neighbors in Western Europe. In particular, Ukraine has been compared with the United Kingdom, Belgium, France, Spain, Italy and Sweden^76^.

The most noticeable difference is that the first “peak” of mortality (April 2020) in Western Europe was absent in Ukraine^73^. At the same time the third wave (November 2021) which was observed in Ukraine and among neighbors^73^ was absent in those Western European countries. There can be multiple reasons for that, such as different reactions of the governments in Eastern and Western European countries, which affected the first wave of the pandemic. At the same time, the absence of the third wave in the above-mentioned Western European countries in November 2021 could potentially be attributed to relatively high vaccination rates and general better preparedness of the corresponding healthcare system for the subsequent epidemic waves.

### Difference in model fits and predictions

Overall, the non-parametric scores (1), the ARIMA model (3) and the Prophet model (4) were considered to compare the results from multiple methods for the same data and to compare the modeled counts and the estimated scores between approaches. In summary, the results were comparable but the estimated all-cause excess mortality summaries for counts grouped in Table 1 for 2020 and (especially) in Table 2 for 2021 did show some variability. It is hypothesized that results are different due to the inherited structure of the used models. In particular, the Prophet model in comparison to the other approaches has an additional component for rare events (e.g. holidays) while the ARIMA model and the non-parametric scores do not include such functionality. This may explain the discrepancy in results between the Prophet model and the other methods for those months when the excess counts were relatively low. The results for the months with substantial excess were similar. The overall range of predicted counts between methods, however, was similar for all modeled and predicted months regardless of how large or small the counts were. The visual representations of the model fits were also consistent with the data.

### Mortality causes and associations

For the cause-specific mortality data, potential underlying associations between COVID-19 pandemics, public health response and mortality categories can be concluded. For example, there is a relationship between COVID-19 and the circulatory system diseases category^77^ that was noticeable in the corresponding trends (Supplementary Figs. S26, S25 and S60, S59). Additionally, mortalities in the flu and pneumonia category (Supplementary Figs. S30, S64) were linked in time to COVID-19 mortalities. This is expected due to the complicating sequelae associated with COVID-19 infections. The identified mortality decline in the “infectious and parasitic disease” category (which also includes “HIV related mortality”) may be surprising but for some diseases (*excluding* the HIV category) mandatory mask-wearing, increased sanitation, at level of the individual (e.g. hand sanitation) and the environment (e.g. constant disaffection of buildings and other facilities), and limited personal interactions likely restricted person-to-person transmission of all infectious diseases. The reduction in HIV death may be due to multiple causes such as worth reporting, decreased exposures for people with the compromised immune systems, reduced risk behavior, and reduced sexual interactions. The differences in alcohol-related death trends are likely due to the COVID-19 restrictions such as lockdown of bars^78^ and potential limitations of sales in stores. In summary, the analysis of the cause-specific mortality counts illustrates how those morality causes were changing during the pandemic period.

### Analysis limitations

There are a few limitations of the presented analysis inherited from the quality of available data. The first one, which is true for all-case and cause-specific mortality, being the potential delays in reporting of new data which are caused by the delayed aggregation of the all cause mortality data. This may happen, for example, due to not immediately known death causes and not immediately known death times which cause delays in reporting^56^. The ongoing conflict as of September 2022 in Ukraine could cause further inhibition of public health data release.

Monthly aggregated data counts and differences in reporting scales are additional limitations in the utilized datasets. While the COVID-19 related mortality for Ukraine was reported fairly frequently over the study period (daily), the all-cause and cause-specific mortality for Ukraine has only been reported monthly which limits the analysis scale. In addition to that, such scale differences in reporting frequencies are common *between* the countries which makes it difficult to compare all-cause and cause-specific deaths across regions^79^.

On top of that it should always be kept in mind that the cause-specific mortality data are always inferior to the all-cause mortality data in terms of accuracy regardless of the selected cause of death. This happens due to multiple reasons such as wrong diagnoses, undiagnosed death, and multiple death causes for the same individuals. Those discrepancies can either cause underreporting or overreporting and it is not surprising that the confirmed COVID-19 case-specific mortality contributed only a fraction of the all-cause mortality excess during the pandemic (panel C of Fig. 1) which was observed previously^46^. Therefore, all cause-specific mortality causes should be treated with caution especially for those causes which are not always apparent and may require additional laboratory diagnostic procedures.

It should be kept in mind that various demographic and social processes are happening constantly in every society. Those processes were happening before the pandemic and likely changed in major ways because of the pandemic. In particular, those factors could have ultimately increased or decreased the all-cause mortality during 2020–2021. The overall stress and relevant events which occurred during the pandemic time could have caused mental and physical health complications, which could have increased the mortality in Ukraine due to various causes which are unrelated to COVID-19. Also, the inability to routinely organize doctor’s visits and to conduct planned hospitalizations and surgeries could have had a similar effect. On the other hand some other factors have also been present which could have decreased the all-cause mortality, such as mass immunization of the population as well as the advancement of medical technology and medicine^80^. The limited mobility and travel also could have had a protective effect since various traumas and car accidents were less likely to occur. Therefore, the all-cause mortality within the presented study should be interpreted as the COVID-19 pandemic influenced mortality or the overall COVID-19 pandemic burden rather than being solely caused by the SARS-CoV-2 virus.

While the age characteristic has been incorporated into the utilized models due to its importance in explaining the COVID-19-related mortality^46,59^, there are plenty of other risk factors that could have affected the outcome. Those risk factors could have been various changes in social behavior or the acquired immunity over time or others for which reliable data are not available. For example, it has been documented that men are more likely to exhibit more severe COVID-19 symptoms than females^81^. These potential factors have not been analyzed due to either a lack of such data availability for Ukraine or the inability to conduct such studies with aggregate counts on the population level which the presented study has focused on.

The utilization of Google Trends search queries data is also not ubiquitous^82^. Although those data have been useful for comparing Ukrainian mortality trends, such data directly inherit self-reporting biases due to the constant variations in the number of active Google users and their search preferences. The search queries are also prone to sudden increases in popularity due to factors which are (potentially) unrelated to epidemics. For example, the Russian search keyword “grob” (i.e. coffin) which has been used as a Google Trends query has experienced a sudden increase starting March 2020. At the same time the increase in the all-cause mortality has not been reported in Ukraine during the same period so the increase could have been caused by other reasons^83^. It is also worth noting, that the potential set of key phrases related to mortality searches is endless which can cause selection biases for mortality trends. This has also been indirectly illustrated by the observed variability between the six chosen search queries. Therefore, Google Trends search data summaries should be interpreted as an auxiliary complement to the mortality data and the related summaries should be interpreted with caution. Such Google Trends search queries data, however, are always rapidly available within a very short period of time which allows to quickly perform the desired modeling and to evaluate the corresponding changes in trends. This may be handy as a source of auxiliary information when no data or limited data are available within the desired time frame^46^.

While the standardized *P*-scores are widely used for mortality analysis^46,54,56^, according to the authors knowledge, the presented work is the first thorough excess mortality analysis for Ukraine. The presented analysis evaluated *both* the all-cause mortality summaries, as well as case-specific mortality and Google Trends summaries using standardized *P*-scores. The presented analysis has also incorporated the demographic characteristics and investigated the effects of those. The only identified and published manuscripts on excess in all-cause mortality in Ukraine were in the context of global mortality^35,36^ with the limited focus on the country’s specifics. The other available Ukrainian manuscripts focused on COVID-19-specific incidence, mortality and genetics^9,24,84–86^, vaccinations^31^, environmental factors^87^ and other COVID-19 aspects^22,88,89^. The only discovered works about the excess mortality in Ukraine were two works for the year 2020^90,91^. In the first work^90^ (written in English) the authors used a linear regression modeling framework without temporal dependence and the monthly index as a predictor. The estimated excess mortality was 38, 095 for the entire year. In the second work^91^ (written in Ukrainian) the author used mortality indexes framework for different age groups. The estimated excess mortality was 10, 525 for the entire year. Such findings^90,91^ are comparable with the results presented in this manuscript for the year 2020. In particular, the first estimate is comparable to estimates from nonparametric (32,569) and parametric ARIMA (31, 123) and ARIMA & 65+ (35,726) models. The second estimate is comparable to the estimates from Prophet (14,087) and Prophet & 65+ (15,061) modes.

It is also important to acknowledge that the epidemiological situation in Ukraine should have been significantly altered in 2022 by the Russia-Ukraine military conflict which started on February 24, 2022 and is ongoing as of January 2023. It is having a substantial impact on the Ukrainian population through mass migration of refugees^92^, war-related injuries and mortality, economical situation and many other aspects which lead to public health crisis^93^. In particular, the proper diagnostics, identification, treatment and reporting of COVID-19 cases around Ukraine as well as implementation of prevention measures became challenging^94^. In addition to that, there were indications of increase in stress and anxiety^92,95^. There was also overwhelming pressure on the Ukrainian healthcare system due to war casualties, reduced access to primary medical treatment, damages to the socioeconomic system and many other factors^92^. The full impacts of those events on the population and epidemiological dynamics still have to be estimated, when the reliable data will become available^89,93,94,96^. At present, the analysis of these impacts is in progress due to the ongoing conflict and lies beyond the scope of this paper which had a focus solely on COVID-19 during pre-war years 2020–2021.

## Supplementary Information

Supplementary Information.

## Data Availability

The datasets generated and/or analyzed during the current study are available in the Github repository. The repository is available via the link: https://github.com/keder/ukraine_mortality_analysis.

## Code availability

The analysis source code in R has been made publicly available on Github^63^ under the MIT License^97^.
