# Supplementary material for: Excess mortality in Ukraine during the course of COVID-19 pandemic in 2020–2021
Source: Sci Rep. 2023 Apr 27;13:6917. doi: 10.1038/s41598-023-33113-2 (PMC10139669; doi:10.1038/s41598-023-33113-2)
Supplement: Supplementary file 1 — Supplementary Information. [file 41598_2023_33113_MOESM1_ESM.pdf]

Excess mortality in Ukraine during the course of COVID-19 pandemic in 2020-2021.

Aleksandr Shishkin[1], Pema Lhewa[1,+], Chen Yang[2,+], Yuriy Gankin[3], Gerardo Chowell[1], Michael Norris[4,5], Pavel Skums[6], and Alexander Kirpich[1,*]

[1]Department of Population Health Sciences, School of Public Health, Georgia State University, Atlanta, Georgia, United States of America
[2]Department of Biology, Georgia State University, Atlanta, Georgia, United States of America
[3]Quantori, Cambridge, Massachusetts, United States of America
[4]Department of Geography, University of Florida, Gainesville, Florida, United States of America
[5]Emerging Pathogens Institute, University of Florida, Gainesville, Florida, United States of America
[6]Department of Computer Science, Georgia State University, Atlanta, Georgia, United States of America
[*]akirpich@gsu.edu
[+]these authors contributed equally to this work

ABSTRACT

In this work the COVID-19 pandemic burden in Ukraine is investigated retrospectively using the excess mortality measures during 2020-2021. In particular, the epidemic impact on the Ukrainian population is studied via the standardized both all-cause and cause-specific mortality scores before and during the epidemic. The excess mortality counts during the pandemic were predicted based on historic data using parametric and nonparametric modeling and then compared with the actual reported counts to quantify the excess. The corresponding standardized mortality P-score metrics were also compared with the neighboring countries. In summary, there were three "waves" of excess all-cause mortality in Ukraine in December 2020, April 2021 and November 2021 with excess of 32%, 43% and 83% above the expected mortality. Each new "wave" of the all-cause mortality was higher than the previous one and the mortality "peaks" corresponded in time to three "waves" of lab-confirmed COVID-19 mortality. The lab-confirmed COVID-19 mortality constituted 9% to 24% of the all-cause mortality during those three peak months. Overall, the mortality trends in Ukraine over time were similar to neighboring countries where vaccination coverage was similar to that in Ukraine. For cause-specific mortality, the excess observed was due to pneumonia as well as circulatory system disease categories that peaked at the same times as the all-cause and lab-confirmed COVID-19 mortality, which was expected. The pneumonias as well as circulatory system disease categories constituted the majority of all cases during those peak times. The seasonality in mortality due to the infectious and parasitic disease category became less pronounced during the pandemic. While the reported numbers were always relatively low, alcohol-related mortality also declined during the pandemic.

Supplemental Material

Google Trends Raw Data

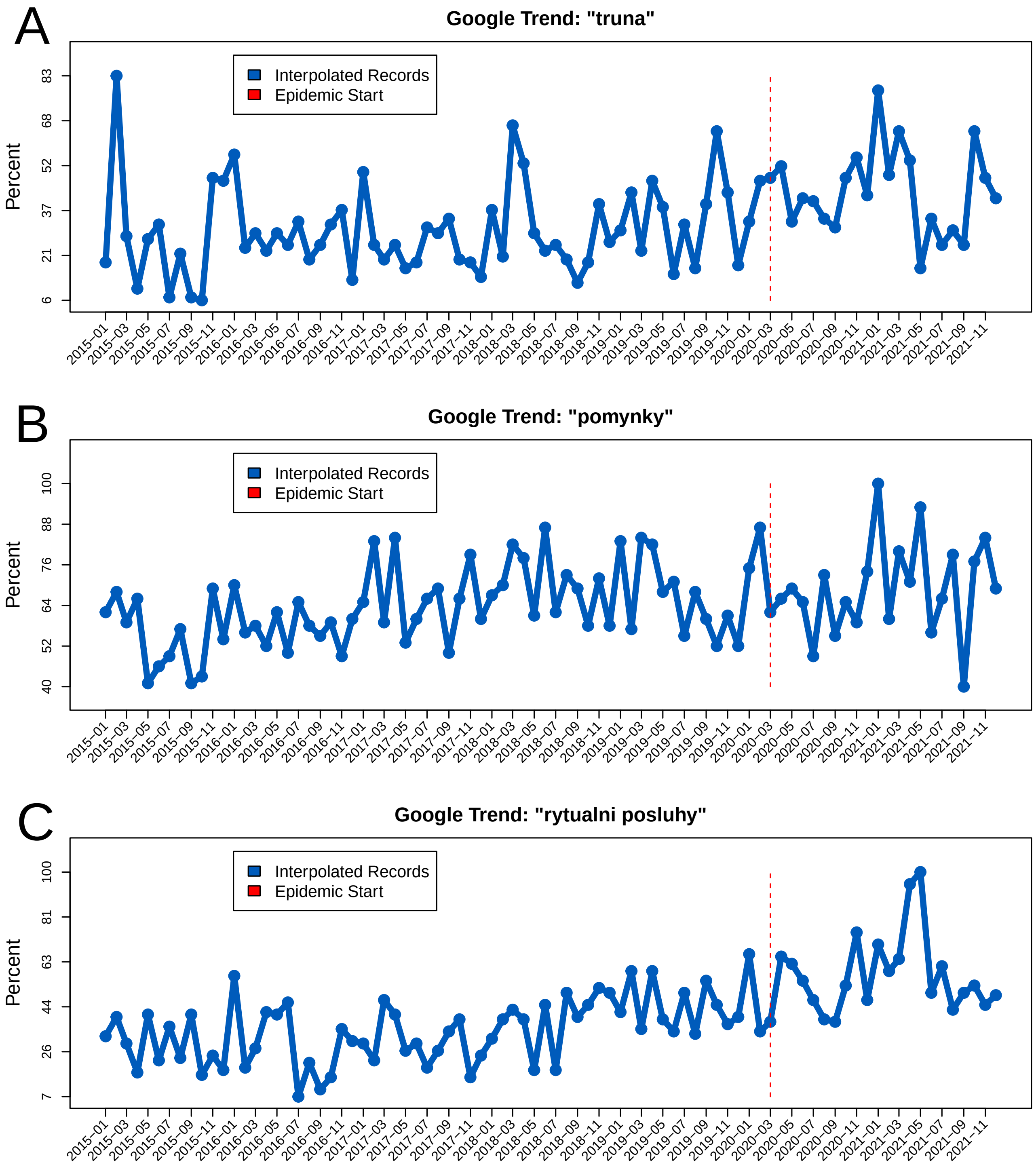

Figure S1. The visual summaries of the Ukrainian language monthly raw Google trends data from January 2015 until December 2021 for queries: “truna” meaning coffin (panel A), "pomynky" meaning memorial service (panel B) and “rytualni posluhy” meaning funeral services (panel C). The vertical red line demonstrates the start of the epidemic, based on the first confirmed case date.

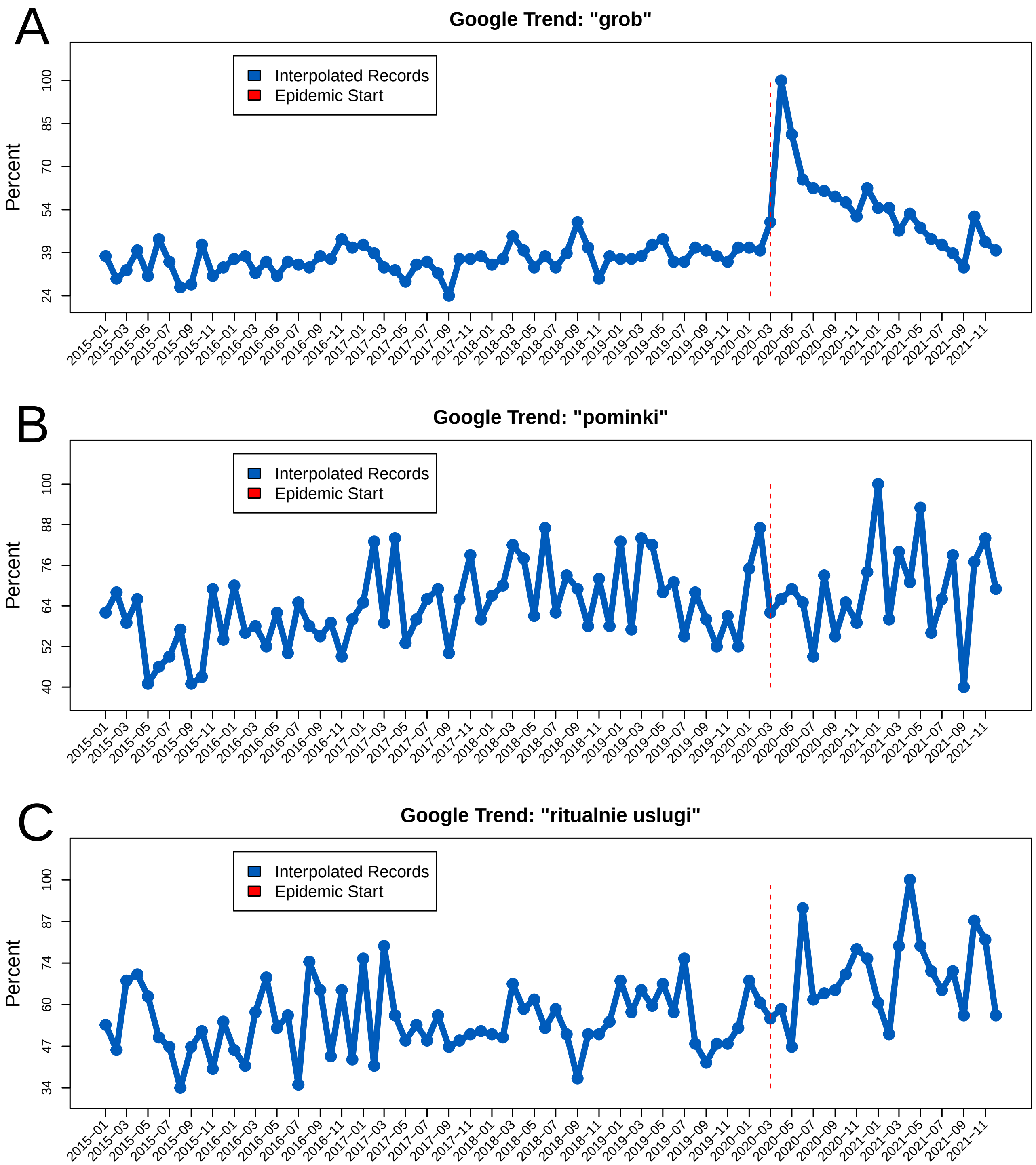

Figure S2. The visual summaries of the Russian language monthly raw Google trends data from January 2015 until December 2021 for queries: “grob” meaning coffin (panel A), "pominki" meaning memorial service (panel B) and “ritualnie uslugi” meaning funeral services (panel C). The vertical red line demonstrates the start of the epidemic, based on the first confirmed case date.

Google Trends Nonparametric P-Scores

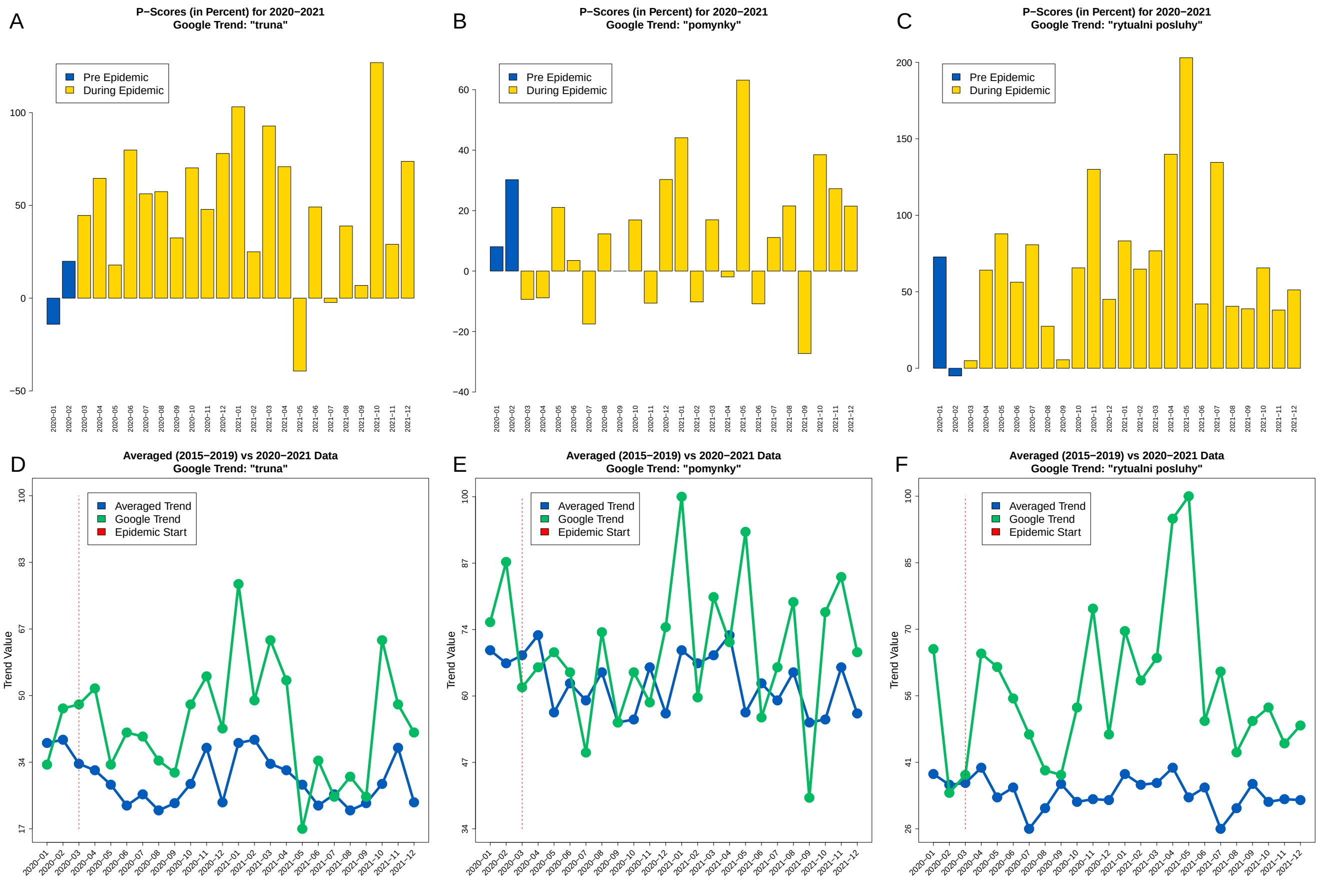

Figure S3. The visual summaries of the Ukrainian language nonparametric Google Trends searches P-scores are presented for "coffin" (panel A), "memorial service" (panel B) and "funeral services" queries (panel C), where yellow bars are used for the epidemic period. The corresponding averaged data based on *five* years (blue) are plotted together with the query data (green) for coffin (panel D), memorial service (panel E) and funeral services (panel F). The vertical red line indicates the epidemic start period.

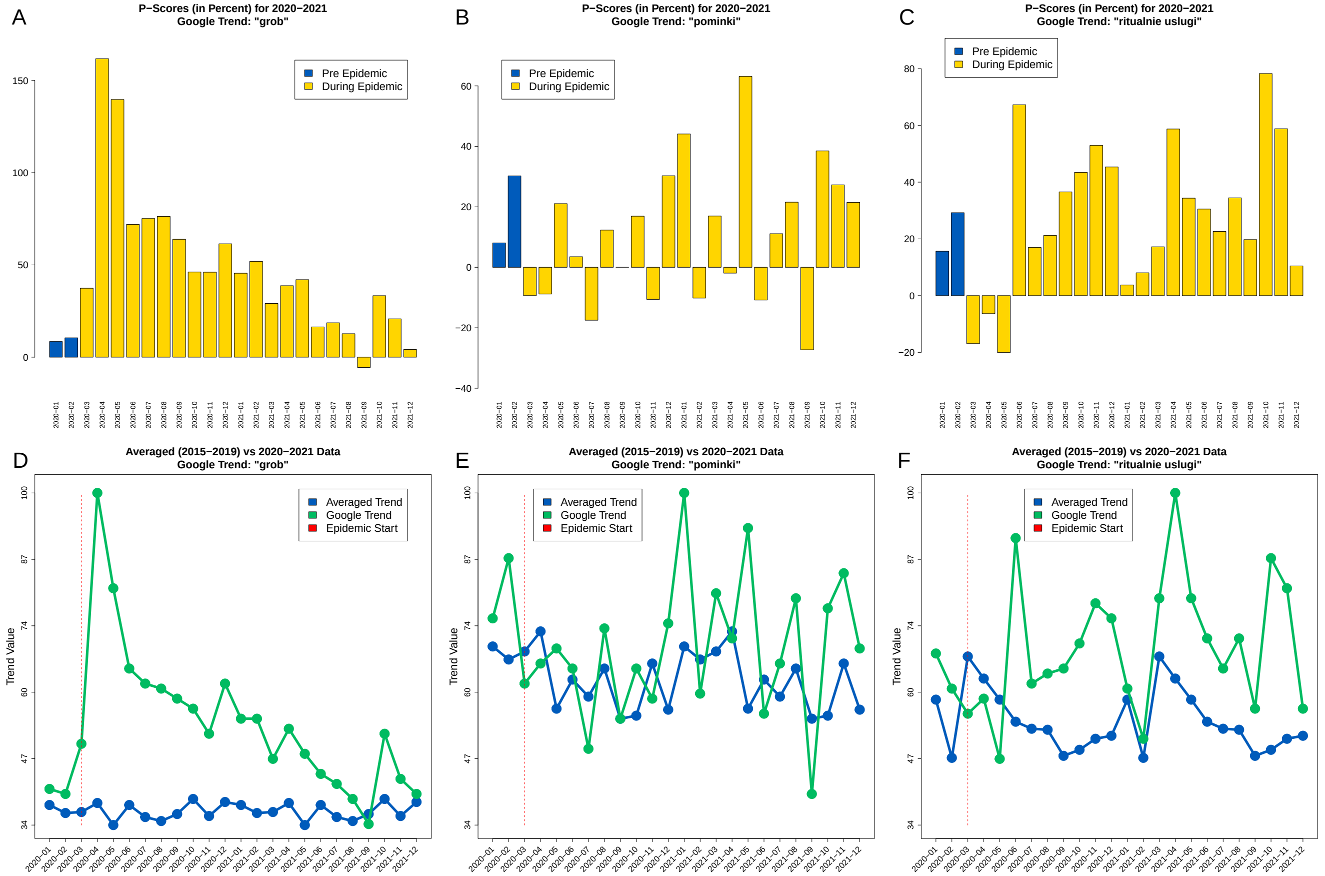

Figure S4. The visual summaries of the Russian language nonparametric Google Trends searches *P*-scores are presented for "coffin" (panel A), "memorial service" (panel B) and "funeral services" queries (panel C), where yellow bars are used for the epidemic period. The corresponding averaged data based on *five* years (blue) are plotted together with the query data (green) for coffin (panel D), memorial service (panel E) and funeral services (panel F). The vertical red line indicates the epidemic start period.

All-Cause Mortality Parametric 𝒫-scores

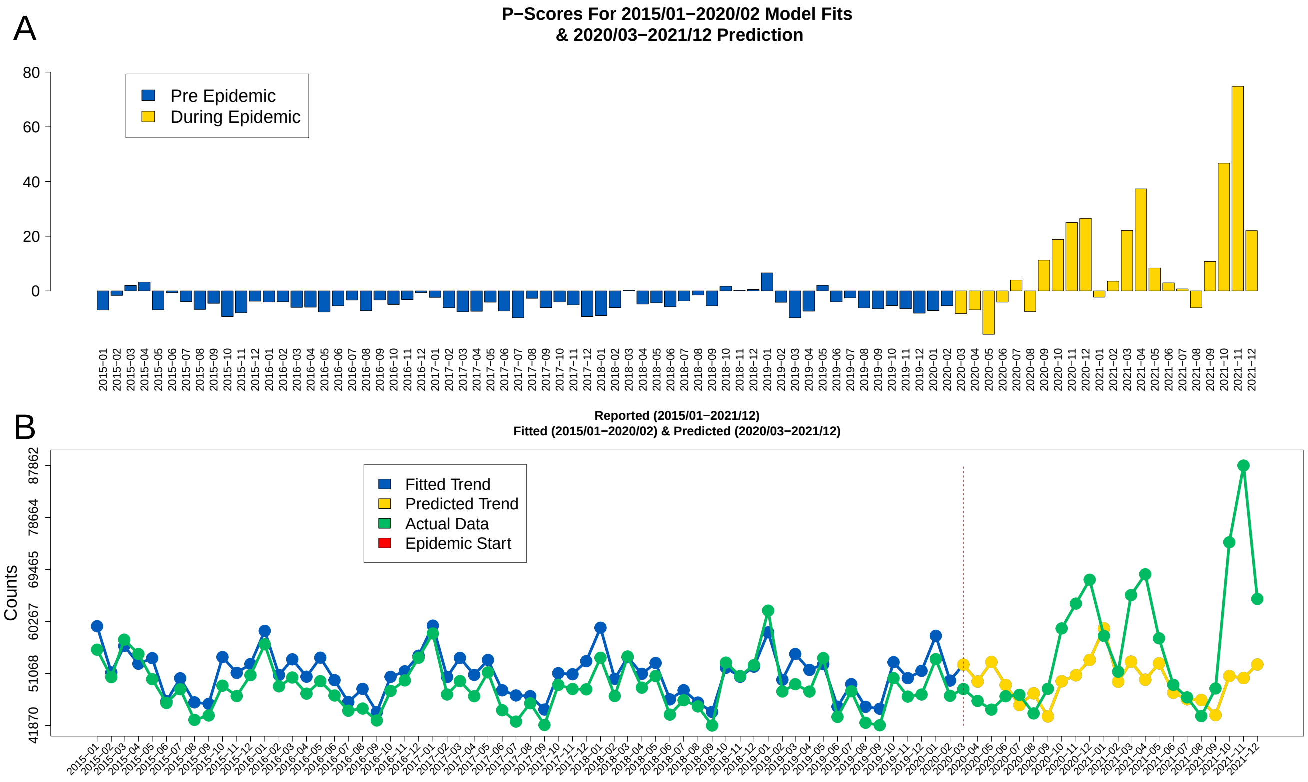

Figure S5. A) Parametric all-cause mortality 𝒫-scores based on the Prophet model *with* demographic characteristics (i.e. with 65+ covariate) where pre-pandemic scores are colored in blue and during pandemic scores are colored in yellow. B) Raw counts during 2015-2021 (green) together with the fitted (blue) and predicted during the pandemic (yellow) values by Prophet model *with* demographic characteristics. The vertical red line in panel B indicates the start of the pandemic.

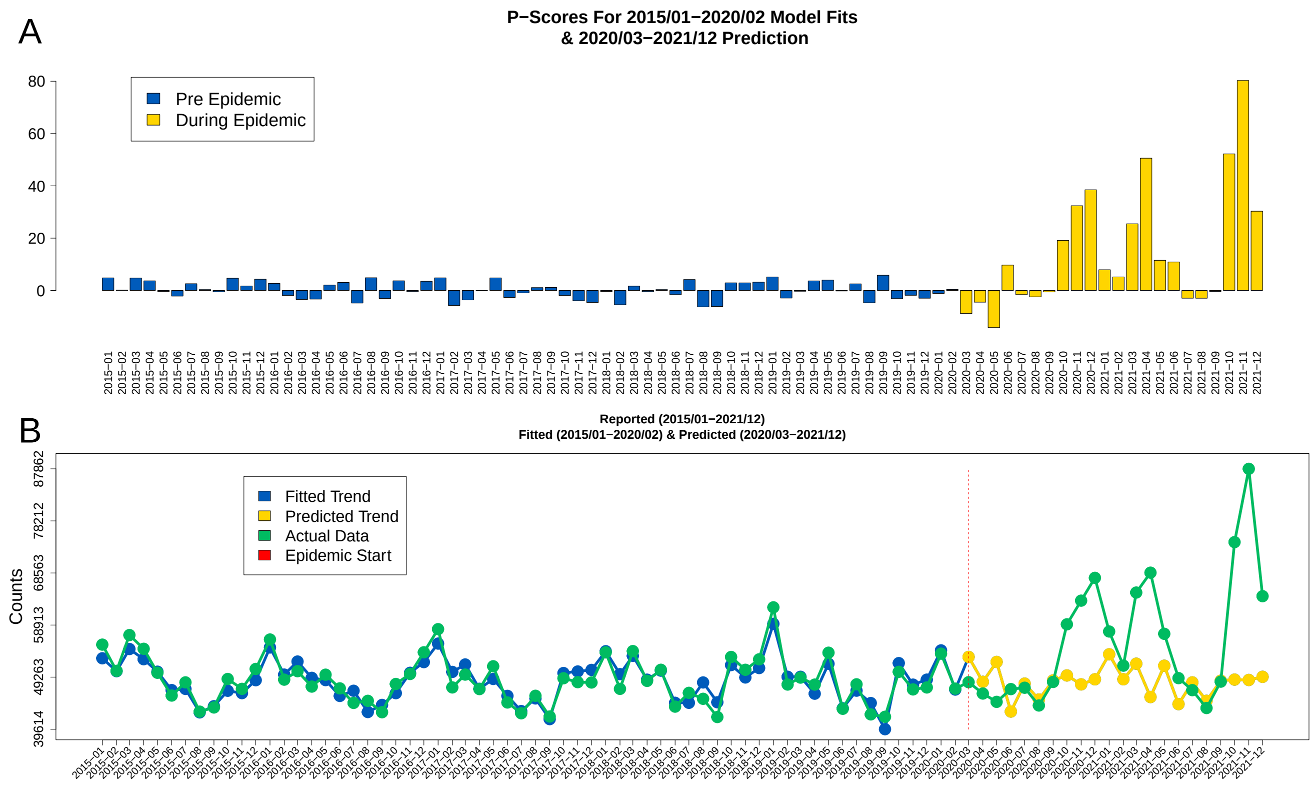

Figure S6. A) Parametric all-cause mortality $\mathscr{P}$-scores based on the ARIMA model *without* demographic characteristics where pre-pandemic scores are colored in blue and during pandemic scores are colored in yellow. B) Raw counts during 2015-2021 (green) together with the fitted (blue) and predicted during the pandemic (yellow) values by ARIMA model *without* demographic characteristics. The vertical red line in panel B indicates the start of the pandemic.

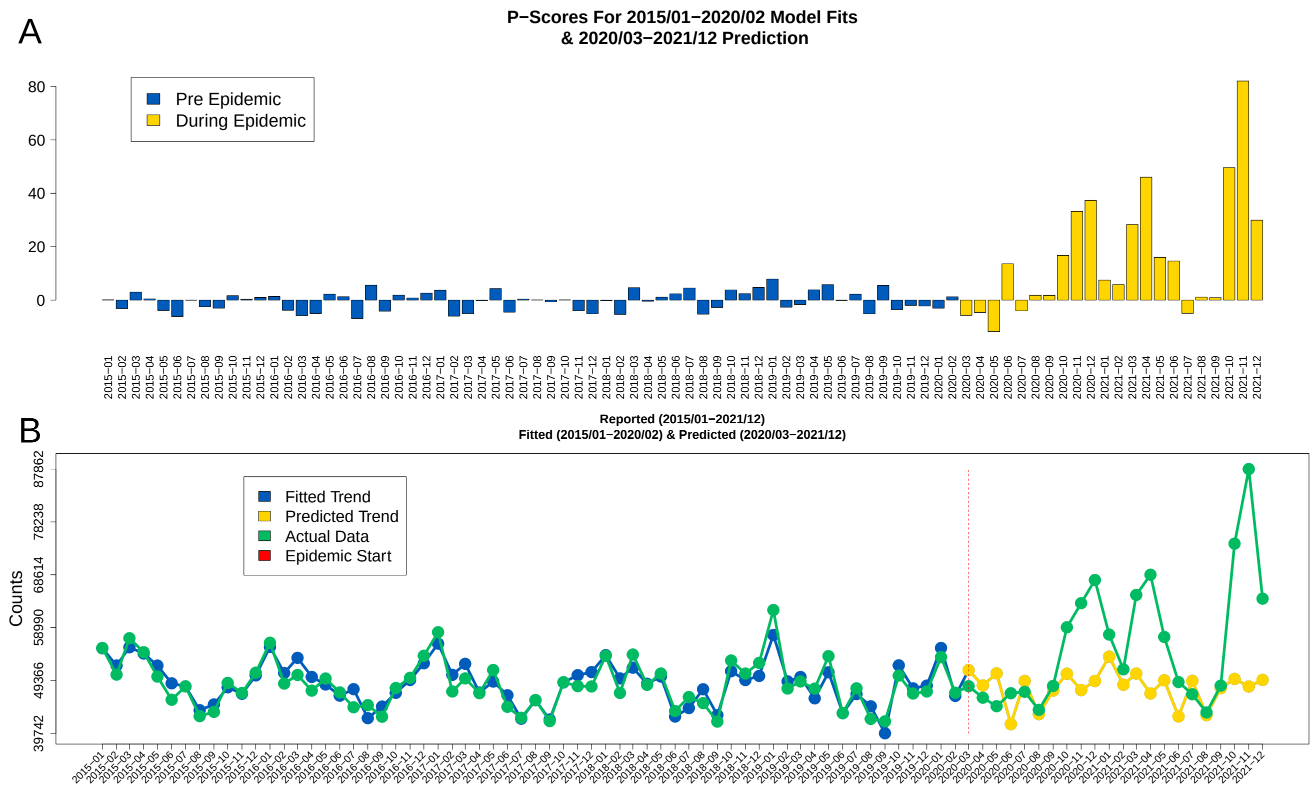

Figure S7. A) Parametric all-cause mortality $\mathscr{P}$-scores based on the ARIMA model *with* demographic characteristics (i.e. with 65+ covariate) where pre-pandemic scores are colored in blue and during pandemic scores are colored in yellow. B) Raw counts during 2015-2021 (green) together with the fitted (blue) and predicted during the pandemic (yellow) values by ARIMA model *with* demographic characteristics. The vertical red line in panel B indicates the start of the pandemic.

Comparisons of Models With and Without Demographic Characteristics

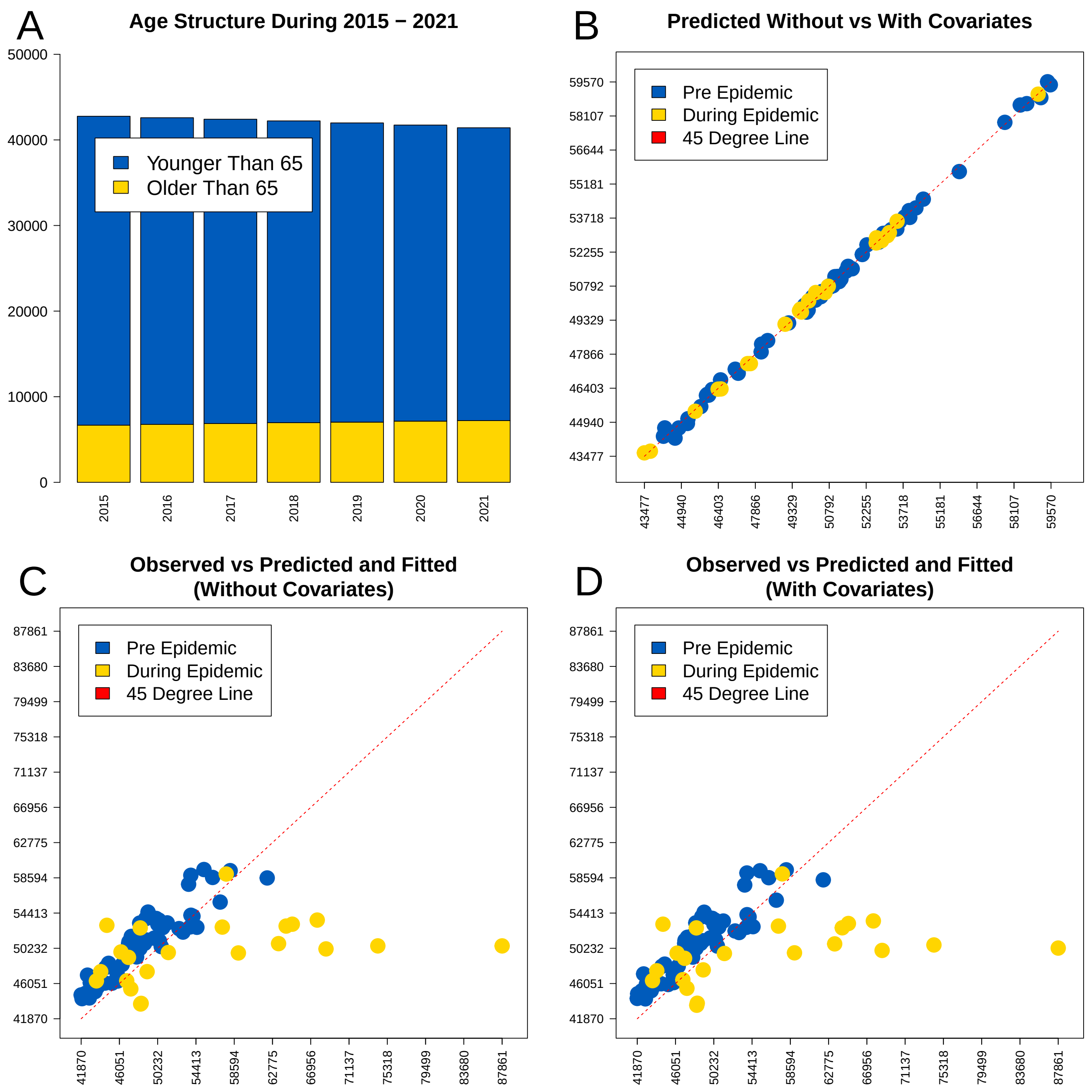

Figure S8. The visual summaries of the Prophet model fits compared without and with a 65+ covariate for five year histories: A) The total population and the proportion of individuals of age 65+. B) Comparison of predictions for two models: with vs without 65+ covariate. Summaries of observed vs predicted for model without (C) and with (D) covariate.

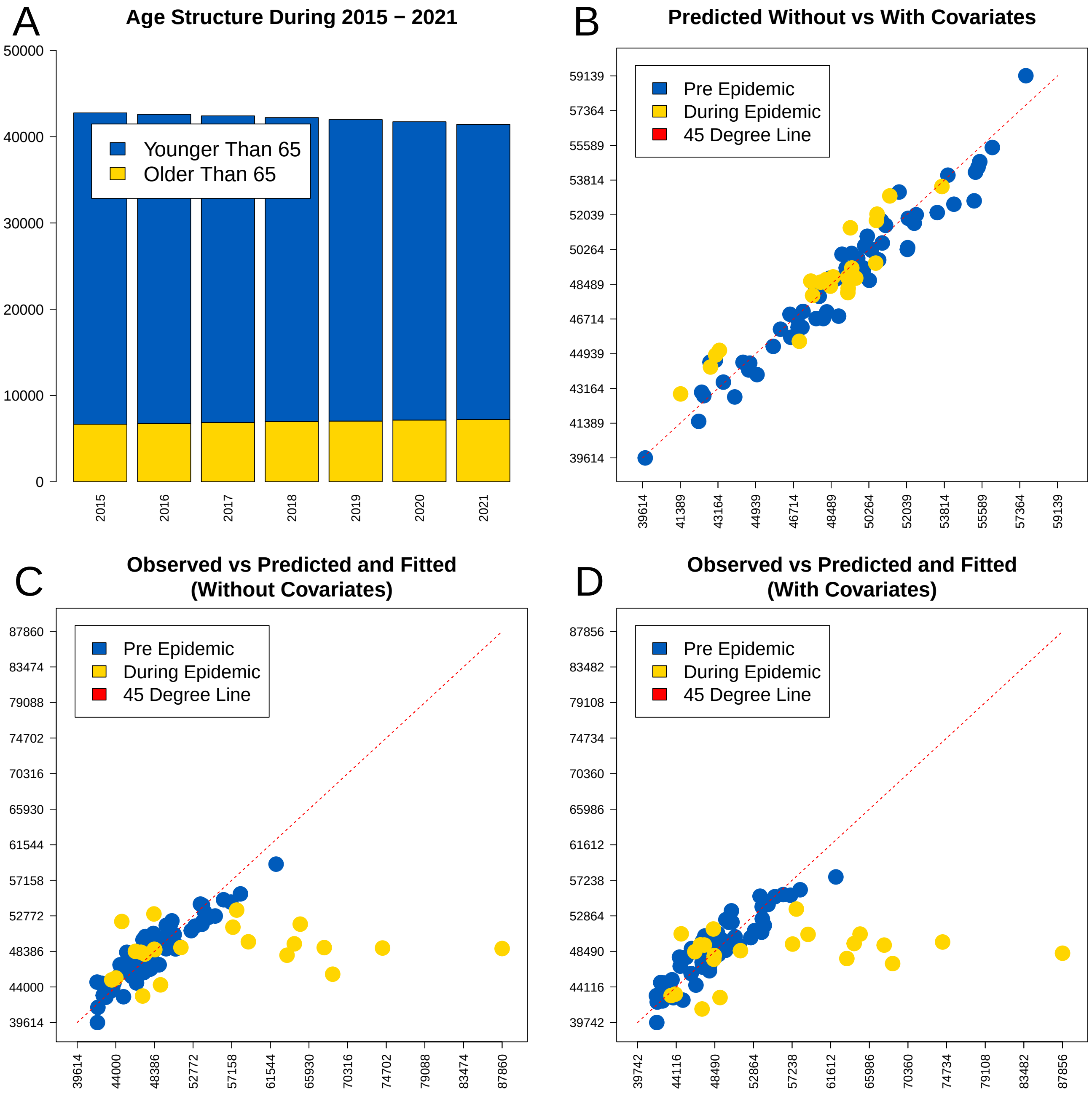

Figure S9. The visual summaries of the ARIMA model fits compared without and with a 65+ covariate for five year histories: A) The total population and the proportion of individuals of age 65+. B) Comparison of predictions for two models: with vs without 65+ covariate. Summaries of observed vs predicted for model without (C) and with (D) covariate.

Google Trends Parametric $\mathscr{P}$-Scores

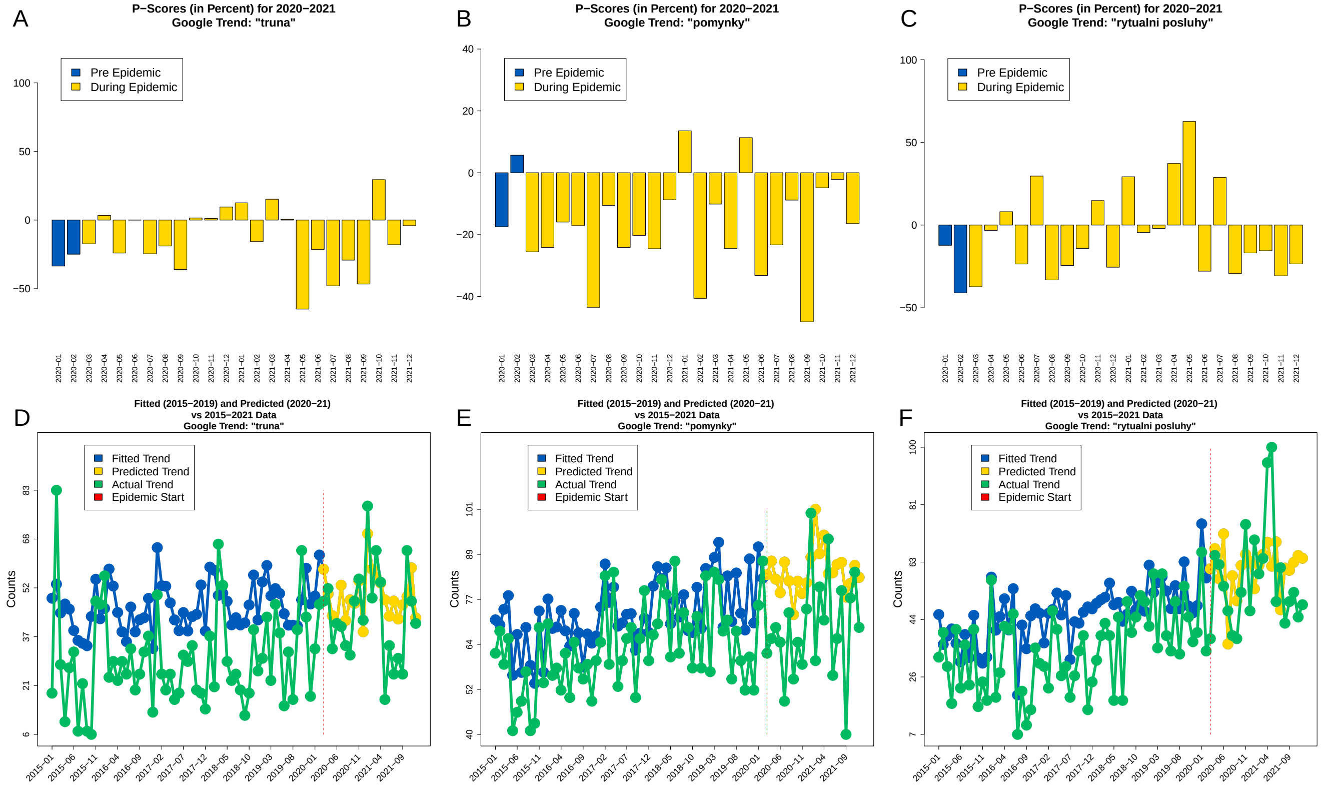

Figure S10. The visual summaries of the Ukrainian language parametric Google Trends searches $\mathscr{P}$-scores are presented for coffin (i.e. “truna”in panel A), memorial service (i.e. “pomynky” in panel B) and funeral services (i.e. “rytualni posluhy” in panel C) where yellow bars are values for the period of the CoV-SARS-2 epidemic. The corresponding model predictions based on 2015-2020 data (blue) are plotted together with the search data (green) for coffin (panel D), memorial service (panel E) and funeral services (panel F). The vertical red line indicates the date when first cases were reported.

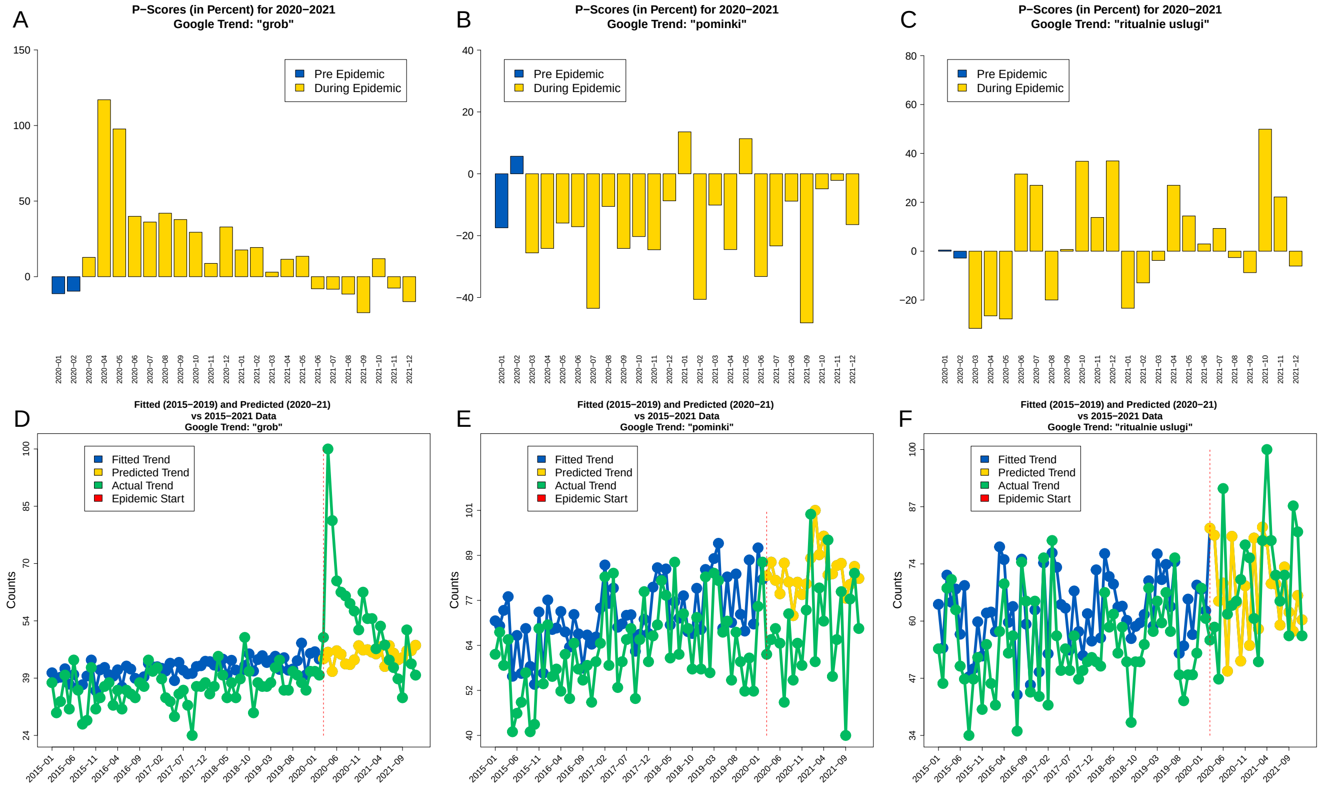

Figure S11. The visual summaries of the Russian language parametric Google Trends searches $\mathscr{P}$-scores are presented for coffin (i.e. “grob”in panel A), memorial service (i.e. “pominki” in panel B) and funeral services (i.e. “ritualnie uslugi” in panel C) where yellow bars are values for the period of the CoV-SARS-2 epidemic. The corresponding model predictions based on 2015-2020 data (blue) are plotted together with the search data (green) for coffin (panel D), memorial service (panel E) and funeral services (panel F). The vertical red line indicates the date when first cases were reported.

Google Trends and Mortality Relationship

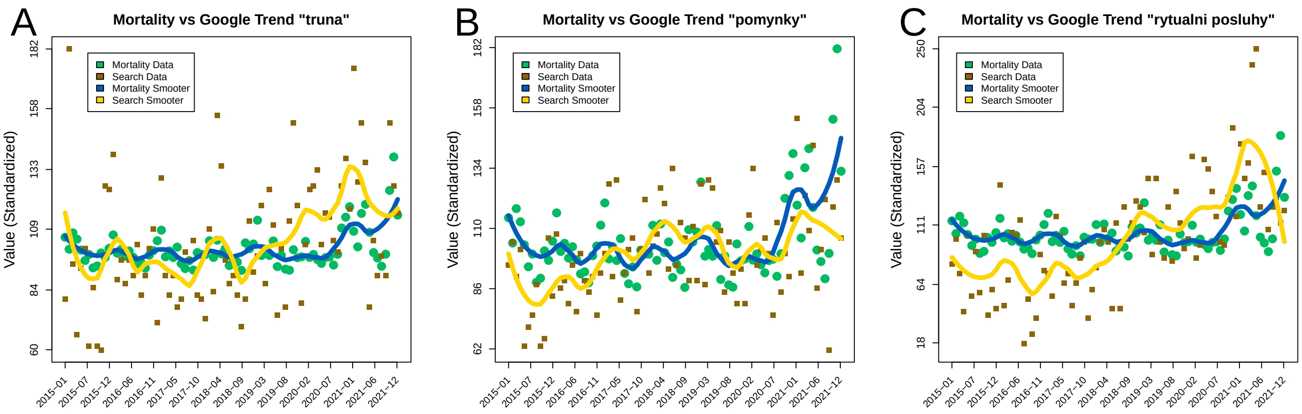

Figure S12. The visual summaries of the joint plotting of the standardized mortality counts versus the Ukrainian language monthly standardized Google trends data together with the corresponding smothers from January 2015 until December 2021 for queries: “truna” meaning coffin (panel A), "pomynky" meaning memorial service (panel B) and “rytualni posluhy” meaning funeral services (panel C).

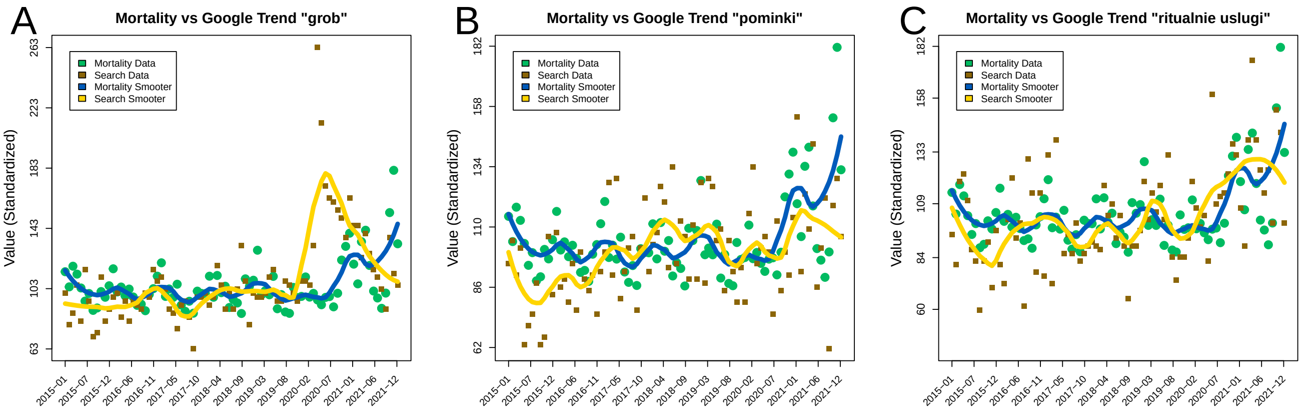

Figure S13. The visual summaries of the joint plotting of the standardized mortality counts versus the the Russian language monthly standardized Google trends data together with the corresponding smothers from January 2015 until December 2021 for queries: “grob” meaning coffin (panel A), "pominki" meaning memorial service (panel B) and “ritualnie uslugi” meaning funeral services (panel C). The vertical red line demonstrates the start of the epidemic, based on the first confirmed case date.

Name	p_{GR}	$p_{GR(Sm)}$
“truna”	0.07	0.20
“pomynky”	0.00	0.89
“rytualni posluhy”	0.03	0.00

Table S1. The p-values of the Granger causality test for the ability of the Ukrainian language monthly standardized Google trends data to forecast the standardized incidence from January 2015 until December 2021. The p-values for the standardized series are presented in the first column (p_{GR}) and the corresponding p-values for the standardized and smoothed versions of the series are presented in the second column ($p_{GR(Sm)}$).

Name	p_{GR}	$p_{GR(Sm)}$
“grob”	0.80	0.00
“pominki”	0.00	0.89
“ritualnie uslugi”	0.16	0.42

Table S2. The p-values of the Granger causality test for the ability of the Russian language monthly standardized Google trends data to forecast the standardized incidence from January 2015 until December 2021. The p-values for the standardized series are presented in the first column (p_{GR}) and the corresponding p-values for the standardized and smoothed versions of the series are presented in the second column ($p_{GR(Sm)}$).

Mortality Causes Analysis

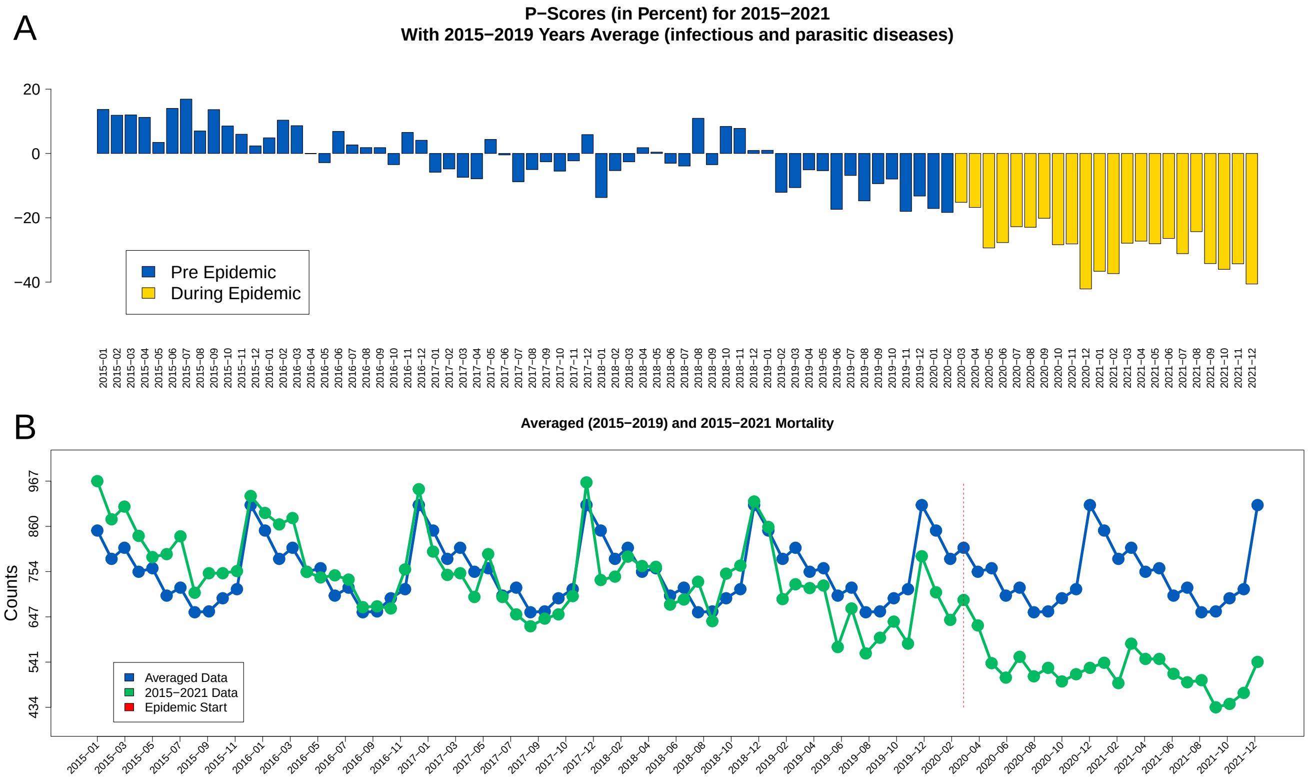

Figure S14. The visual summaries of nonparametric "infectious and parasitic disease" mortality *P*-scores presented at panel A. Yellow bars represent the epidemic period. The corresponding averaged data (blue) based on the previous five (panel B) years are presented along the reported data for 2020 – 2021 (yellow). The vertical red line shows the date on which when epidemic has started.

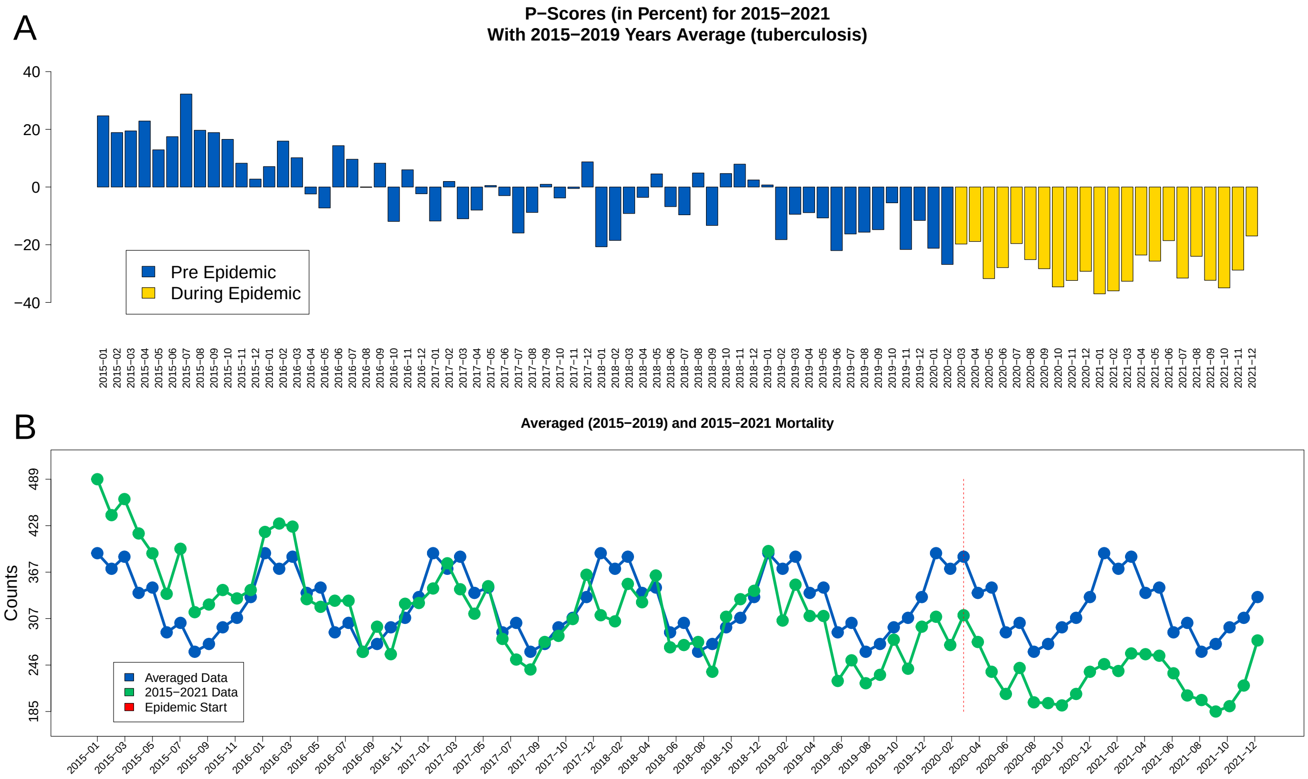

Figure S15. The visual summaries of nonparametric "tuberculosis" mortality *P*-scores presented at panel A. Yellow bars represent the epidemic period. The corresponding averaged data (blue) based on the previous five (panel B) years are presented along the reported data for 2020 – 2021 (yellow). The vertical red line shows the date on which when epidemic has started.

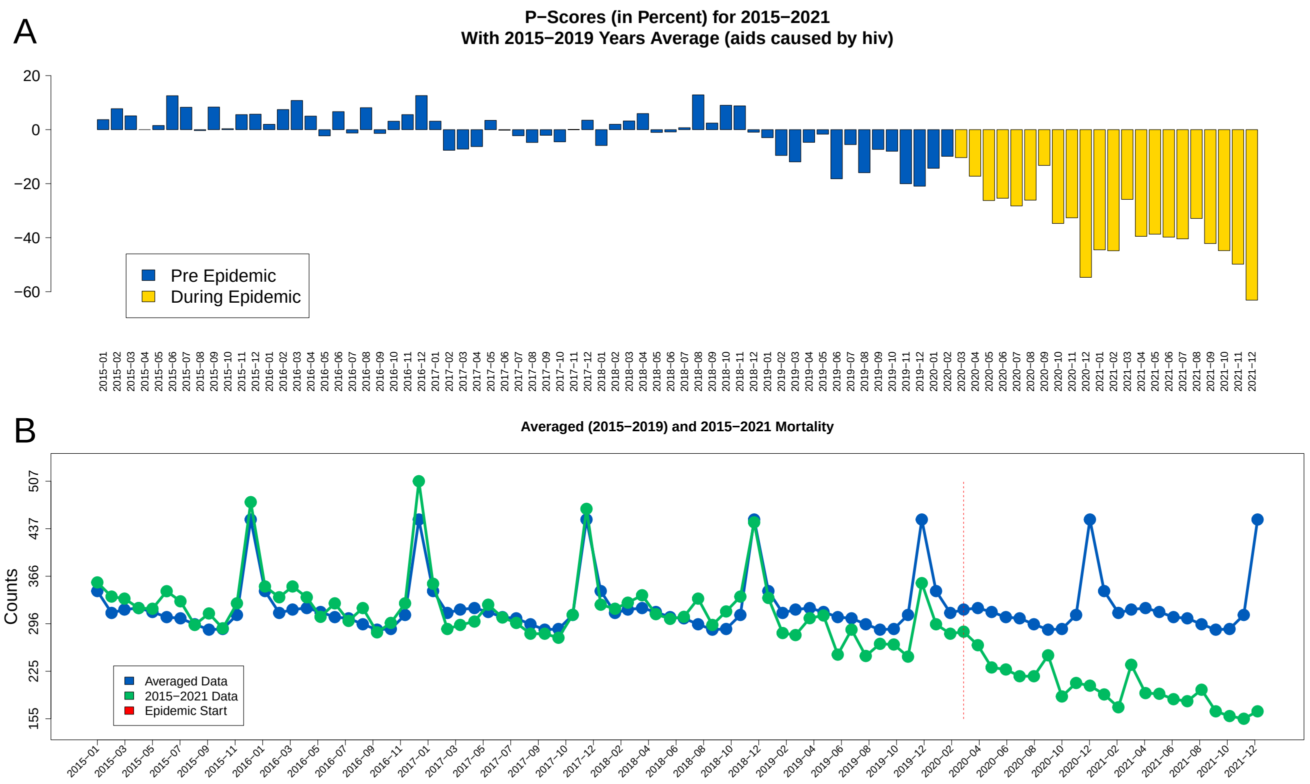

Figure S16. The visual summaries of nonparametric "AIDS cause by HIV" mortality *P*-scores presented at panel A. Yellow bars represent the epidemic period. The corresponding averaged data (blue) based on the previous five (panel B) years are presented along the reported data for 2020 – 2021 (yellow). The vertical red line shows the date on which when epidemic has started.

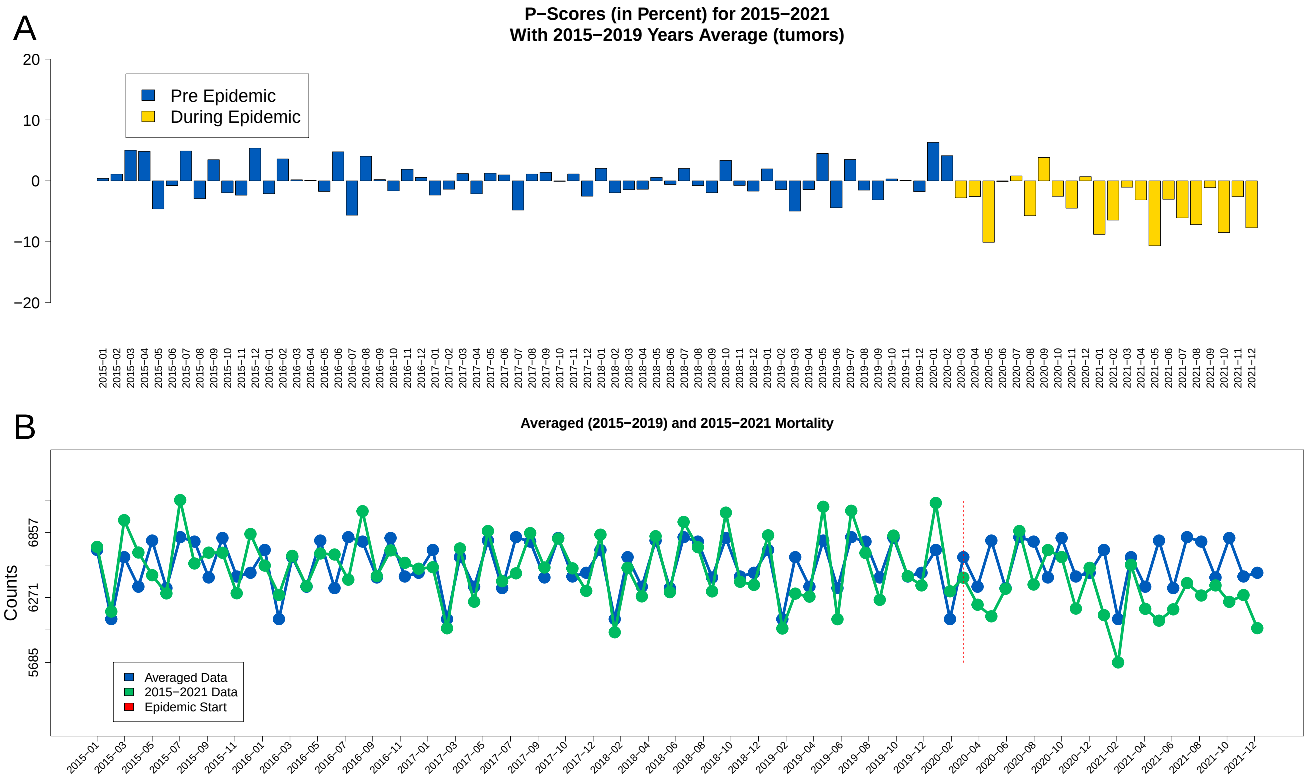

Figure S17. The visual summaries of nonparametric "tumors" mortality *P*-scores presented at panel A. Yellow bars represent the epidemic period. The corresponding averaged data (blue) based on the previous five (panel B) years are presented along the reported data for 2020 – 2021 (yellow). The vertical red line shows the date on which when epidemic has started.

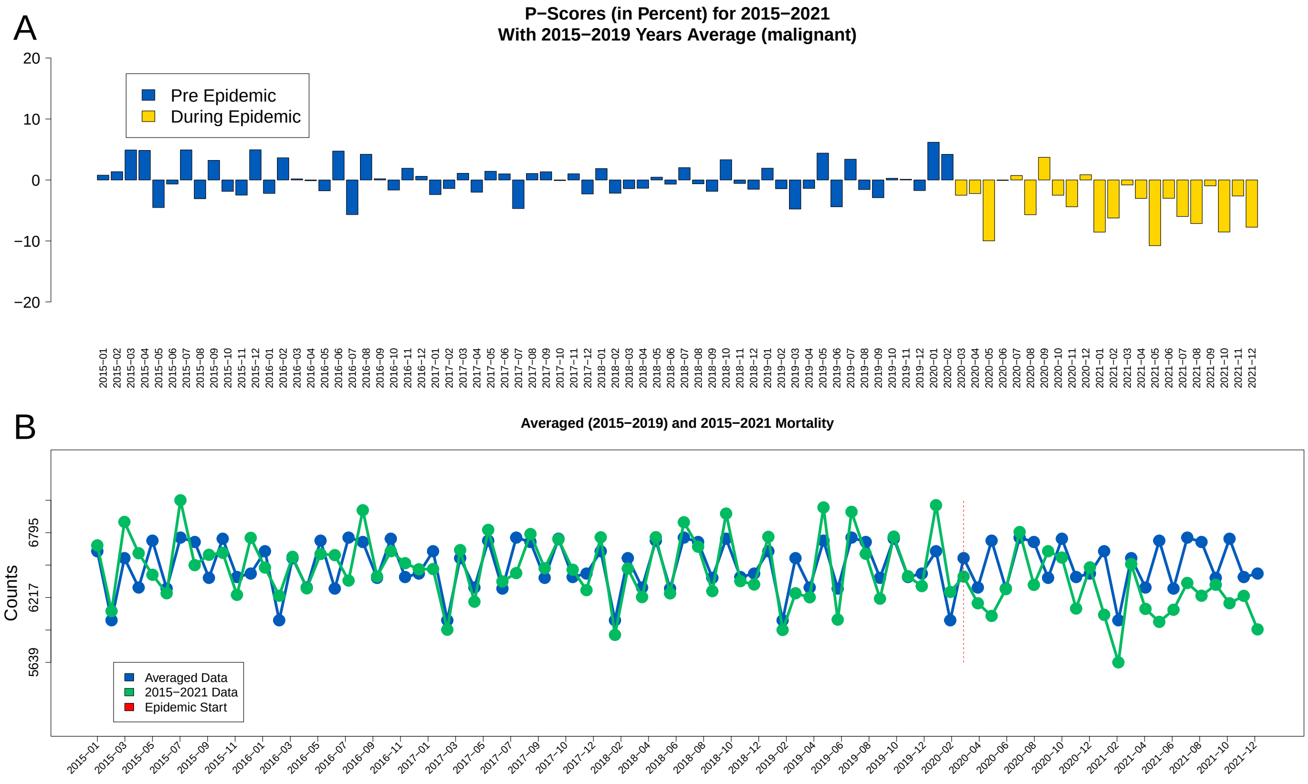

Figure S18. The visual summaries of nonparametric "malignant tumors" mortality *P*-scores presented at panel A. Yellow bars represent the epidemic period. The corresponding averaged data (blue) based on the previous five (panel B) years are presented along the reported data for 2020 – 2021 (yellow). The vertical red line shows the date on which when epidemic has started.

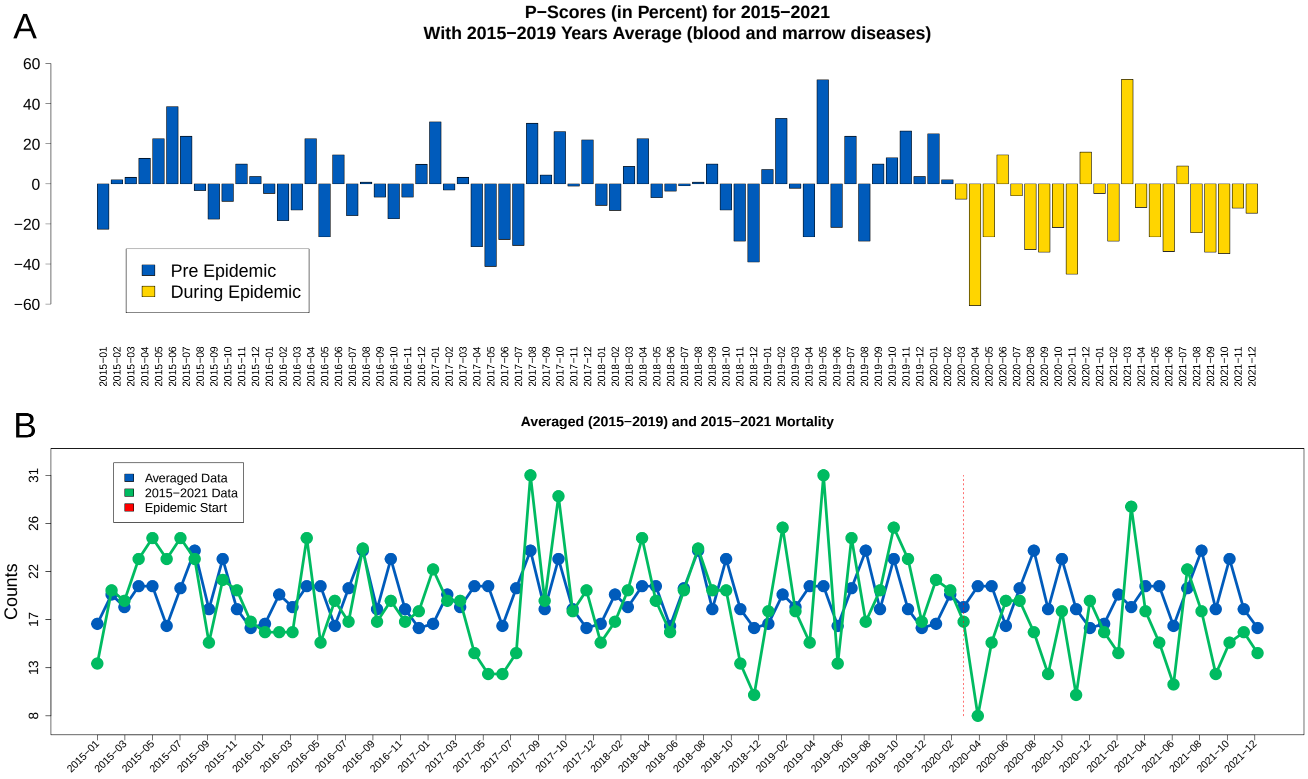

Figure S19. The visual summaries of nonparametric "blood and marrow diseases" mortality *P*-scores presented at panel A. Yellow bars represent the epidemic period. The corresponding averaged data (blue) based on the previous five (panel B) years are presented along the reported data for 2020 – 2021 (yellow). The vertical red line shows the date on which when epidemic has started.

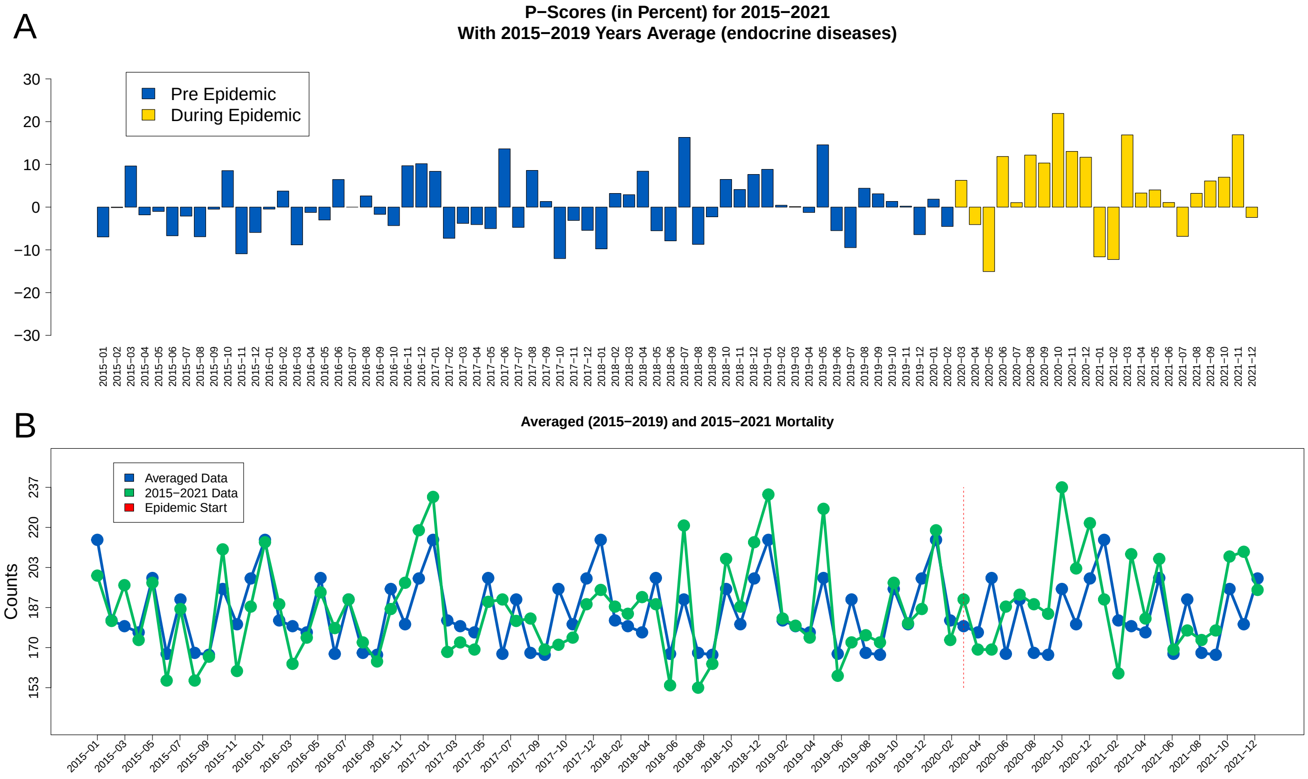

Figure S20. The visual summaries of nonparametric "endocrine diseases" mortality *P*-scores presented at panel A. Yellow bars represent the epidemic period. The corresponding averaged data (blue) based on the previous five (panel B) years are presented along the reported data for 2020 – 2021 (yellow). The vertical red line shows the date on which when epidemic has started.

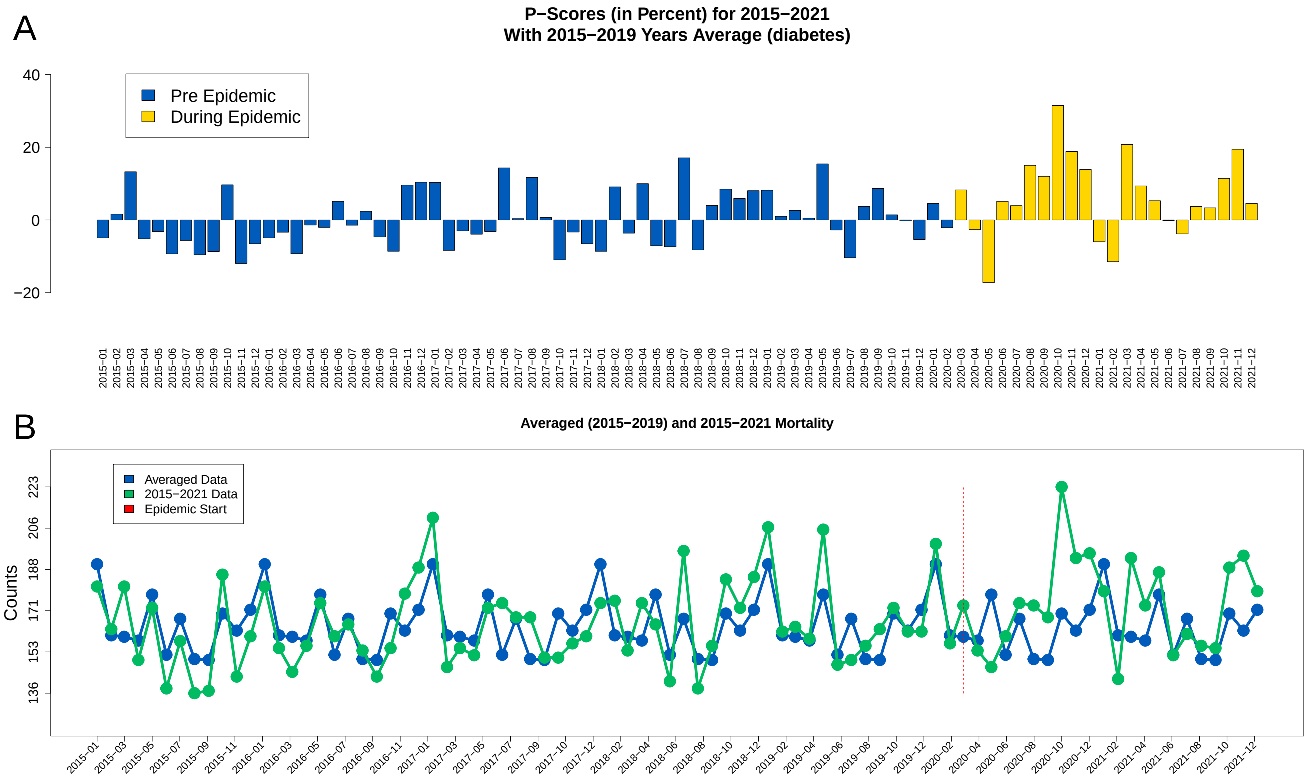

Figure S21. The visual summaries of nonparametric "diabetes" mortality *P*-scores presented at panel A. Yellow bars represent the epidemic period. The corresponding averaged data (blue) based on the previous five (panel B) years are presented along the reported data for 2020 – 2021 (yellow). The vertical red line shows the date on which when epidemic has started.

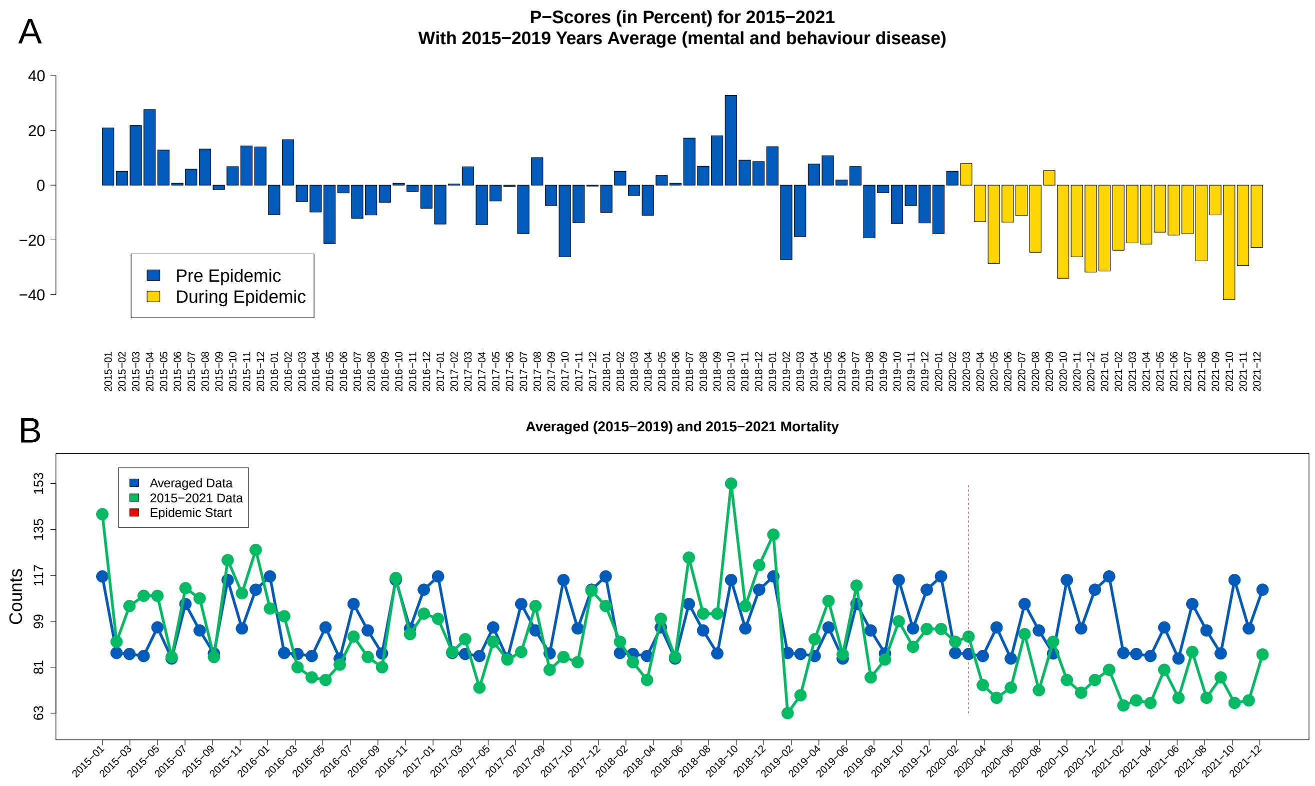

Figure S22. The visual summaries of nonparametric "mental and behavior disorders" mortality *P*-scores presented at panel A. Yellow bars represent the epidemic period. The corresponding averaged data (blue) based on the previous five (panel B) years are presented along the reported data for 2020 – 2021 (yellow). The vertical red line shows the date on which when epidemic has started.

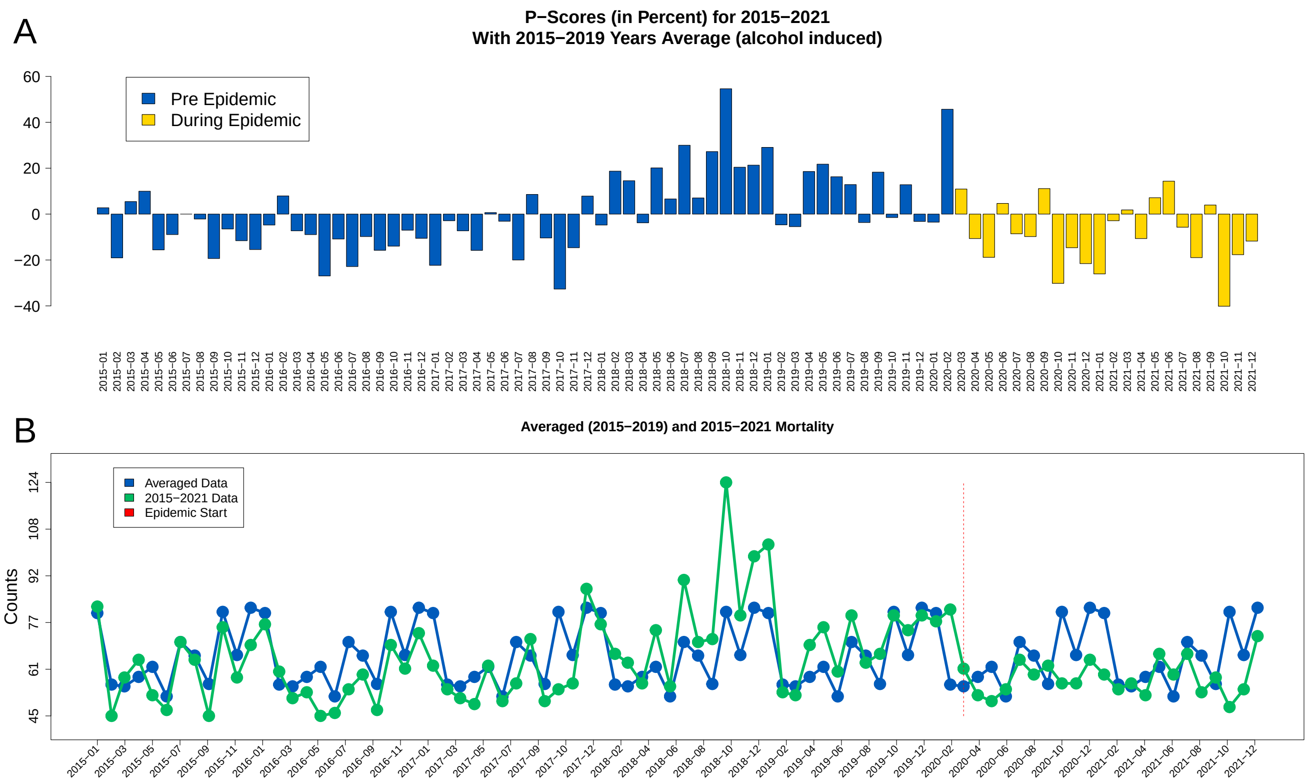

Figure S23. The visual summaries of nonparametric "alcohol induced mental and behavior disorders" mortality *P*-scores presented at panel A. Yellow bars represent the epidemic period. The corresponding averaged data (blue) based on the previous five (panel B) years are presented along the reported data for 2020 – 2021 (yellow). The vertical red line shows the date on which when epidemic has started.

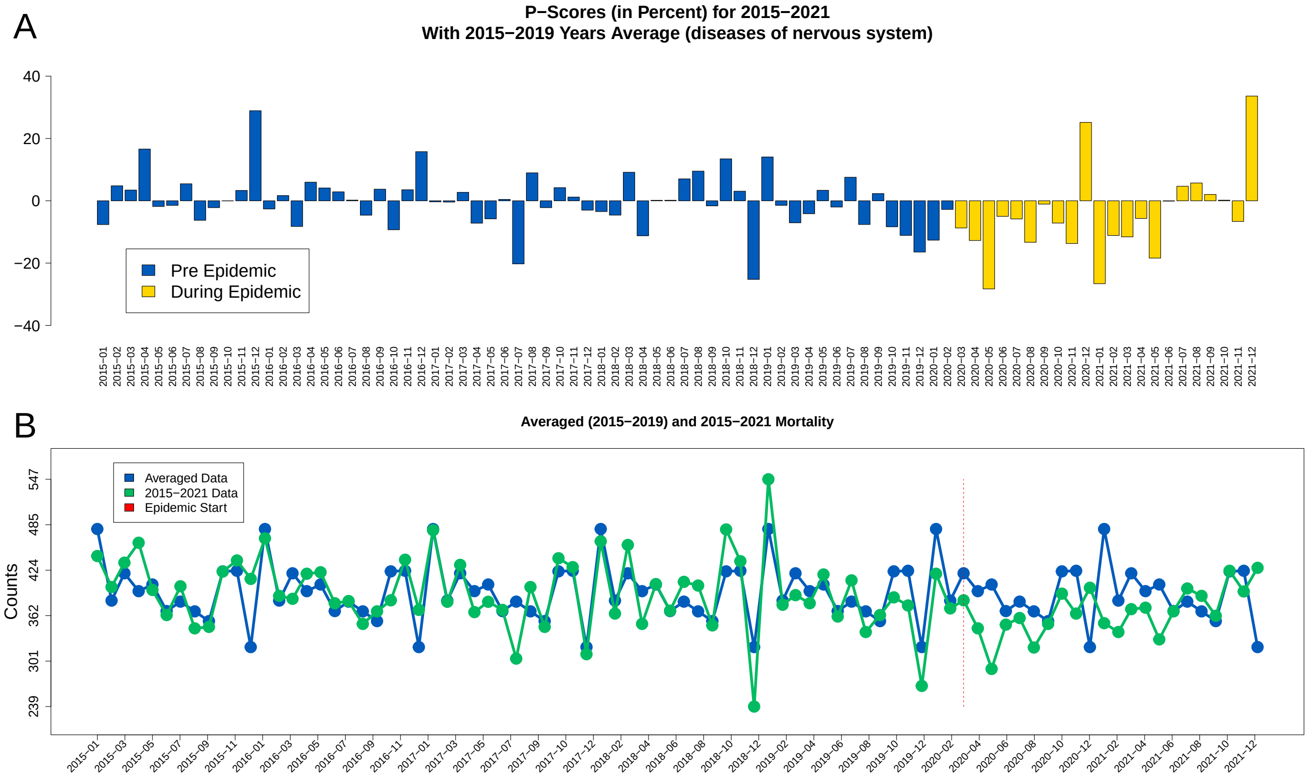

Figure S24. The visual summaries of nonparametric "diseases of the nervous system" mortality P-scores presented at panel A. Yellow bars represent the epidemic period. The corresponding averaged data (blue) based on the previous five (panel B) years are presented along the reported data for 2020 – 2021 (yellow). The vertical red line shows the date on which when epidemic has started.

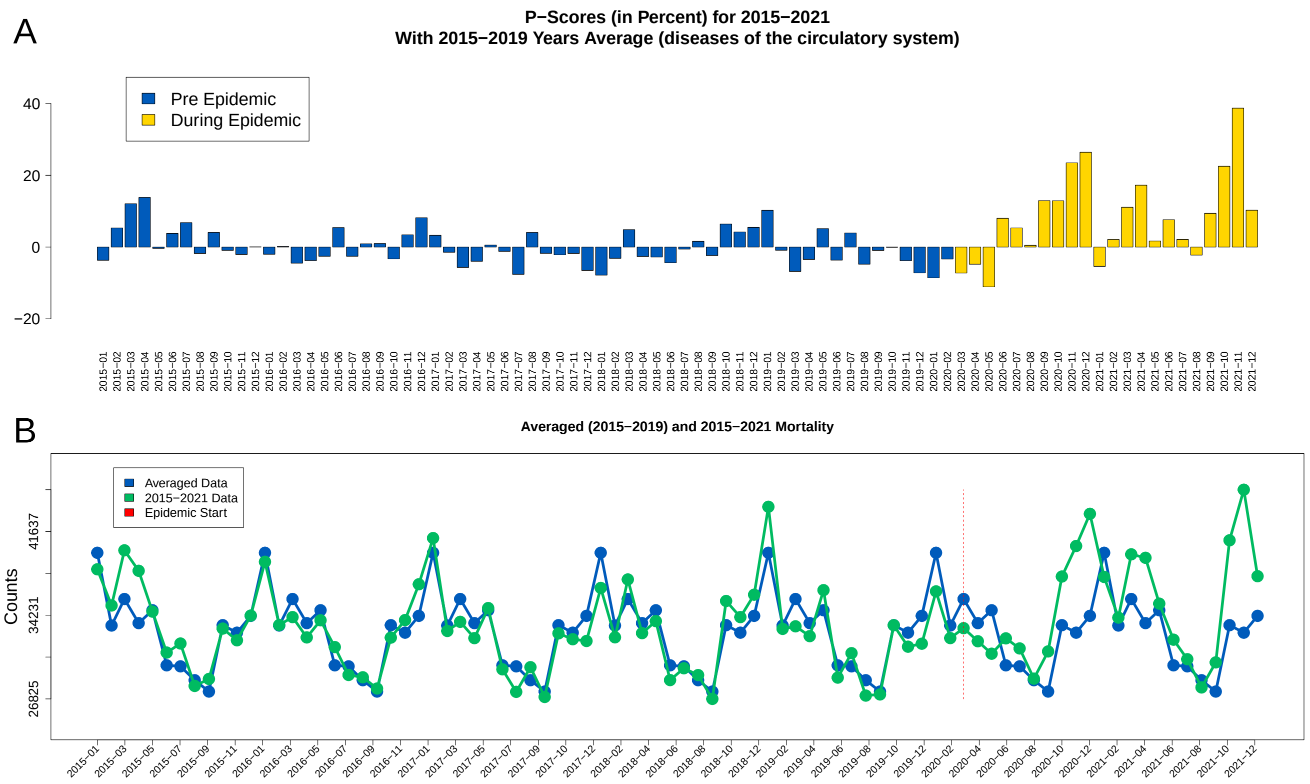

Figure S25. The visual summaries of nonparametric "diseases of the circulatory system" mortality *P*-scores presented at panel A. Yellow bars represent the epidemic period. The corresponding averaged data (blue) based on the previous five (panel B) years are presented along the reported data for 2020 – 2021 (yellow). The vertical red line shows the date on which when epidemic has started.

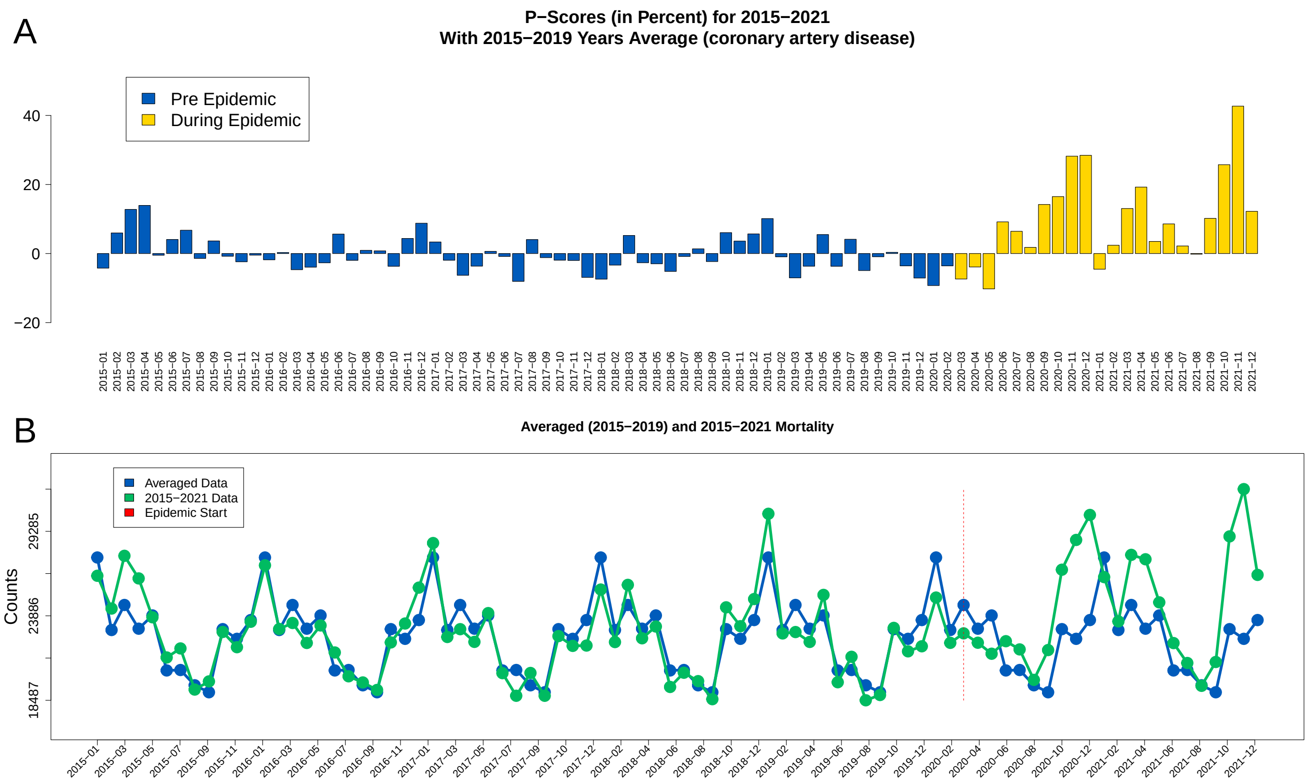

Figure S26. The visual summaries of nonparametric "coronary artery disease" mortality *P*-scores presented at panel A. Yellow bars represent the epidemic period. The corresponding averaged data (blue) based on the previous five (panel B) years are presented along the reported data for 2020 – 2021 (yellow). The vertical red line shows the date on which when epidemic has started.

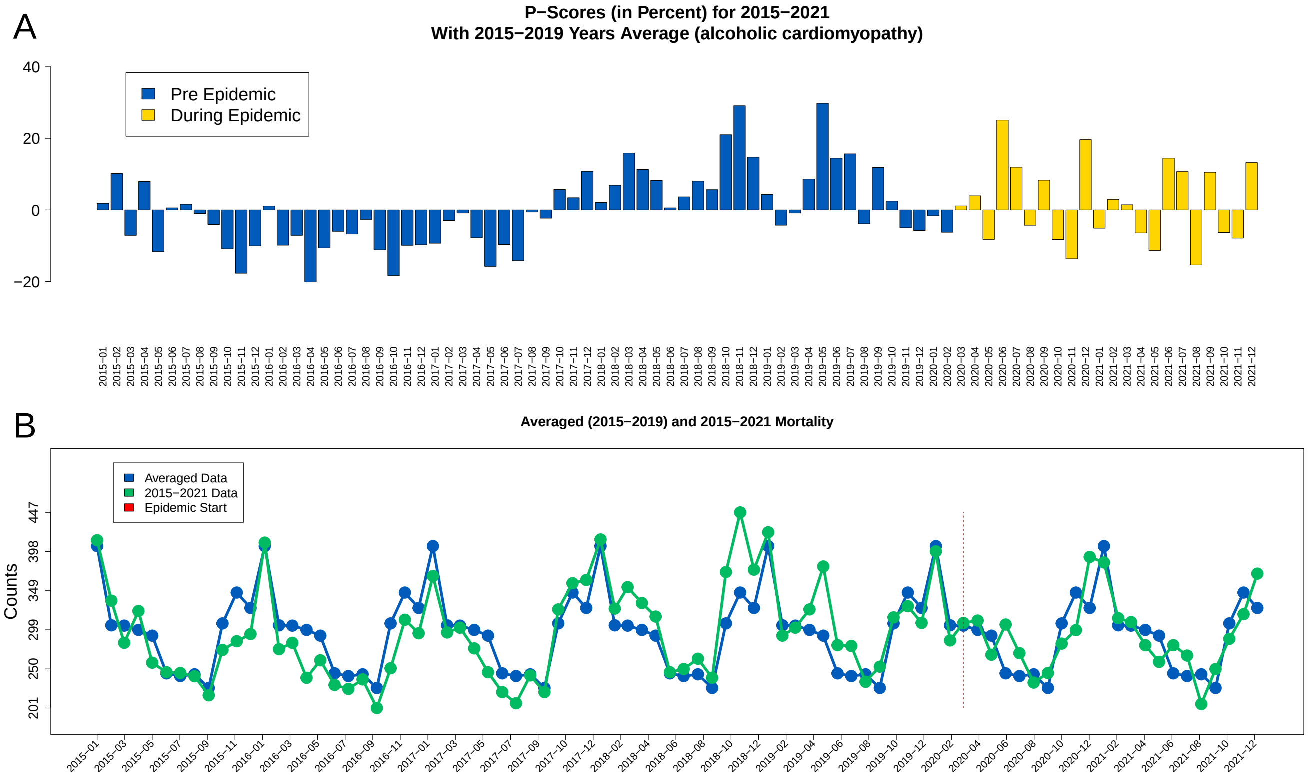

Figure S27. The visual summaries of nonparametric "alcoholic cardiomyopathy" mortality *P*-scores presented at panel A. Yellow bars represent the epidemic period. The corresponding averaged data (blue) based on the previous five (panel B) years are presented along the reported data for 2020 – 2021 (yellow). The vertical red line shows the date on which when epidemic has started.

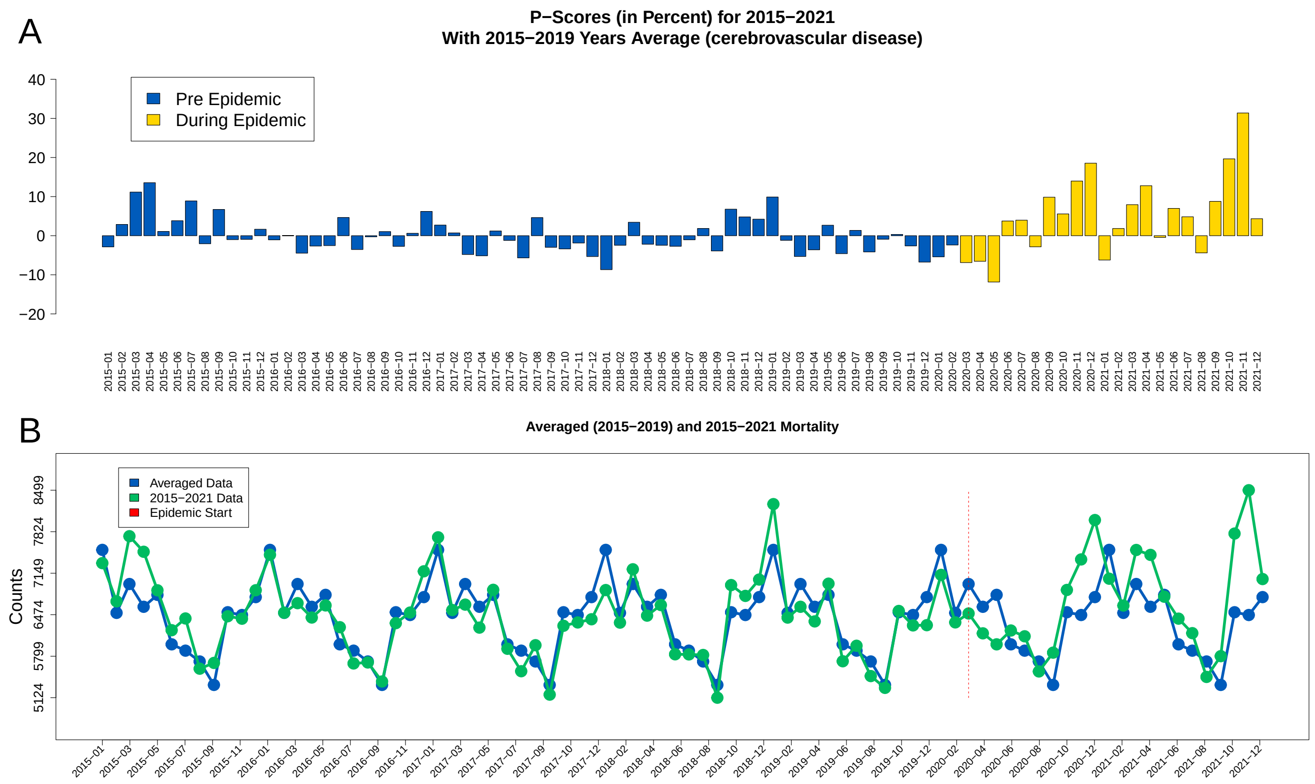

Figure S28. The visual summaries of nonparametric "cerebrovascular disease" mortality *P*-scores presented at panel A. Yellow bars represent the epidemic period. The corresponding averaged data (blue) based on the previous five (panel B) years are presented along the reported data for 2020 – 2021 (yellow). The vertical red line shows the date on which when epidemic has started.

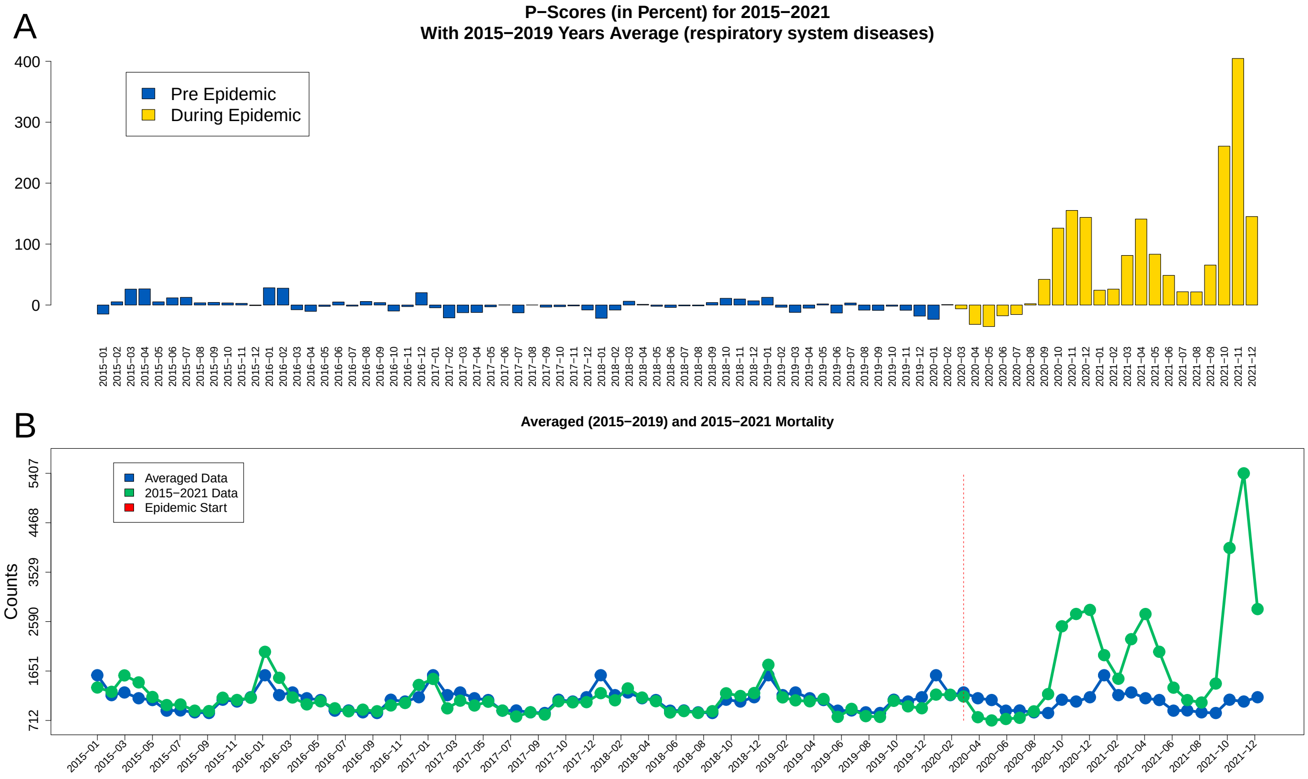

Figure S29. The visual summaries of nonparametric "respiratory system diseases" mortality *P*-scores presented at panel A. Yellow bars represent the epidemic period. The corresponding averaged data (blue) based on the previous five (panel B) years are presented along the reported data for 2020 – 2021 (yellow). The vertical red line shows the date on which when epidemic has started.

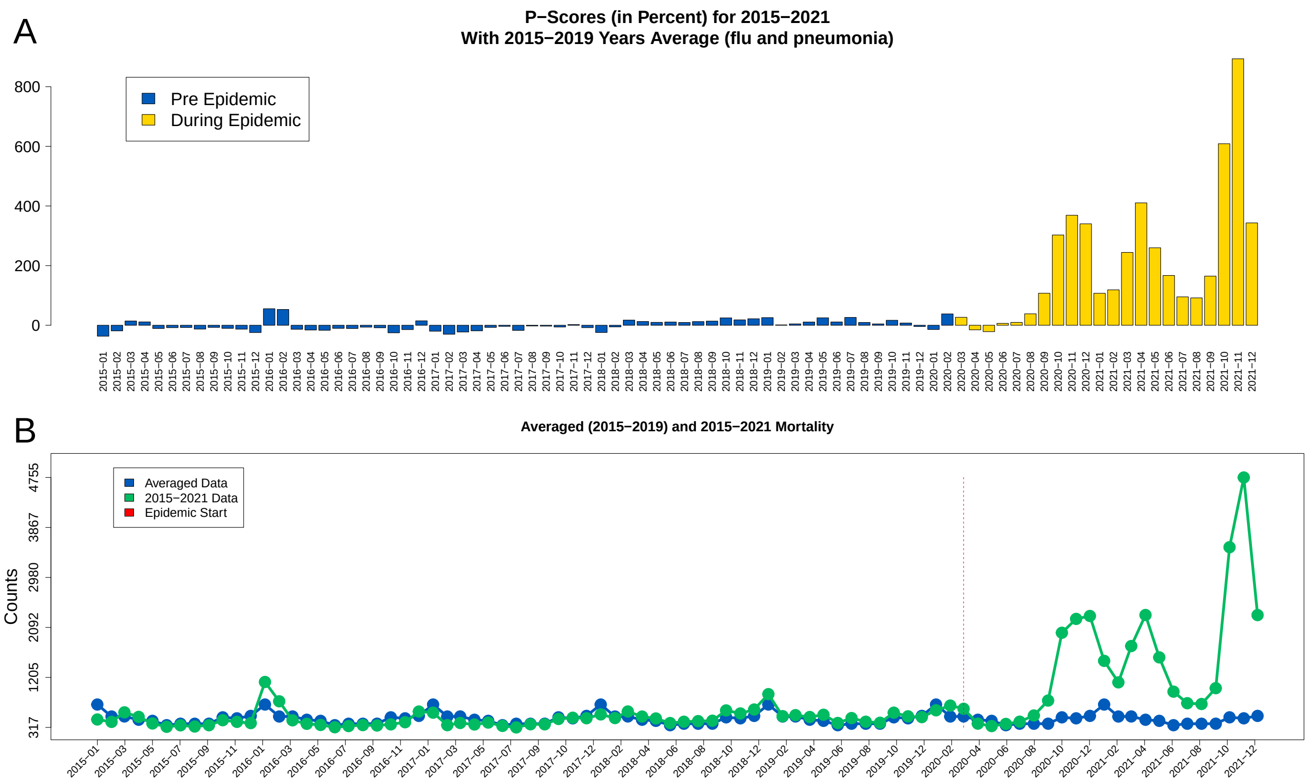

Figure S30. The visual summaries of nonparametric "flu and pneumonia" mortality *P*-scores presented at panel A. Yellow bars represent the epidemic period. The corresponding averaged data (blue) based on the previous five (panel B) years are presented along the reported data for 2020 – 2021 (yellow). The vertical red line shows the date on which when epidemic has started.

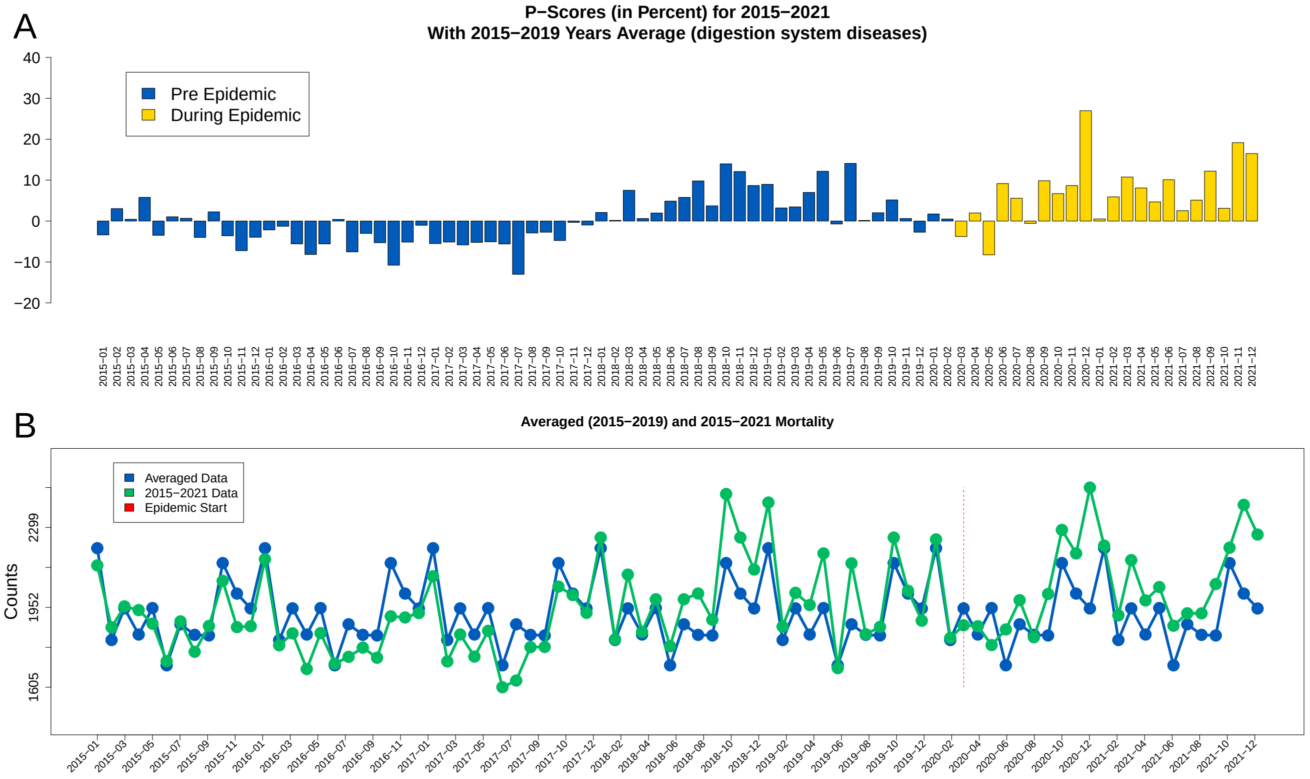

Figure S31. The visual summaries of nonparametric "digestion system diseases" mortality *P*-scores presented at panel A. Yellow bars represent the epidemic period. The corresponding averaged data (blue) based on the previous five (panel B) years are presented along the reported data for 2020 – 2021 (yellow). The vertical red line shows the date on which when epidemic has started.

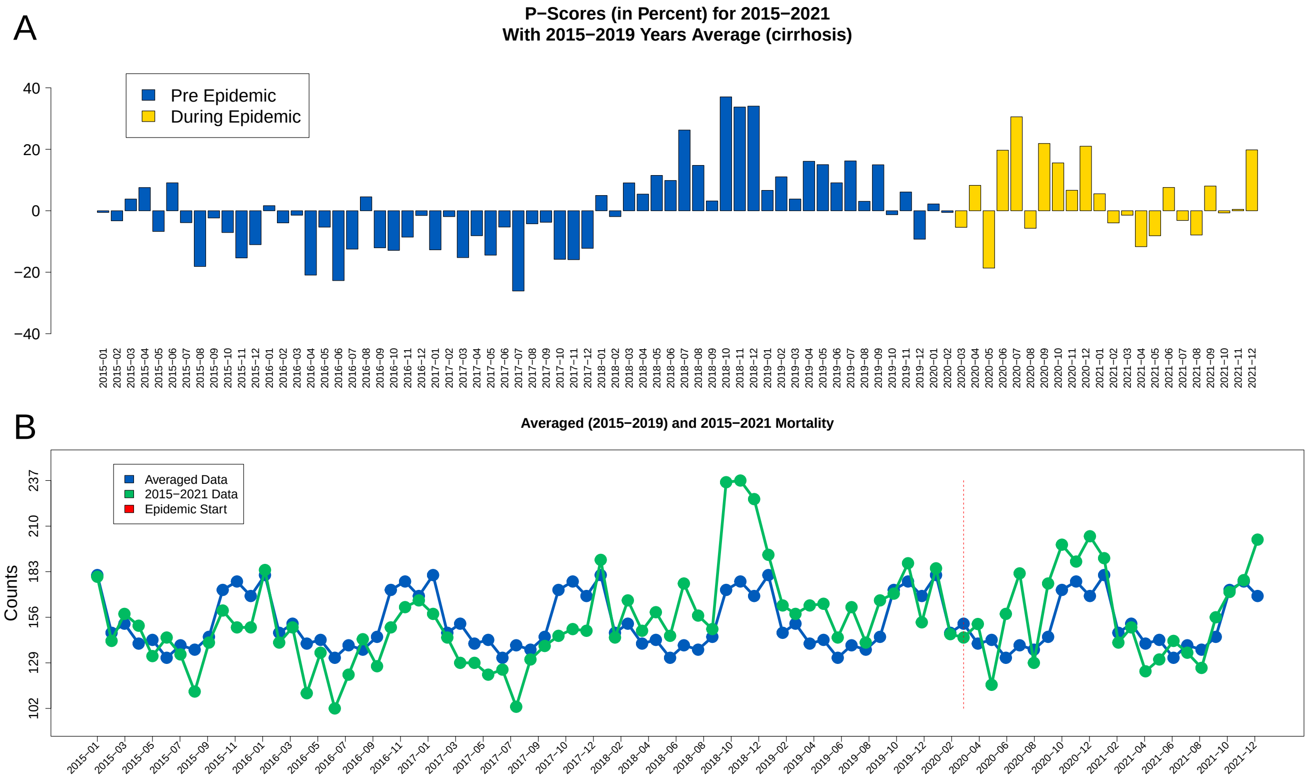

Figure S32. The visual summaries of nonparametric "cirrhosis" mortality *P*-scores presented at panel A. Yellow bars represent the epidemic period. The corresponding averaged data (blue) based on the previous five (panel B) years are presented along the reported data for 2020 – 2021 (yellow). The vertical red line shows the date on which when epidemic has started.

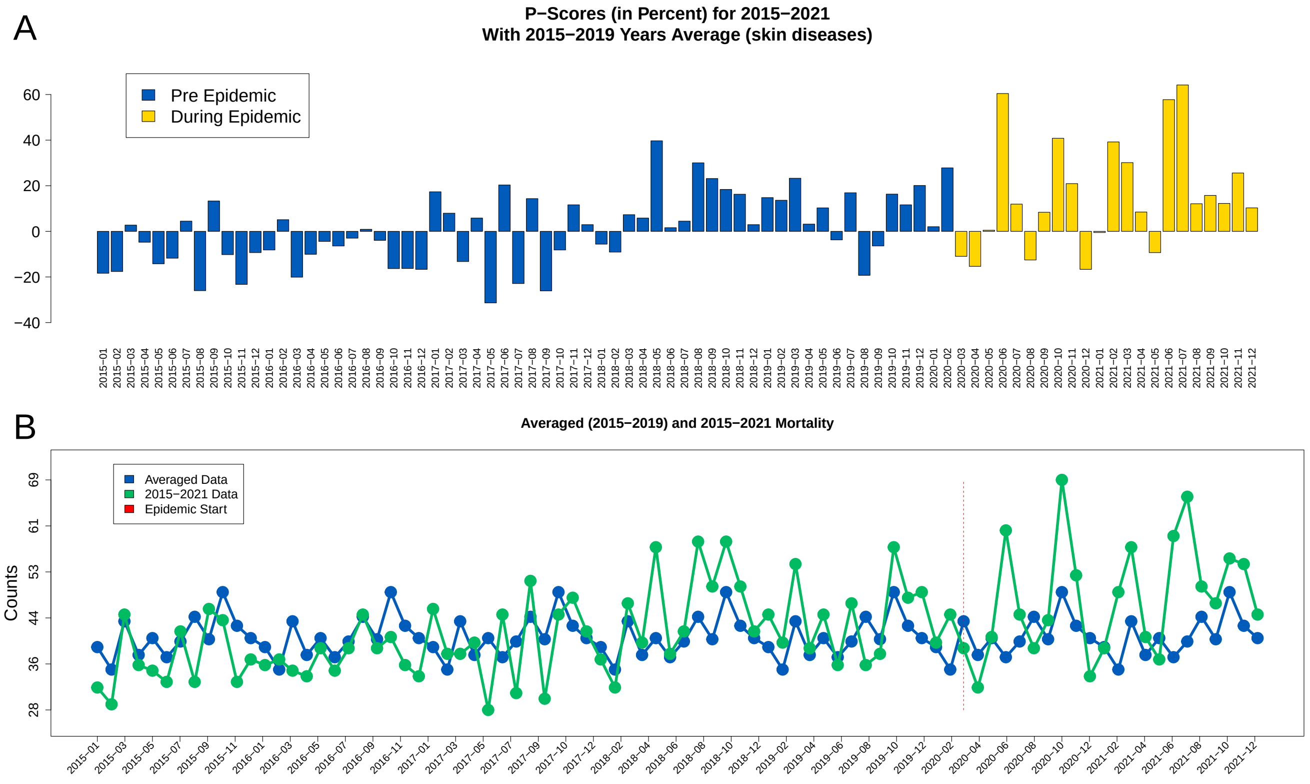

Figure S33. The visual summaries of nonparametric "skin diseases" mortality *P*-scores presented at panel A. Yellow bars represent the epidemic period. The corresponding averaged data (blue) based on the previous five (panel B) years are presented along the reported data for 2020 – 2021 (yellow). The vertical red line shows the date on which when epidemic has started.

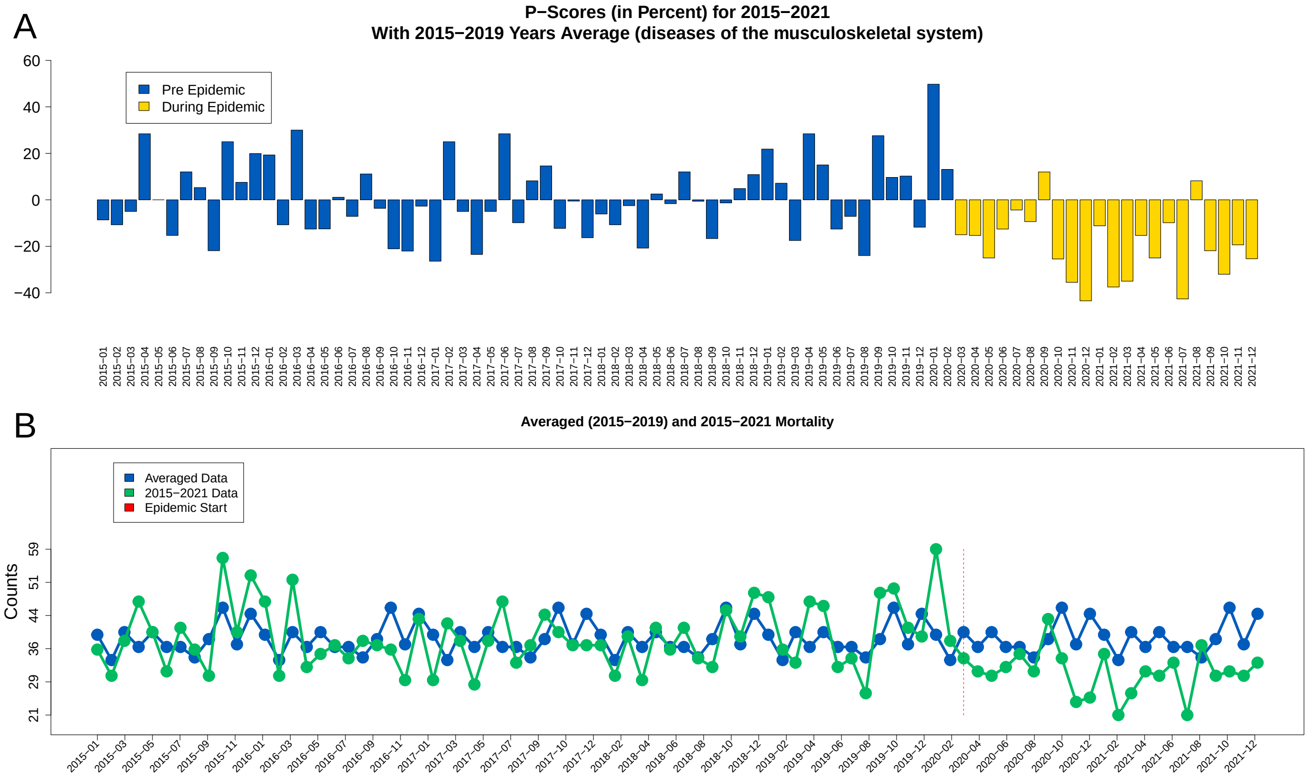

Figure S34. The visual summaries of nonparametric "diseases of the musculoskeletal system" mortality *P*-scores presented at panel A. Yellow bars represent the epidemic period. The corresponding averaged data (blue) based on the previous five (panel B) years are presented along the reported data for 2020 – 2021 (yellow). The vertical red line shows the date on which when epidemic has started.

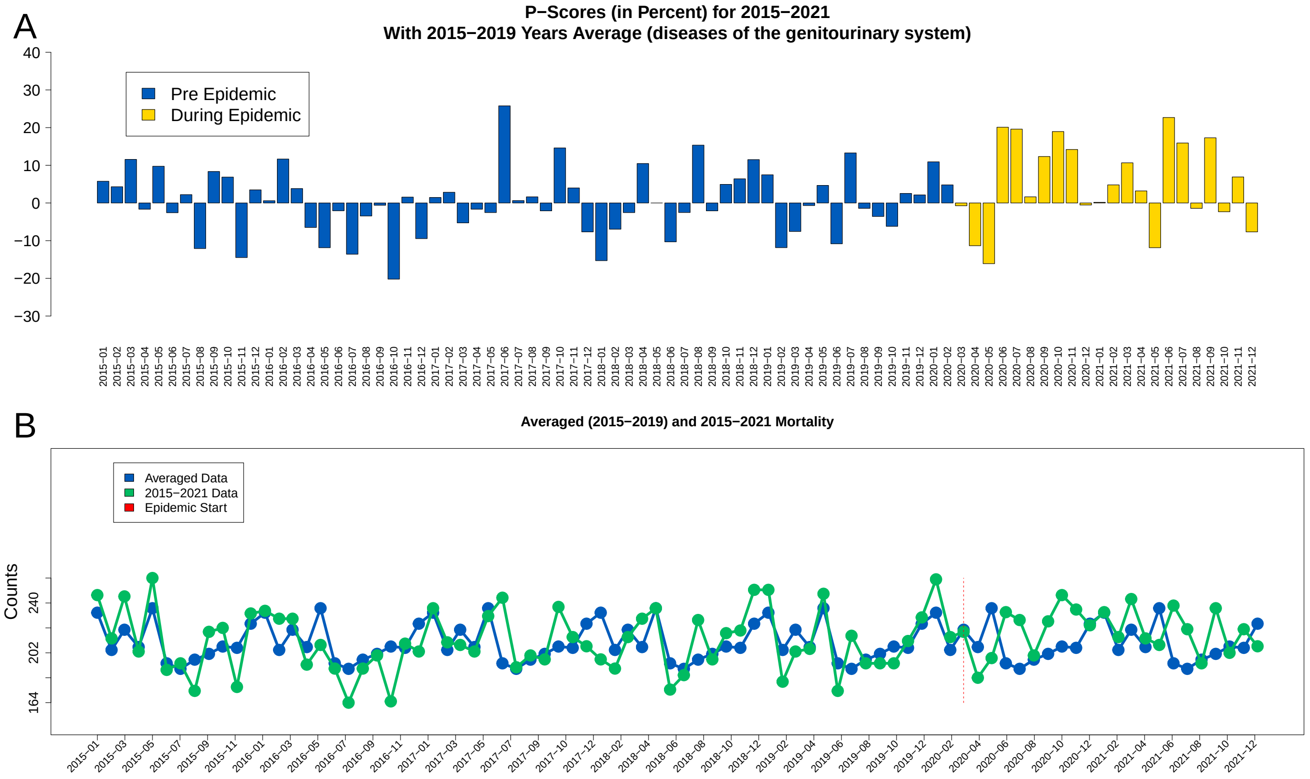

Figure S35. The visual summaries of nonparametric "diseases of the genitourinary system" mortality *P*-scores presented at panel A. Yellow bars represent the epidemic period. The corresponding averaged data (blue) based on the previous five (panel B) years are presented along the reported data for 2020 – 2021 (yellow). The vertical red line shows the date on which when epidemic has started.

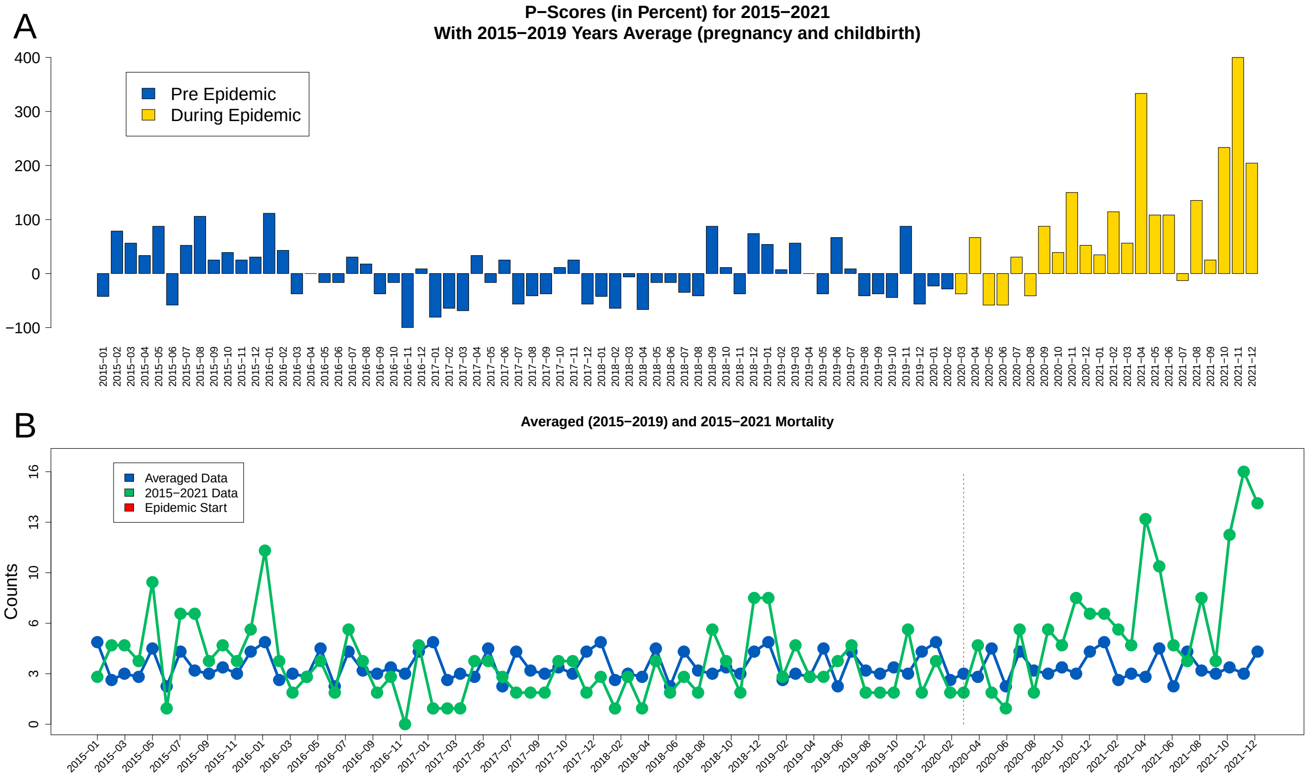

Figure S36. The visual summaries of nonparametric "pregnancy and childbirth" mortality *P*-scores presented at panel A. Yellow bars represent the epidemic period. The corresponding averaged data (blue) based on the previous five (panel B) years are presented along the reported data for 2020 – 2021 (yellow). The vertical red line shows the date on which when epidemic has started.

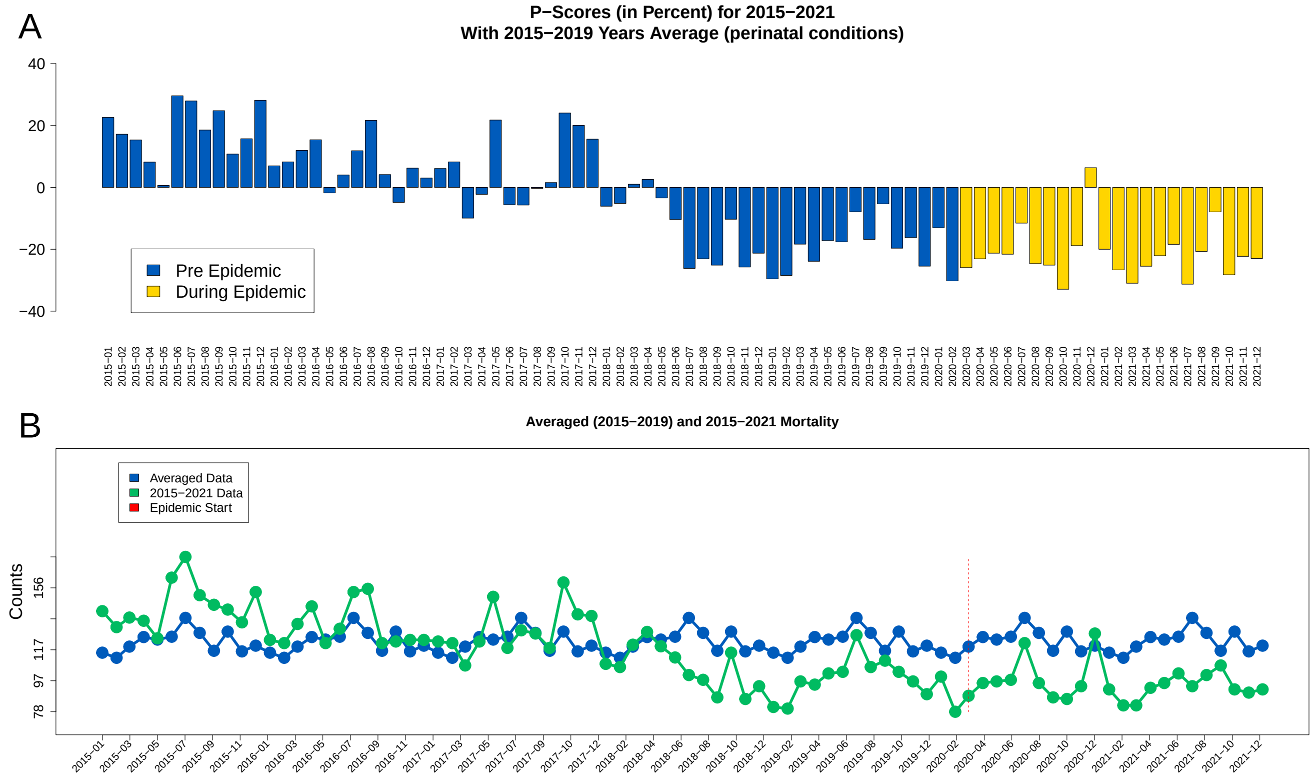

Figure S37. The visual summaries of nonparametric "perinatal conditions" mortality P-scores presented at panel A. Yellow bars represent the epidemic period. The corresponding averaged data (blue) based on the previous five (panel B) years are presented along the reported data for $2020-2021$ (yellow). The vertical red line shows the date on which when epidemic has started.

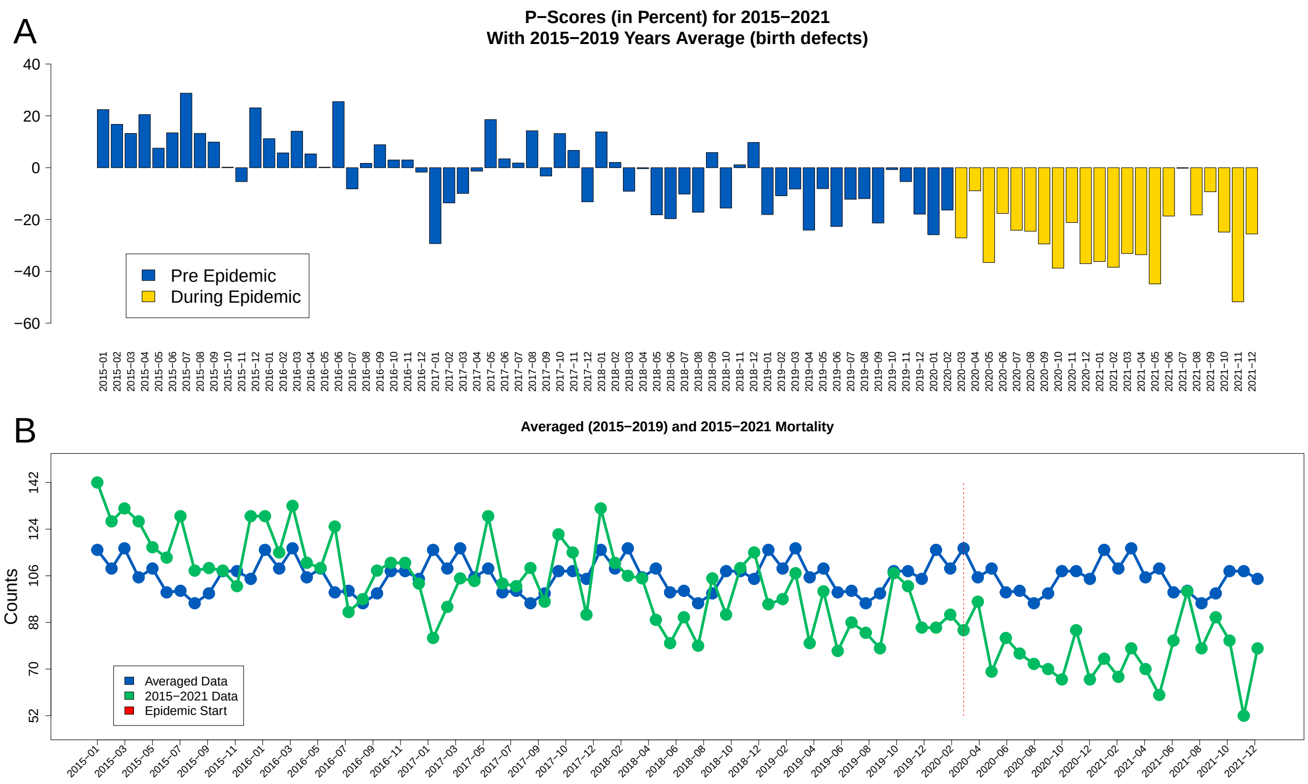

Figure S38. The visual summaries of nonparametric "birth defects and chromosome anomalies" mortality *P*-scores presented at panel A. Yellow bars represent the epidemic period. The corresponding averaged data (blue) based on the previous five (panel B) years are presented along the reported data for 2020 – 2021 (yellow). The vertical red line shows the date on which when epidemic has started.

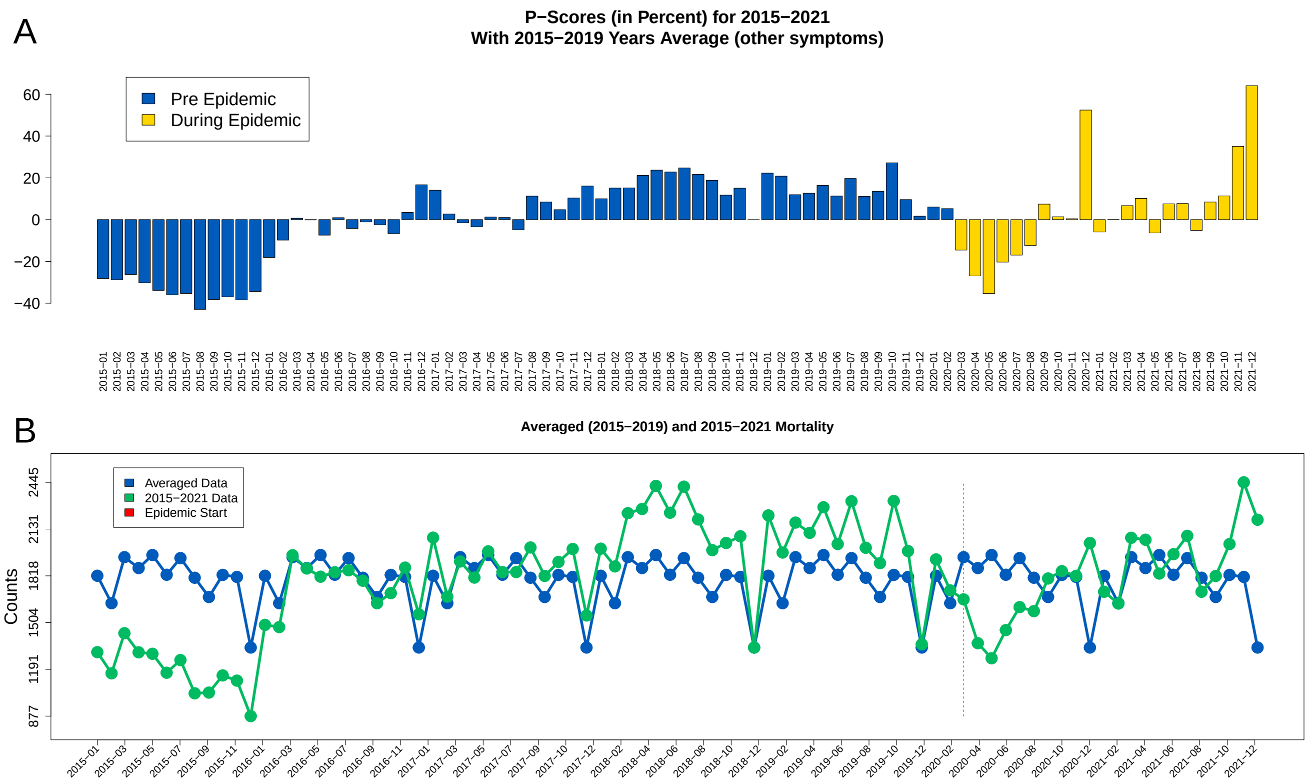

Figure S39. The visual summaries of nonparametric "other symptoms not classified as any above" mortality *P*-scores presented at panel A. Yellow bars represent the epidemic period. The corresponding averaged data (blue) based on the previous five (panel B) years are presented along the reported data for 2020 – 2021 (yellow). The vertical red line shows the date on which when epidemic has started.

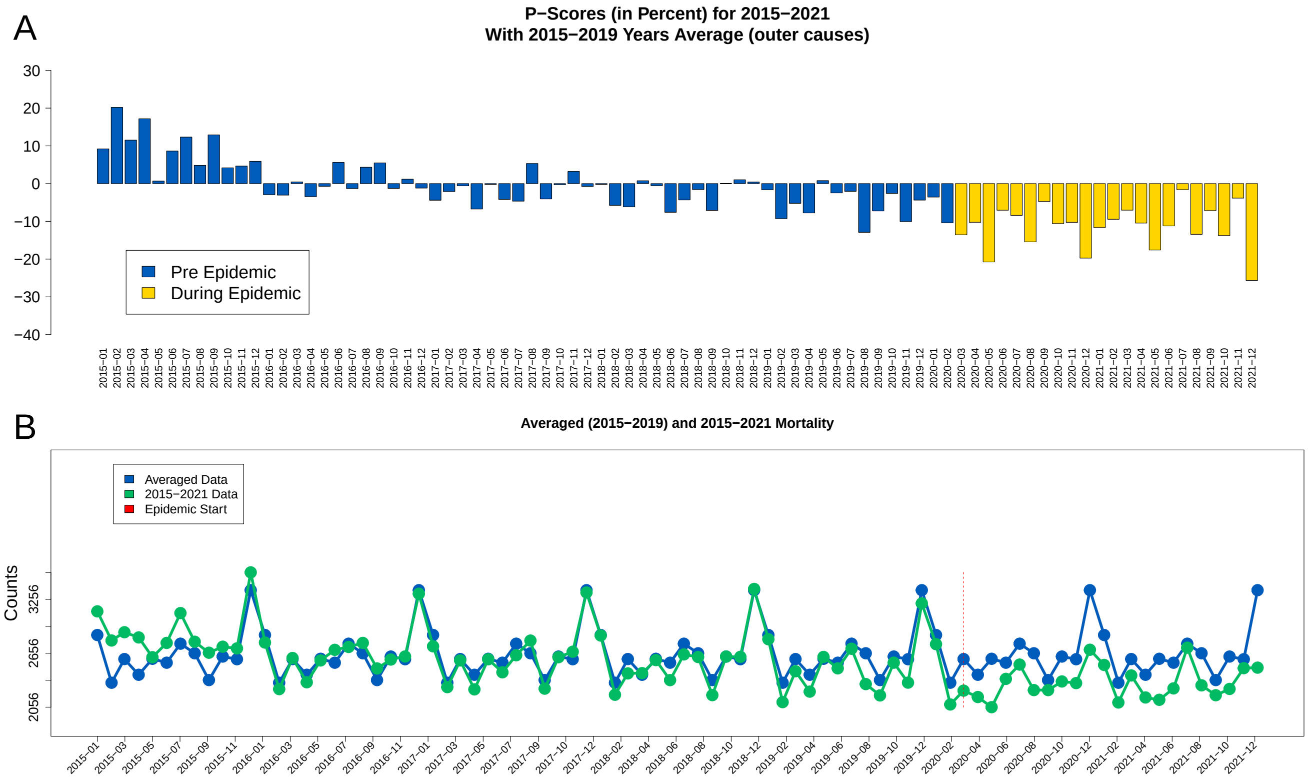

Figure S40. The visual summaries of nonparametric "outer causes" mortality *P*-scores presented at panel A. Yellow bars represent the epidemic period. The corresponding averaged data (blue) based on the previous five (panel B) years are presented along the reported data for 2020 – 2021 (yellow). The vertical red line shows the date on which when epidemic has started.

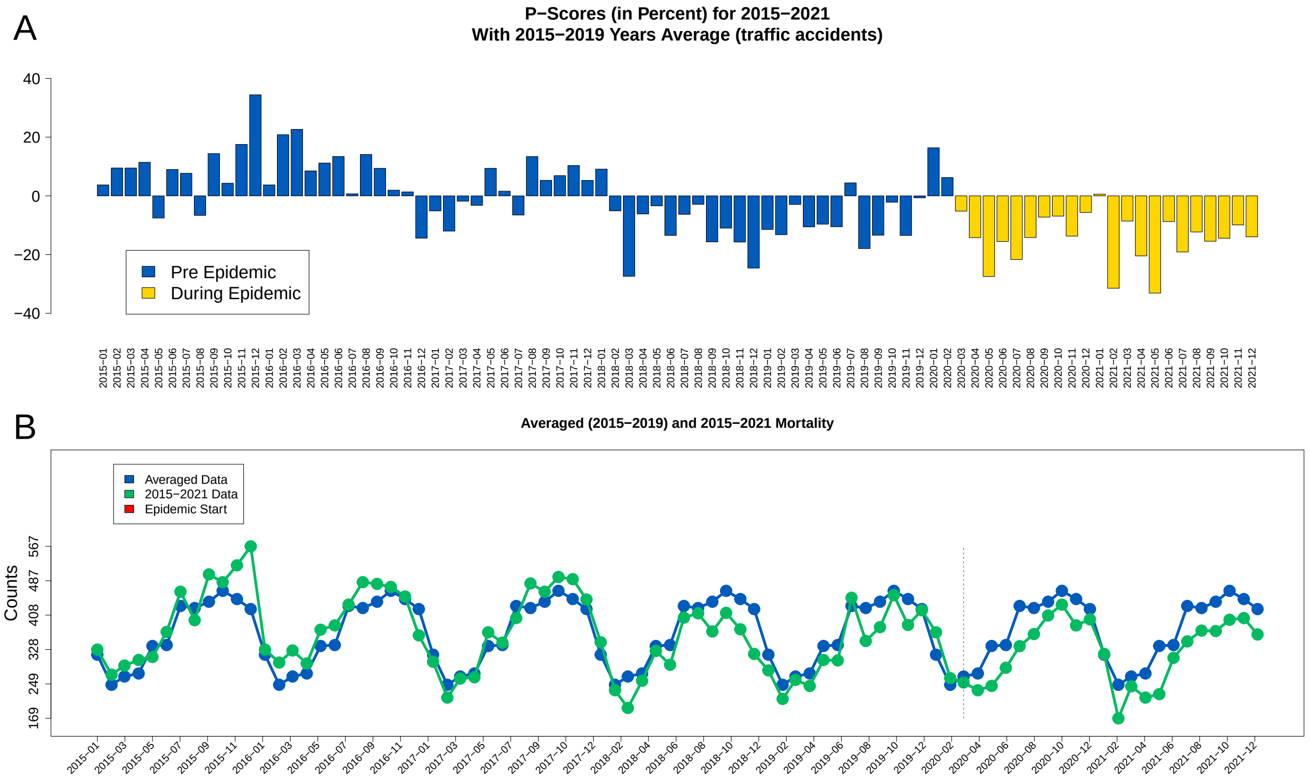

Figure S41. The visual summaries of nonparametric "traffic accidents" mortality *P*-scores presented at panel A. Yellow bars represent the epidemic period. The corresponding averaged data (blue) based on the previous five (panel B) years are presented along the reported data for 2020 – 2021 (yellow). The vertical red line shows the date on which when epidemic has started.

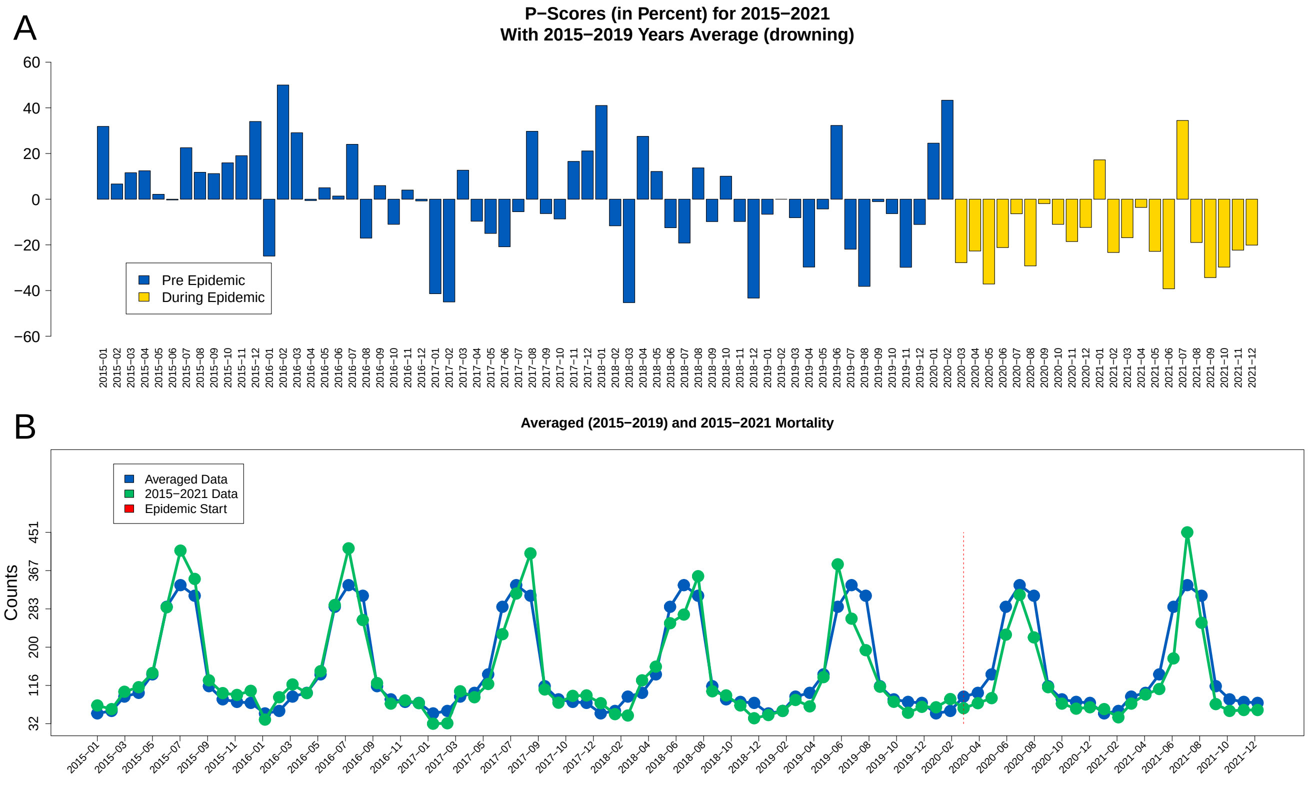

Figure S42. The visual summaries of nonparametric "drowning" mortality *P*-scores presented at panel A. Yellow bars represent the epidemic period. The corresponding averaged data (blue) based on the previous five (panel B) years are presented along the reported data for 2020 – 2021 (yellow). The vertical red line shows the date on which when epidemic has started.

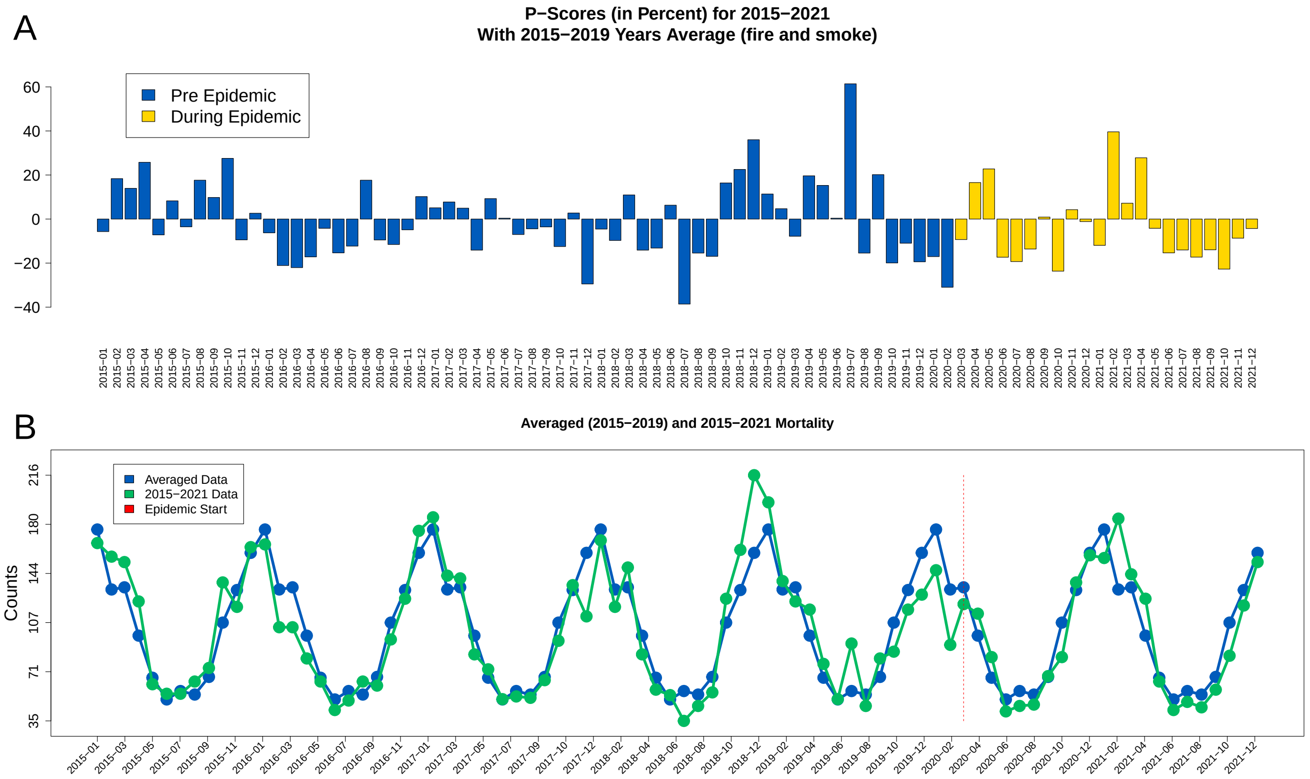

Figure S43. The visual summaries of nonparametric "fire and smoke" mortality *P*-scores presented at panel A. Yellow bars represent the epidemic period. The corresponding averaged data (blue) based on the previous five (panel B) years are presented along the reported data for 2020 – 2021 (yellow). The vertical red line shows the date on which when epidemic has started.

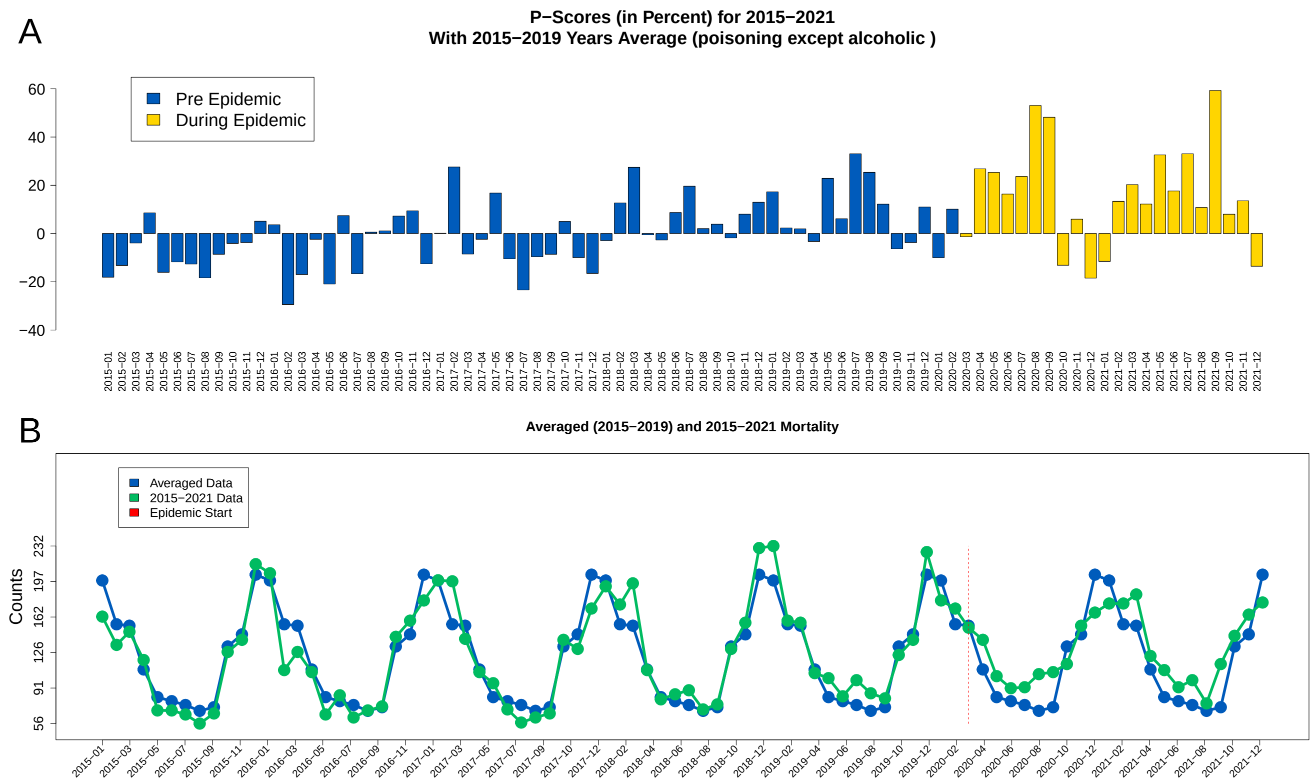

Figure S44. The visual summaries of nonparametric "poisoning, except alcoholic poisoning" mortality *P*-scores presented at panel A. Yellow bars represent the epidemic period. The corresponding averaged data (blue) based on the previous five (panel B) years are presented along the reported data for 2020 – 2021 (yellow). The vertical red line shows the date on which when epidemic has started.

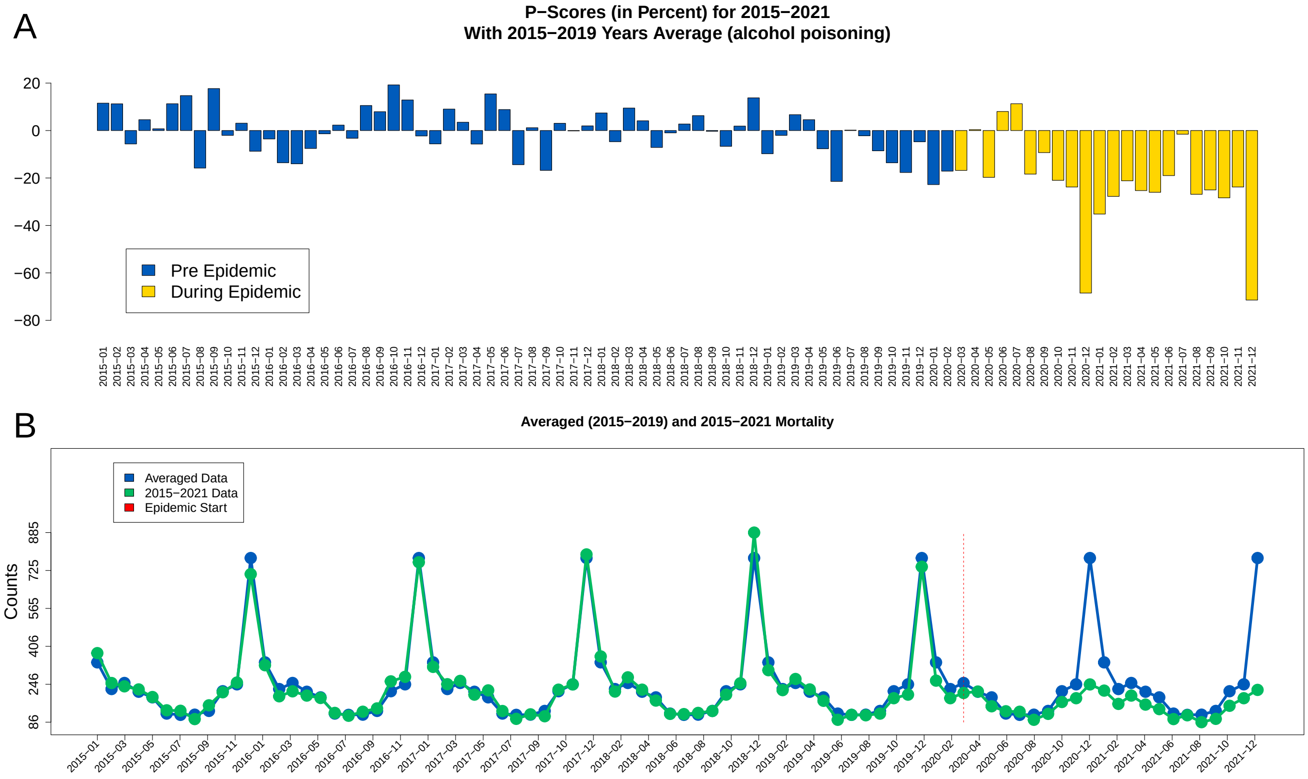

Figure S45. The visual summaries of nonparametric "alcoholic poisoning" mortality *P*-scores presented at panel A. Yellow bars represent the epidemic period. The corresponding averaged data (blue) based on the previous five (panel B) years are presented along the reported data for 2020 – 2021 (yellow). The vertical red line shows the date on which when epidemic has started.

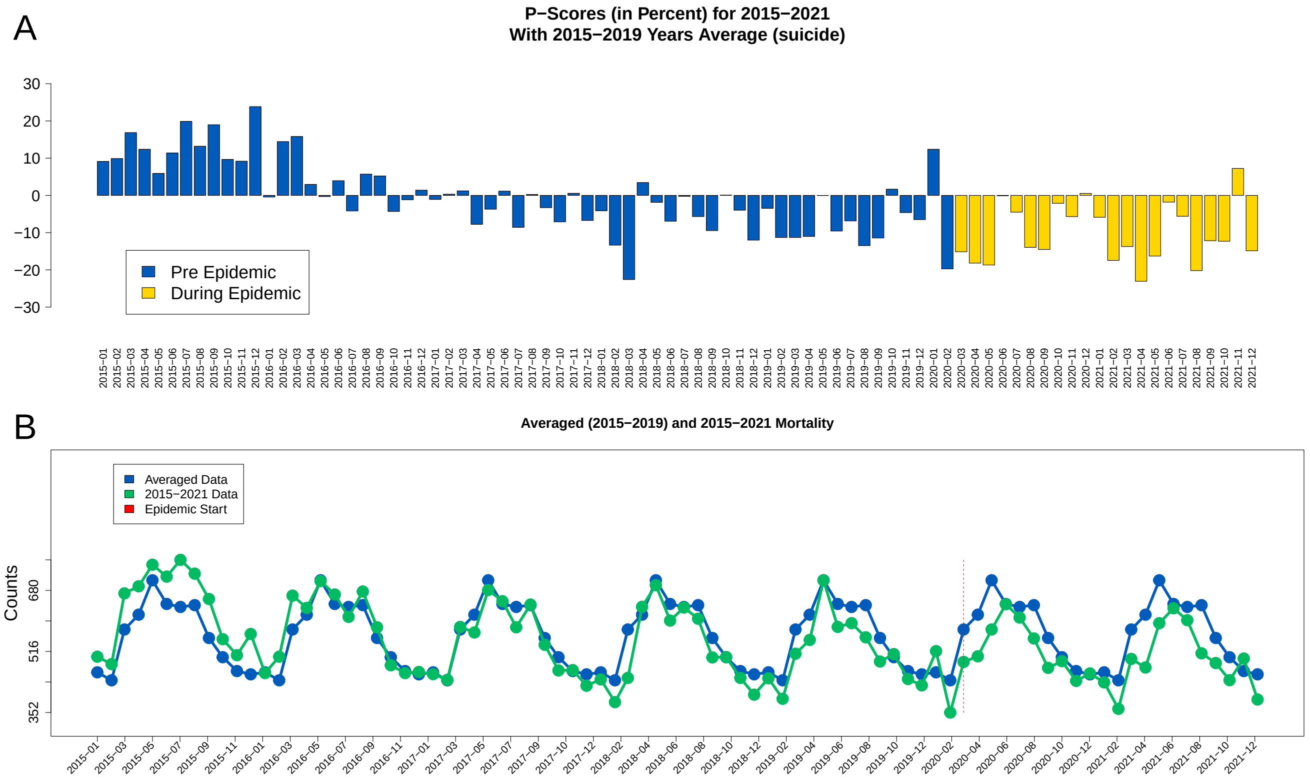

Figure S46. The visual summaries of nonparametric "suicide" mortality *P*-scores presented at panel A. Yellow bars represent the epidemic period. The corresponding averaged data (blue) based on the previous five (panel B) years are presented along the reported data for 2020 – 2021 (yellow). The vertical red line shows the date on which when epidemic has started.

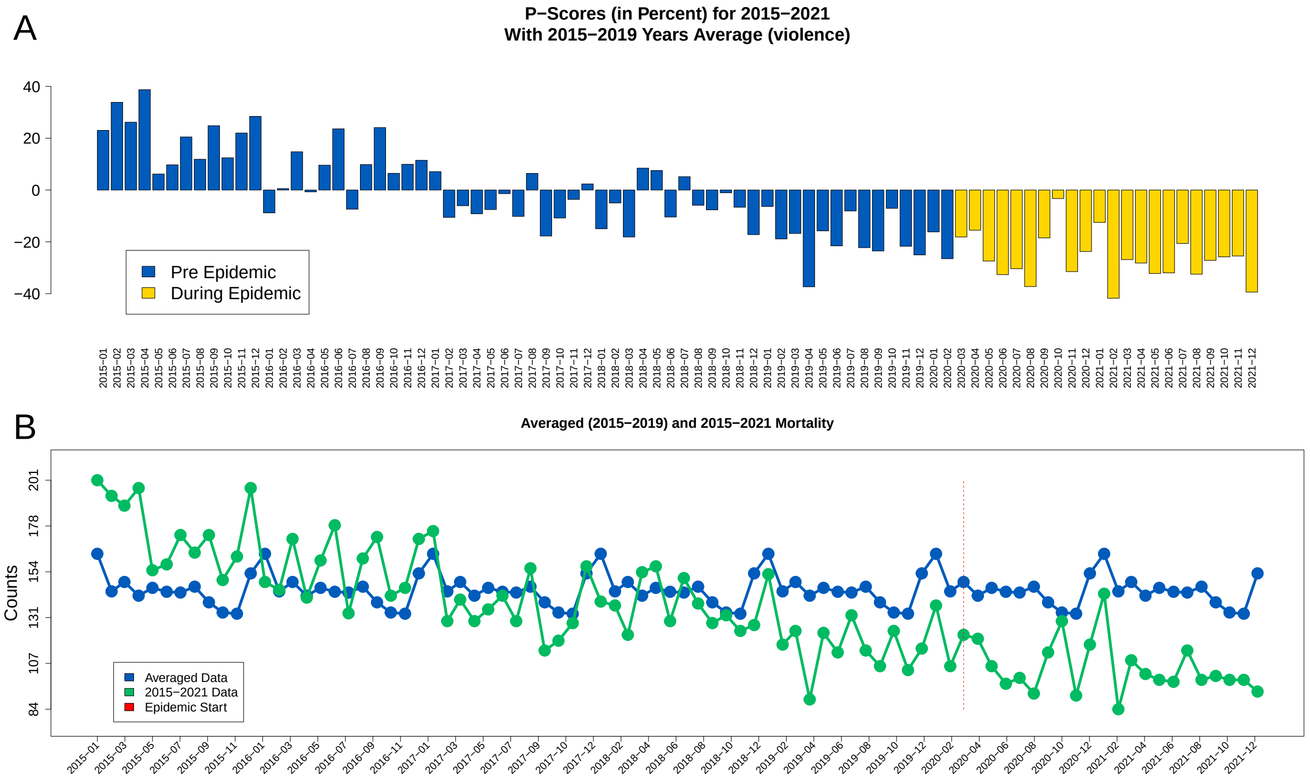

Figure S47. The visual summaries of nonparametric "violence" mortality *P*-scores presented at panel A. Yellow bars represent the epidemic period. The corresponding averaged data (blue) based on the previous five (panel B) years are presented along the reported data for 2020 – 2021 (yellow). The vertical red line shows the date on which when epidemic has started.

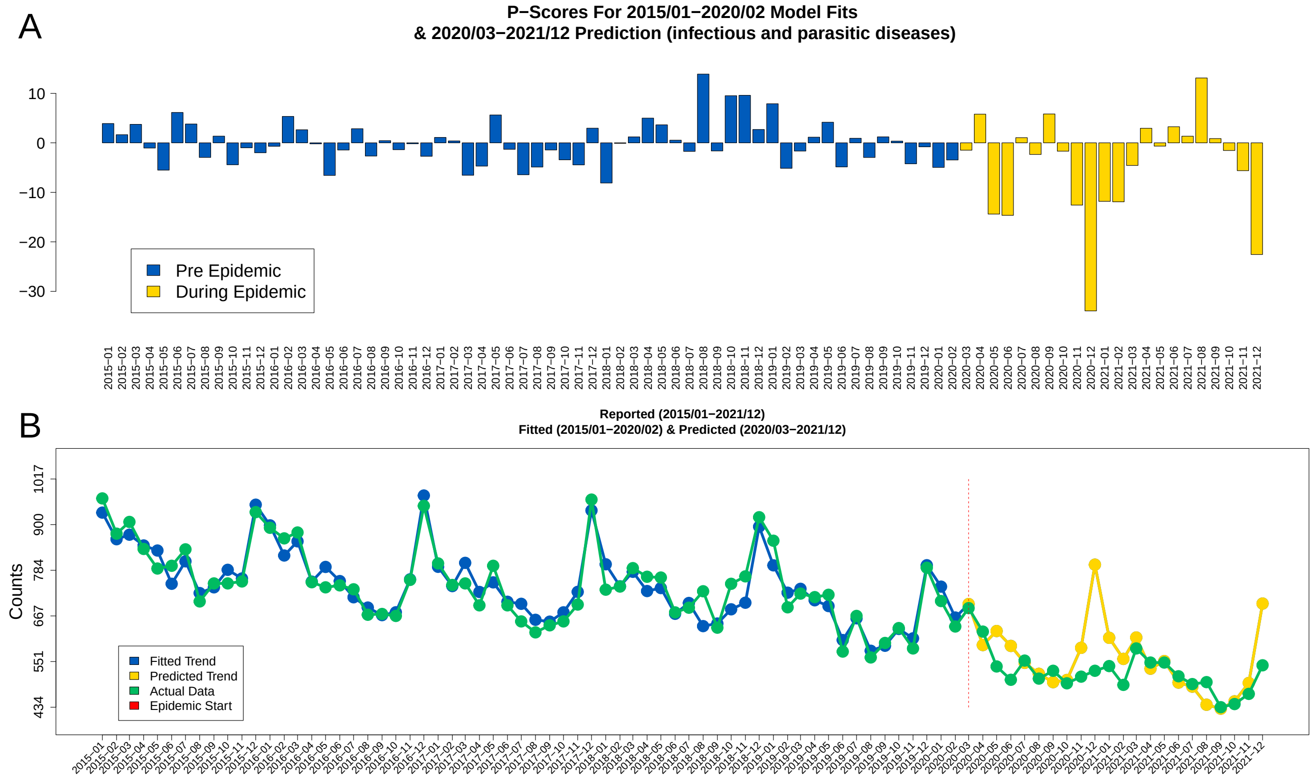

Figure S48. The visual summaries of parametric "infectious and parasitic disease" mortality $\mathscr{P}$-scores based on the Prophet model *without* demographic characteristics are presented on panel A. The yellow bars represent the epidemic period. The corresponding Prophet model fits (blue) and predictions (yellow) based on the 2015-2020 data (panel B) are presented along the reported data (green). Vertical red line indicate the epidemic start period.

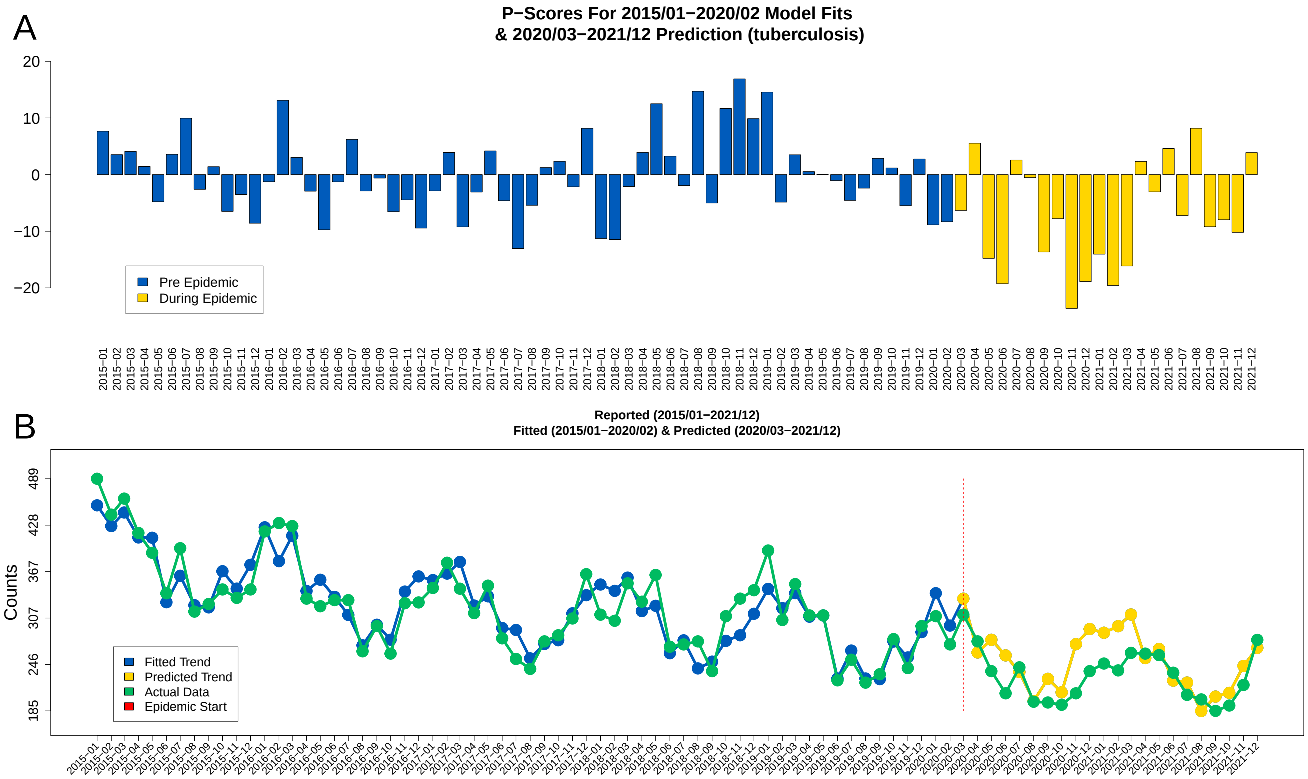

Figure S49. The visual summaries of parametric "tuberculosis" mortality $\mathscr{P}$-scores based on the Prophet model *without* demographic characteristics are presented on panel A. The yellow bars represent the epidemic period. The corresponding Prophet model fits (blue) and predictions (yellow) based on the 2015-2020 data (panel B) are presented along the reported data (green). Vertical red line indicate the epidemic start period.

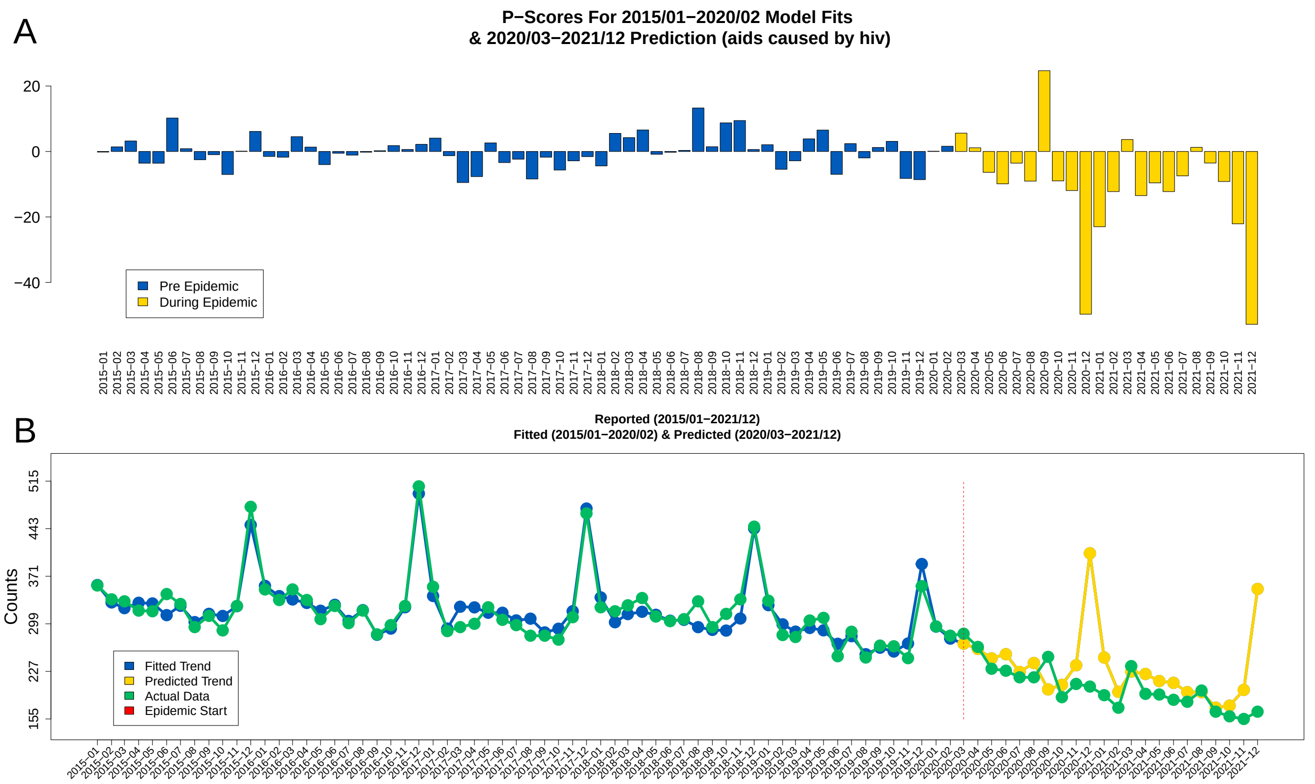

Figure S50. The visual summaries of parametric "AIDS cause by HIV" mortality $\mathscr{P}$-scores based on the Prophet model *without* demographic characteristics are presented on panel A. The yellow bars represent the epidemic period. The corresponding Prophet model fits (blue) and predictions (yellow) based on the 2015-2020 data (panel B) are presented along the reported data (green). Vertical red line indicate the epidemic start period.

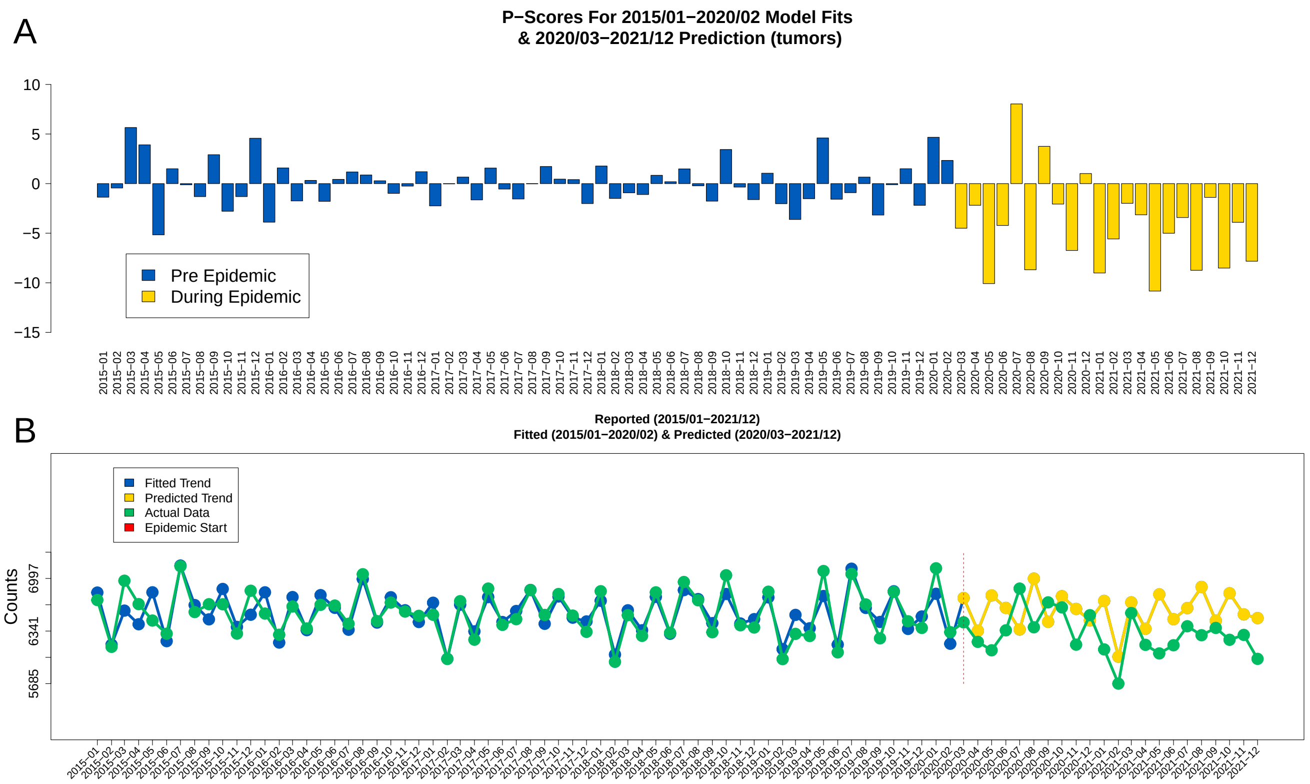

Figure S51. The visual summaries of parametric "tumors" mortality $\mathscr{P}$-scores based on the Prophet model *without* demographic characteristics are presented on panel A. The yellow bars represent the epidemic period. The corresponding Prophet model fits (blue) and predictions (yellow) based on the 2015-2020 data (panel B) are presented along the reported data (green). Vertical red line indicate the epidemic start period.

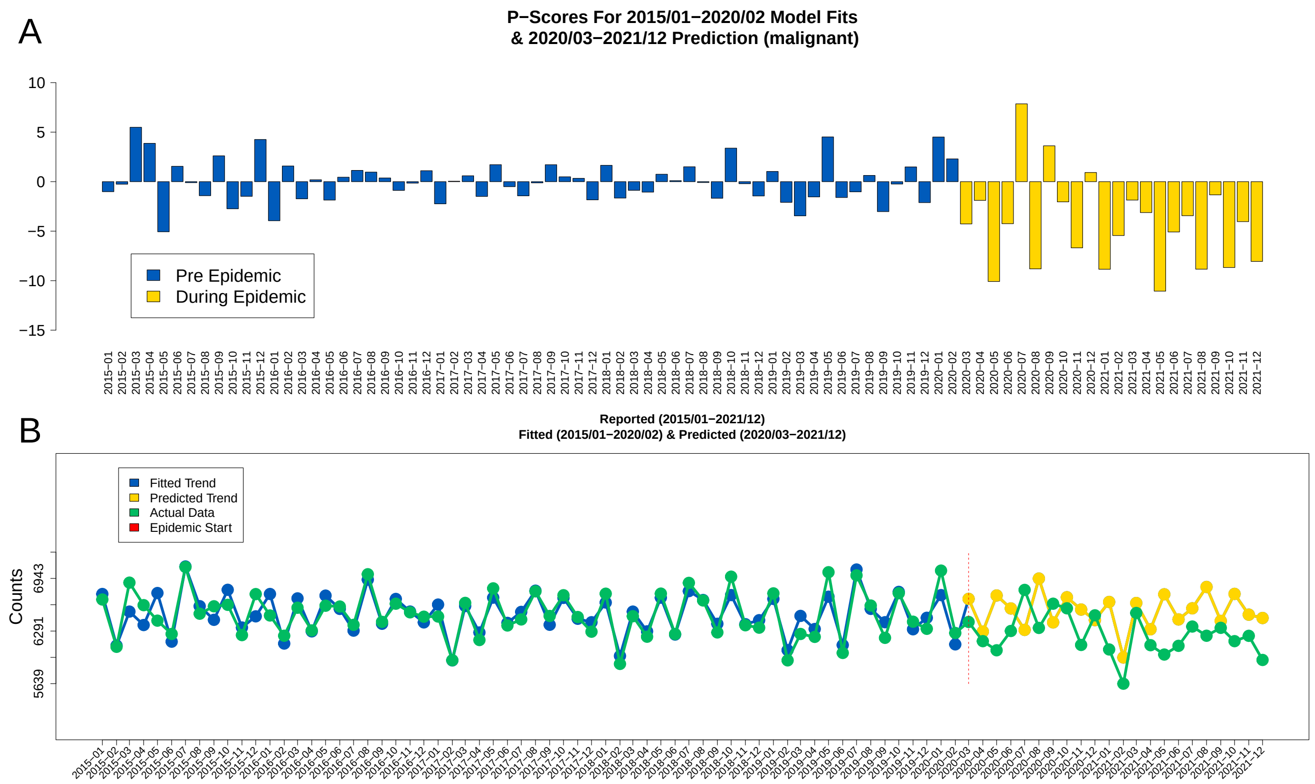

Figure S52. The visual summaries of parametric "malignant tumors" mortality $\mathscr{P}$-scores based on the Prophet model *without* demographic characteristics are presented on panel A. The yellow bars represent the epidemic period. The corresponding Prophet model fits (blue) and predictions (yellow) based on the 2015-2020 data (panel B) are presented along the reported data (green). Vertical red line indicate the epidemic start period.

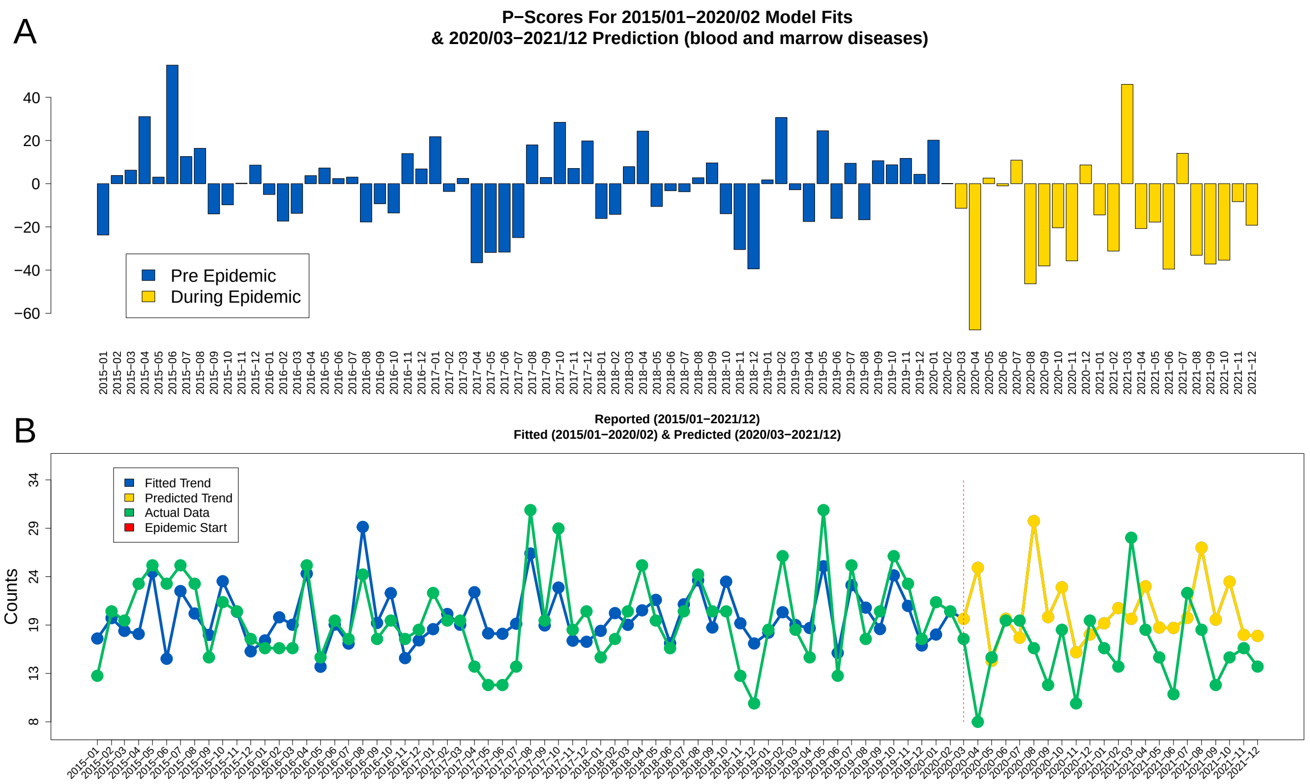

Figure S53. The visual summaries of parametric "blood and marrow diseases" mortality $\mathscr{P}$-scores based on the Prophet model *without* demographic characteristics are presented on panel A. The yellow bars represent the epidemic period. The corresponding Prophet model fits (blue) and predictions (yellow) based on the 2015-2020 data (panel B) are presented along the reported data (green). Vertical red line indicate the epidemic start period.

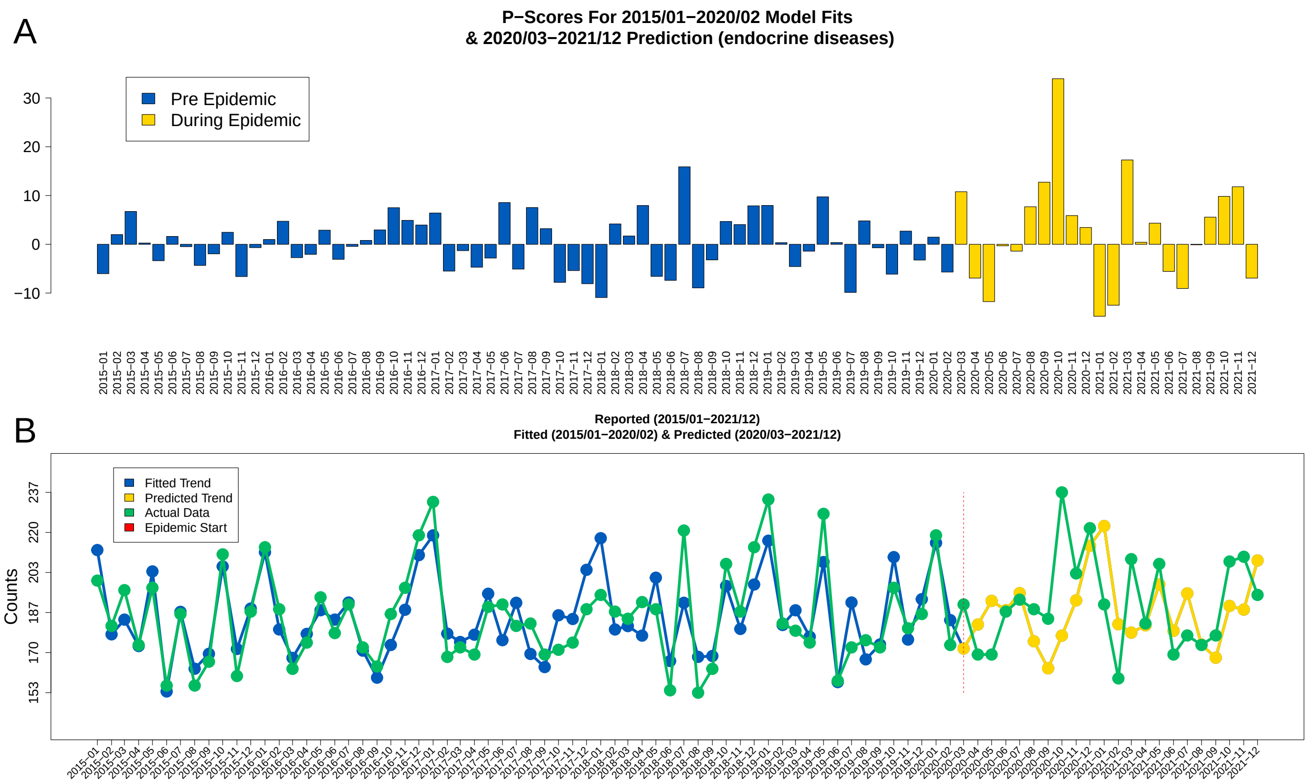

Figure S54. The visual summaries of parametric "endocrine diseases" mortality $\mathscr{P}$-scores based on the Prophet model *without* demographic characteristics are presented on panel A. The yellow bars represent the epidemic period. The corresponding Prophet model fits (blue) and predictions (yellow) based on the 2015-2020 data (panel B) are presented along the reported data (green). Vertical red line indicate the epidemic start period.

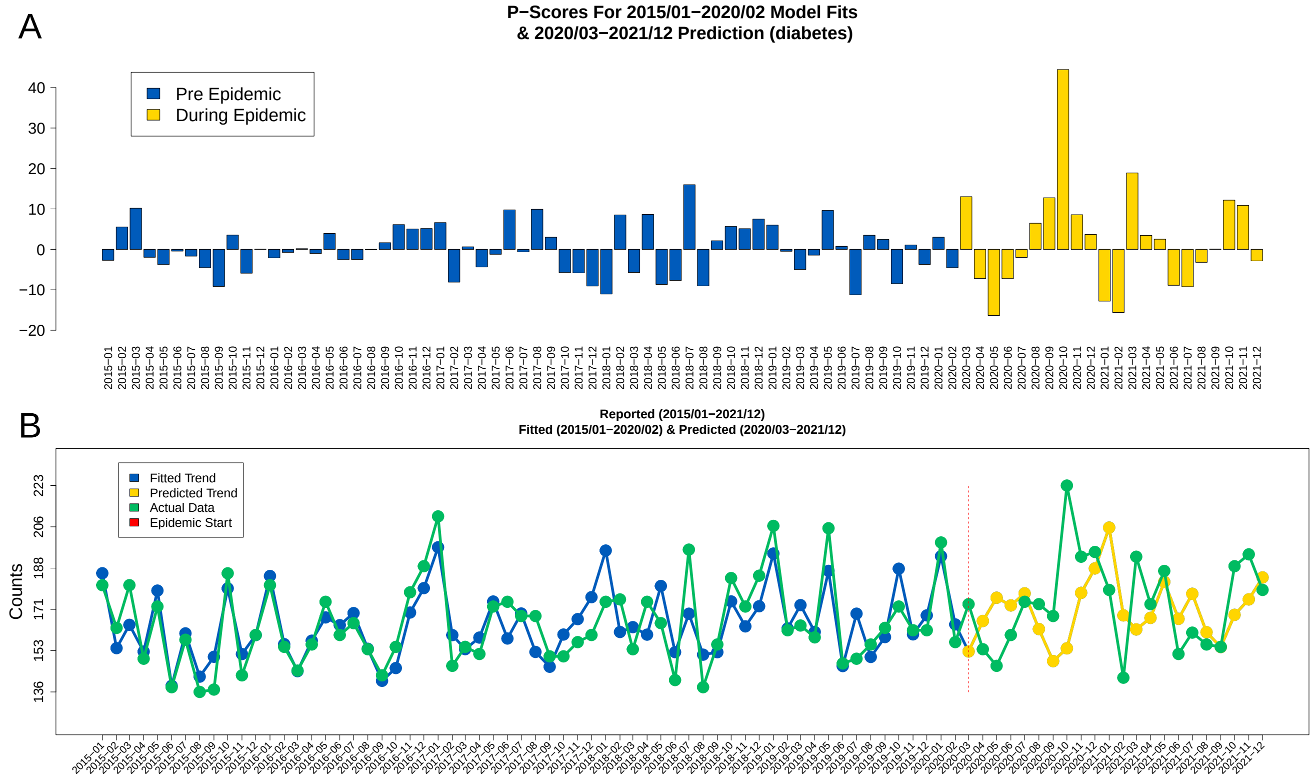

Figure S55. The visual summaries of parametric "diabetes" mortality $\mathscr{P}$-scores based on the Prophet model *without* demographic characteristics are presented on panel A. The yellow bars represent the epidemic period. The corresponding Prophet model fits (blue) and predictions (yellow) based on the 2015-2020 data (panel B) are presented along the reported data (green). Vertical red line indicate the epidemic start period.

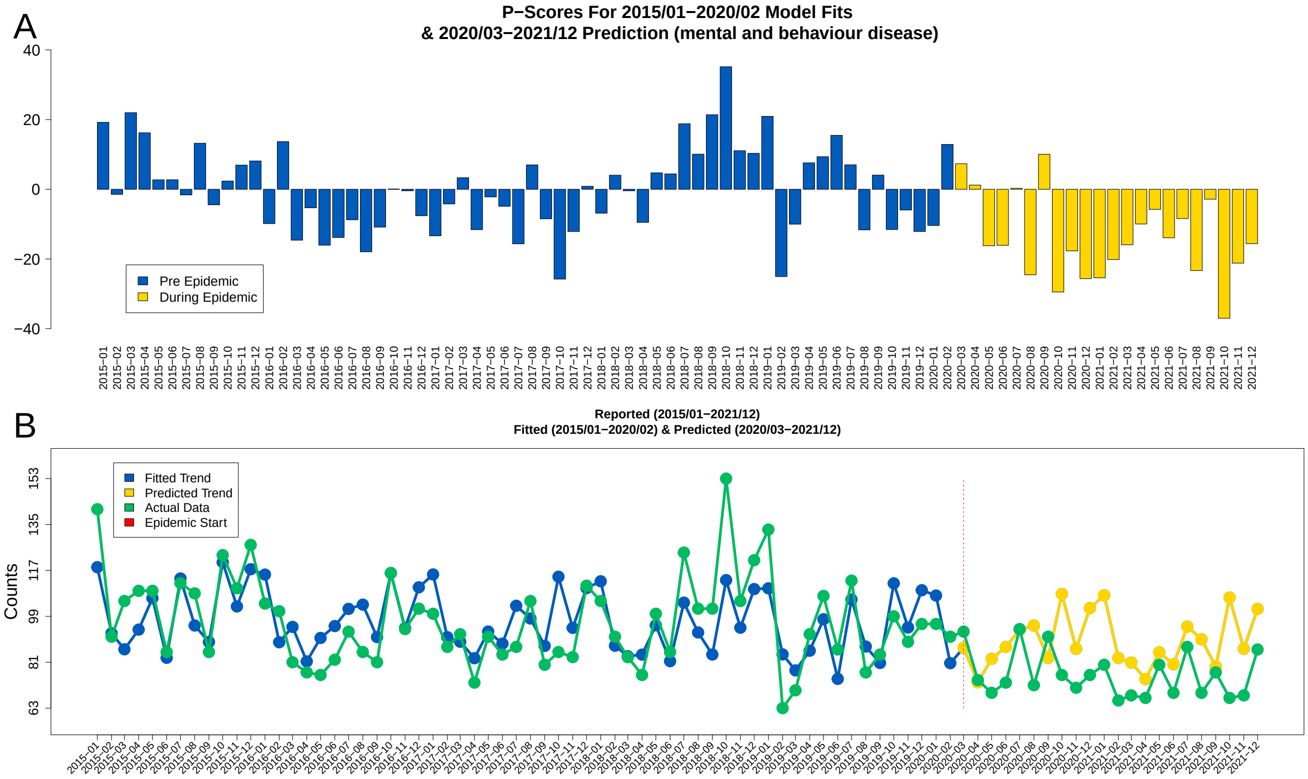

Figure S56. The visual summaries of parametric "mental and behavior disorders" mortality $\mathscr{P}$-scores based on the Prophet model *without* demographic characteristics are presented on panel A. The yellow bars represent the epidemic period. The corresponding Prophet model fits (blue) and predictions (yellow) based on the 2015-2020 data (panel B) are presented along the reported data (green). Vertical red line indicate the epidemic start period.

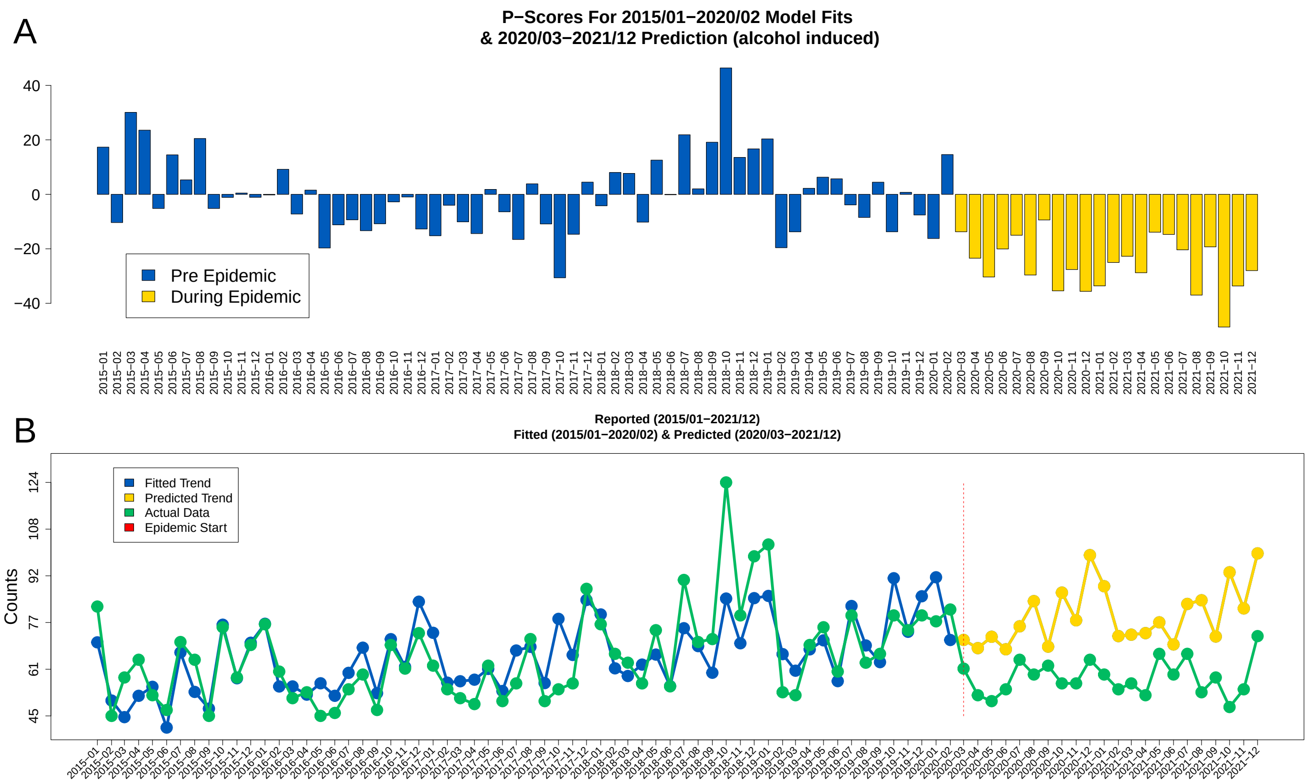

Figure S57. The visual summaries of parametric "alcohol induced mental and behavior disorders" mortality $\mathscr{P}$-scores based on the Prophet model *without* demographic characteristics are presented on panel A. The yellow bars represent the epidemic period. The corresponding Prophet model fits (blue) and predictions (yellow) based on the 2015-2020 data (panel B) are presented along the reported data (green). Vertical red line indicate the epidemic start period.

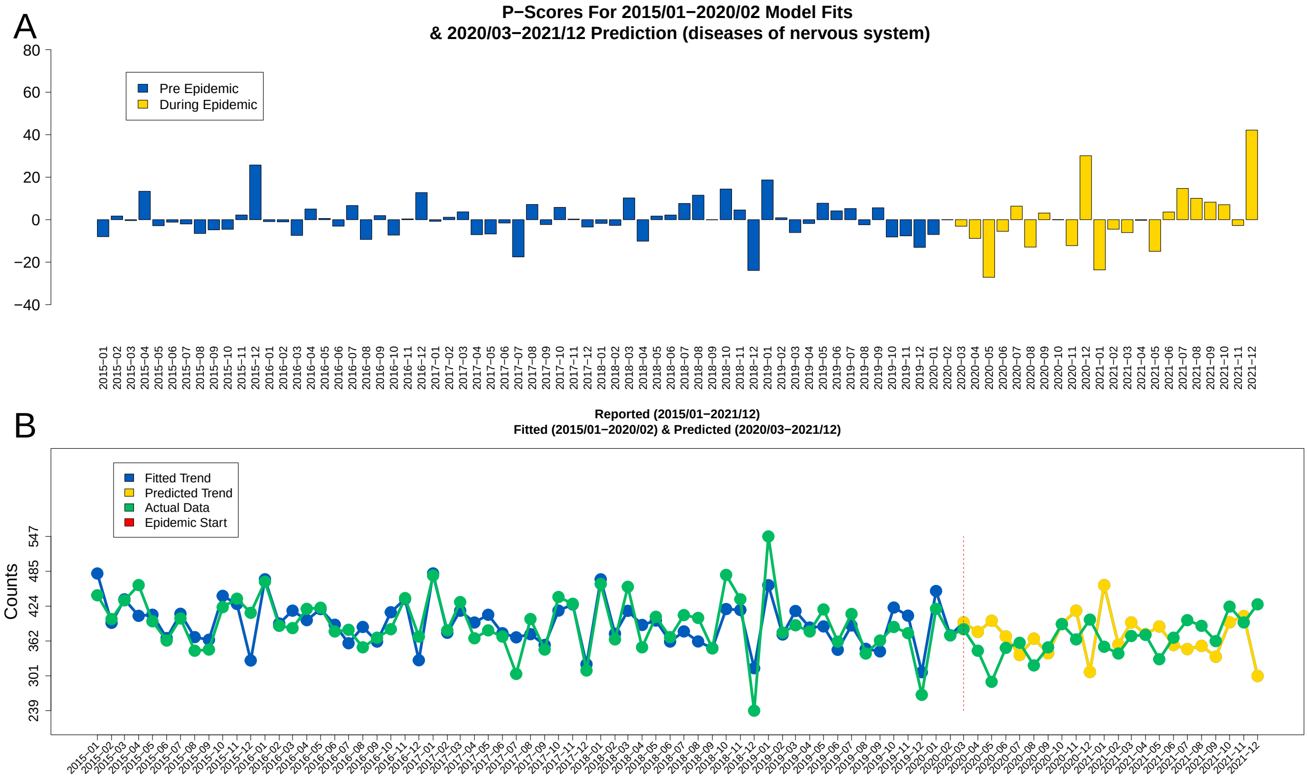

Figure S58. The visual summaries of parametric "diseases of the nervous system" mortality $\mathscr{P}$-scores based on the Prophet model *without* demographic characteristics are presented on panel A. The yellow bars represent the epidemic period. The corresponding Prophet model fits (blue) and predictions (yellow) based on the 2015-2020 data (panel B) are presented along the reported data (green). Vertical red line indicate the epidemic start period.

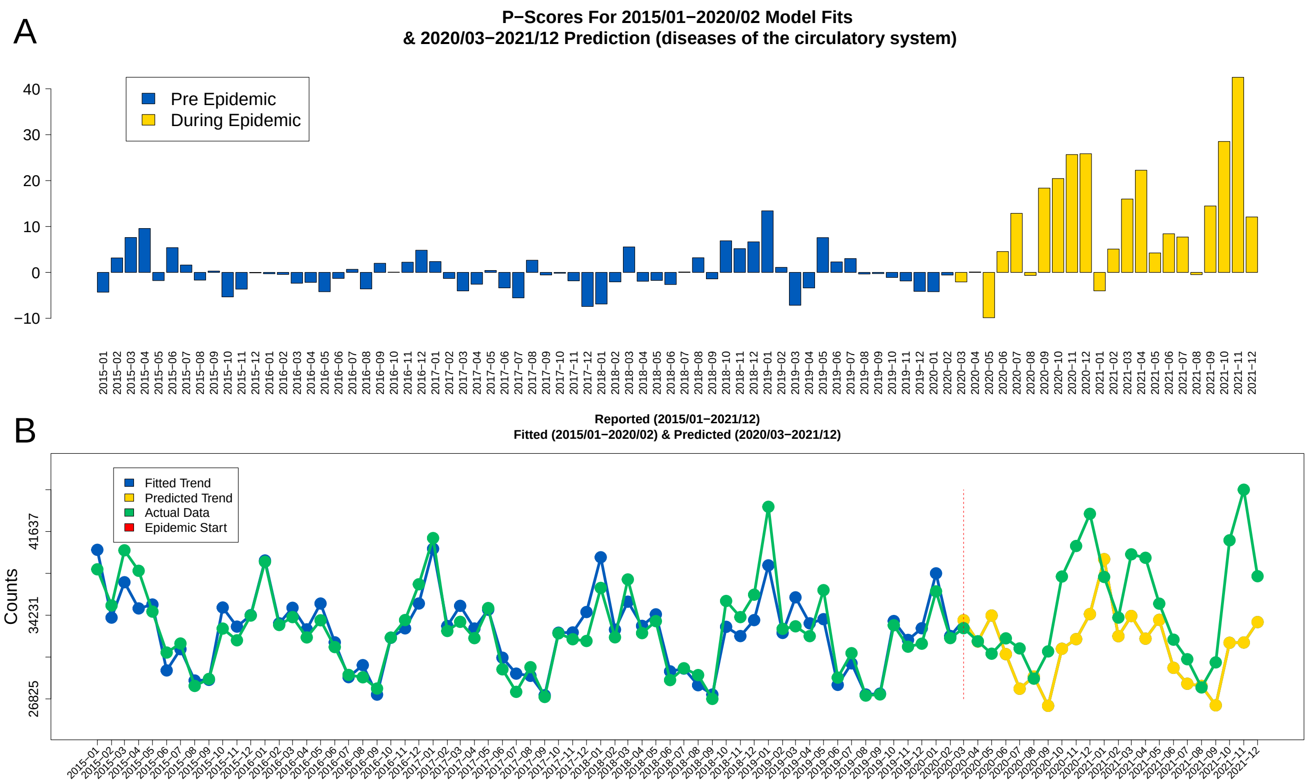

Figure S59. The visual summaries of parametric "diseases of the circulatory system" mortality $\mathscr{P}$-scores based on the Prophet model *without* demographic characteristics are presented on panel A. The yellow bars represent the epidemic period. The corresponding Prophet model fits (blue) and predictions (yellow) based on the 2015-2020 data (panel B) are presented along the reported data (green). Vertical red line indicate the epidemic start period.

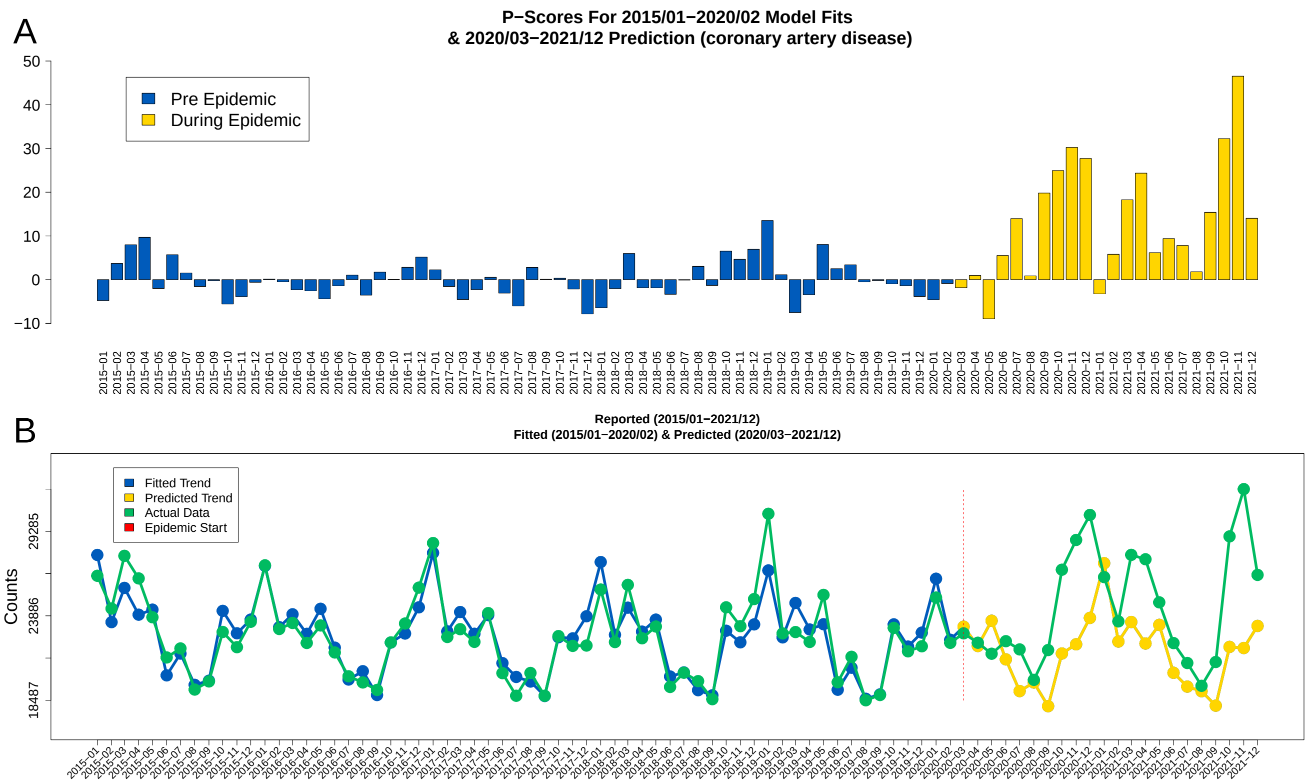

Figure S60. The visual summaries of parametric "coronary artery disease" mortality $\mathscr{P}$-scores based on the Prophet model *without* demographic characteristics are presented on panel A. The yellow bars represent the epidemic period. The corresponding Prophet model fits (blue) and predictions (yellow) based on the 2015-2020 data (panel B) are presented along the reported data (green). Vertical red line indicate the epidemic start period.

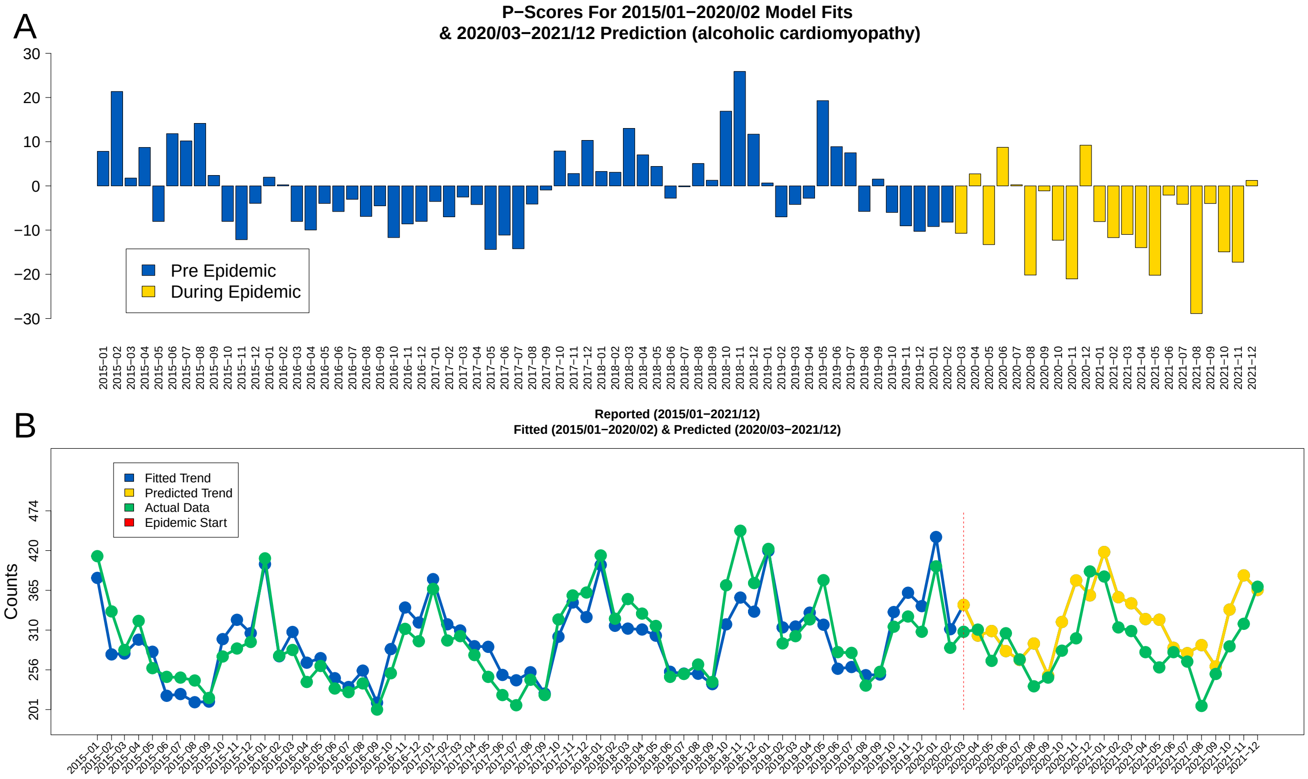

Figure S61. The visual summaries of parametric "alcoholic cardiomyopathy" mortality $\mathscr{P}$-scores based on the Prophet model *without* demographic characteristics are presented on panel A. The yellow bars represent the epidemic period. The corresponding Prophet model fits (blue) and predictions (yellow) based on the 2015-2020 data (panel B) are presented along the reported data (green). Vertical red line indicate the epidemic start period.

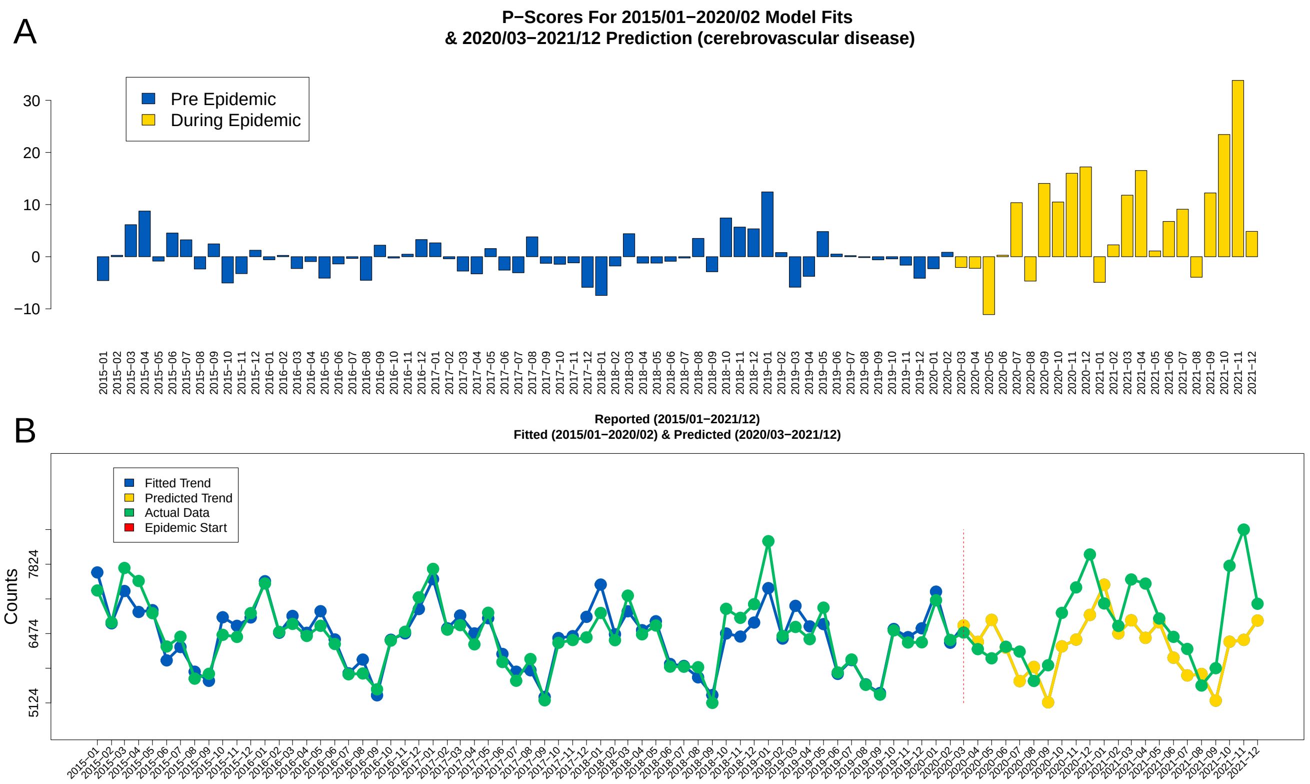

Figure S62. The visual summaries of parametric "cerebrovascular disease" mortality $\mathscr{P}$-scores based on the Prophet model *without* demographic characteristics are presented on panel A. The yellow bars represent the epidemic period. The corresponding Prophet model fits (blue) and predictions (yellow) based on the 2015-2020 data (panel B) are presented along the reported data (green). Vertical red line indicate the epidemic start period.

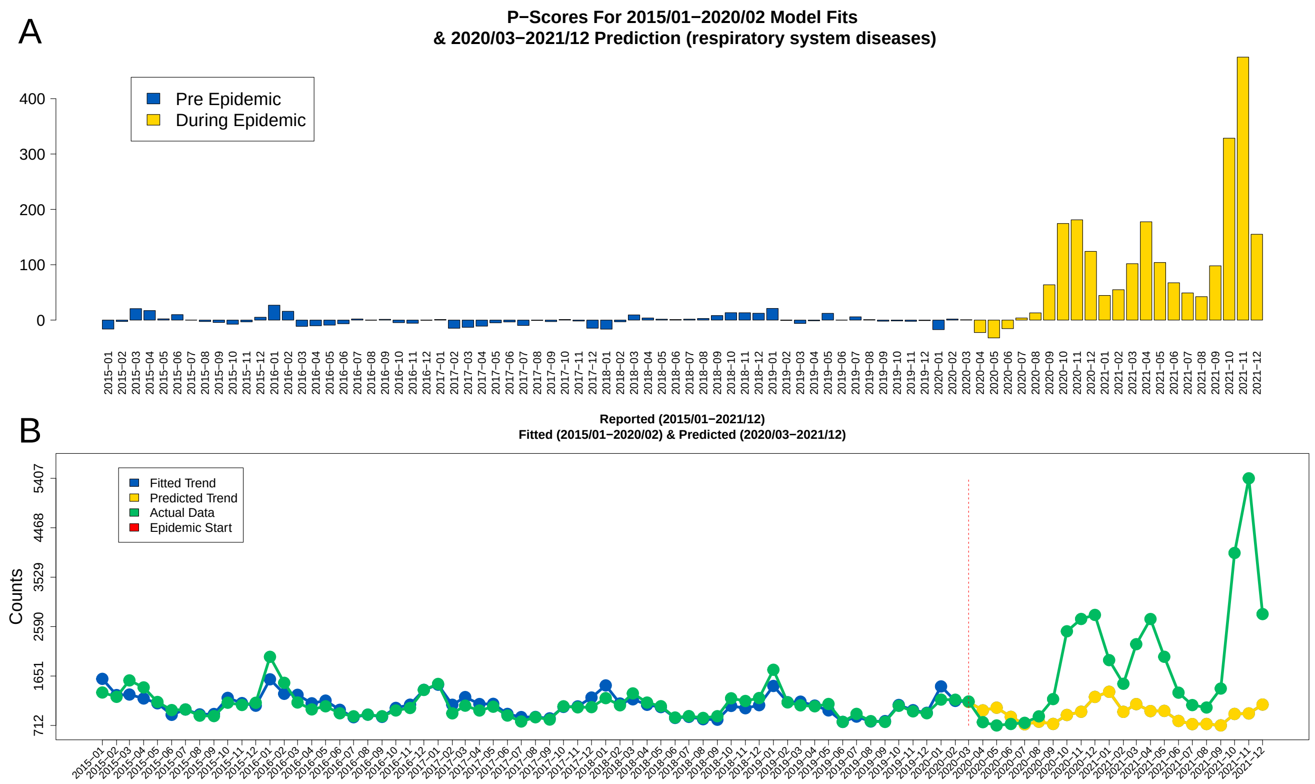

Figure S63. The visual summaries of parametric "respiratory system diseases" mortality $\mathscr{P}$-scores based on the Prophet model *without* demographic characteristics are presented on panel A. The yellow bars represent the epidemic period. The corresponding Prophet model fits (blue) and predictions (yellow) based on the 2015-2020 data (panel B) are presented along the reported data (green). Vertical red line indicate the epidemic start period.

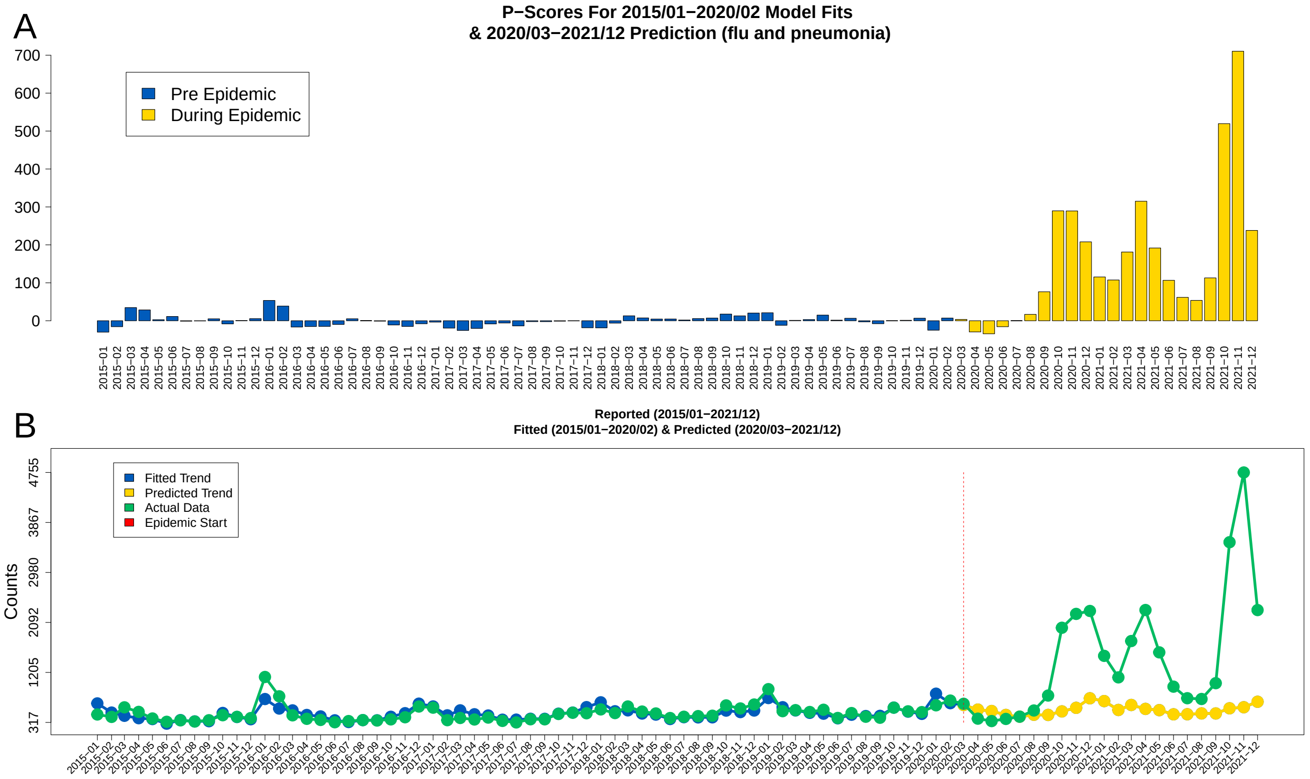

Figure S64. The visual summaries of parametric "flu and pneumonia" mortality $\mathscr{P}$-scores based on the Prophet model *without* demographic characteristics are presented on panel A. The yellow bars represent the epidemic period. The corresponding Prophet model fits (blue) and predictions (yellow) based on the 2015-2020 data (panel B) are presented along the reported data (green). Vertical red line indicate the epidemic start period.

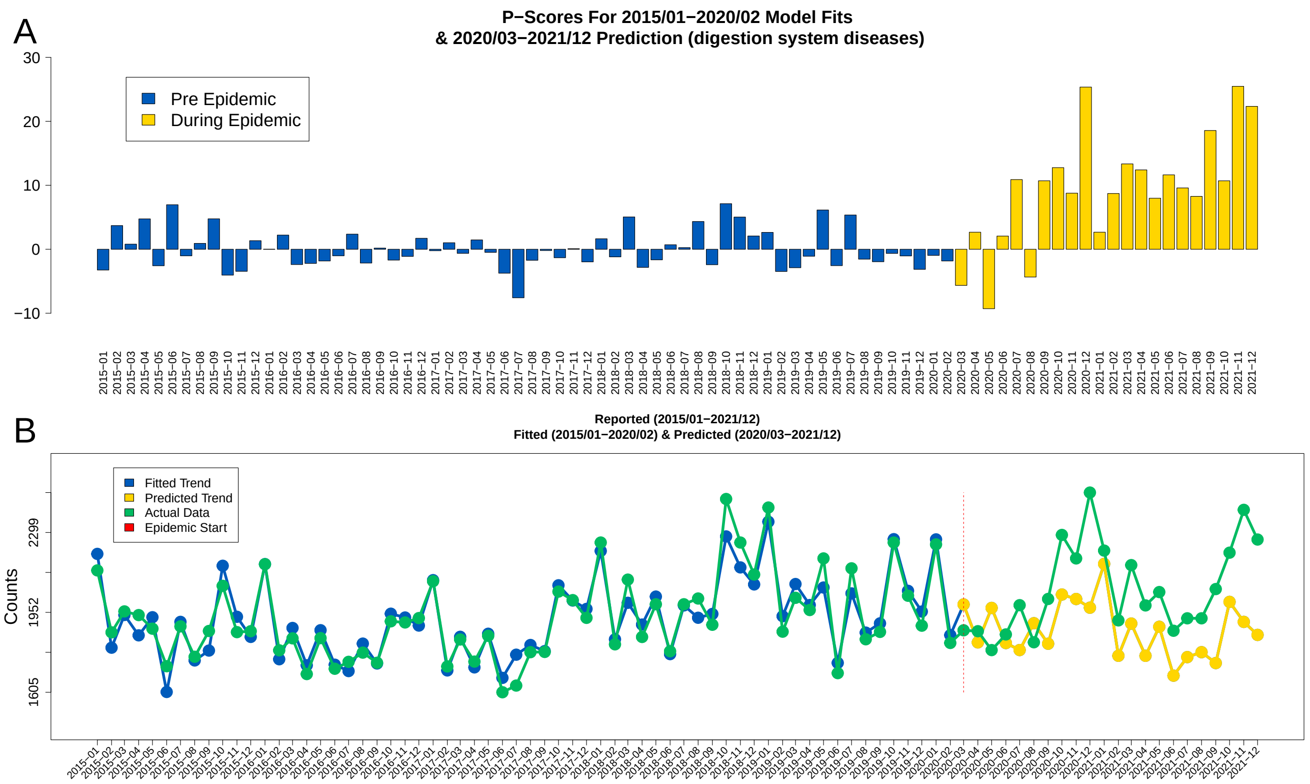

Figure S65. The visual summaries of parametric "digestion system diseases" mortality $\mathscr{P}$-scores based on the Prophet model *without* demographic characteristics are presented on panel A. The yellow bars represent the epidemic period. The corresponding Prophet model fits (blue) and predictions (yellow) based on the 2015-2020 data (panel B) are presented along the reported data (green). Vertical red line indicate the epidemic start period.

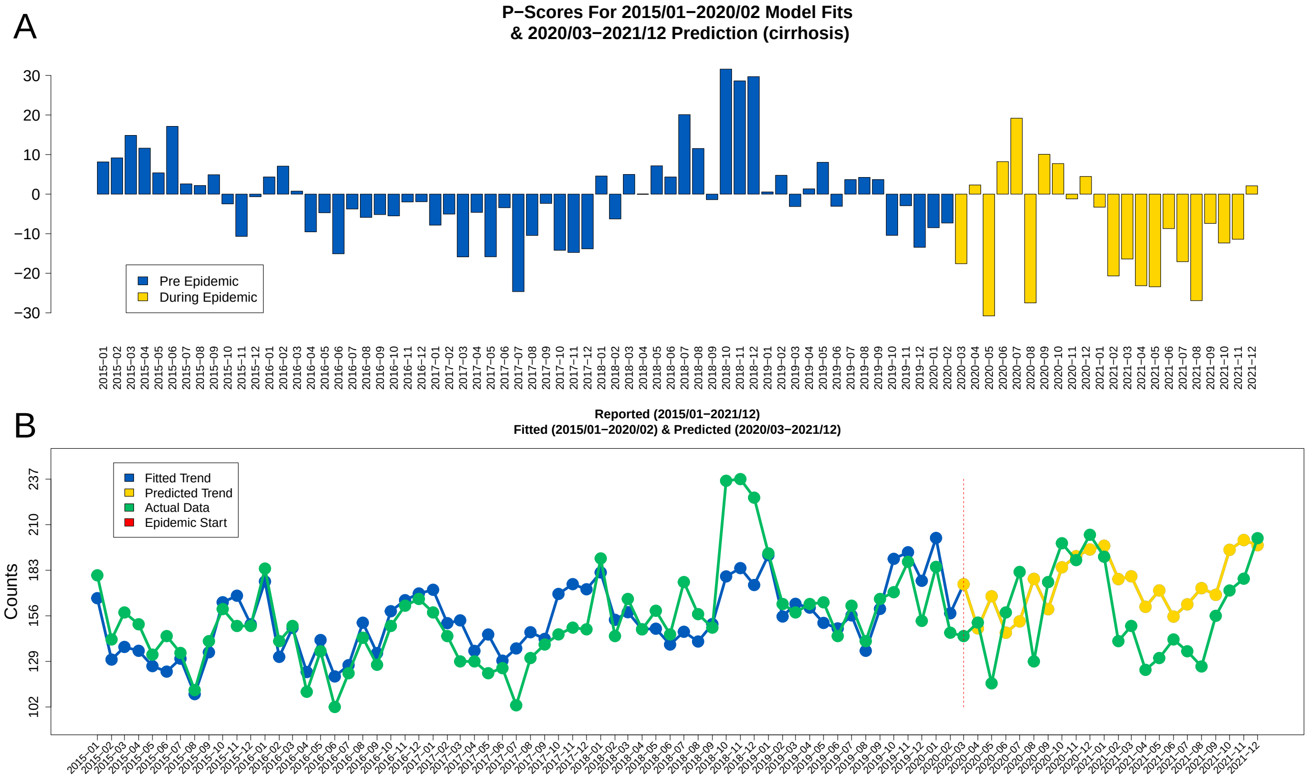

Figure S66. The visual summaries of parametric "cirrhosis" mortality $\mathscr{P}$-scores based on the Prophet model *without* demographic characteristics are presented on panel A. The yellow bars represent the epidemic period. The corresponding Prophet model fits (blue) and predictions (yellow) based on the 2015-2020 data (panel B) are presented along the reported data (green). Vertical red line indicate the epidemic start period.

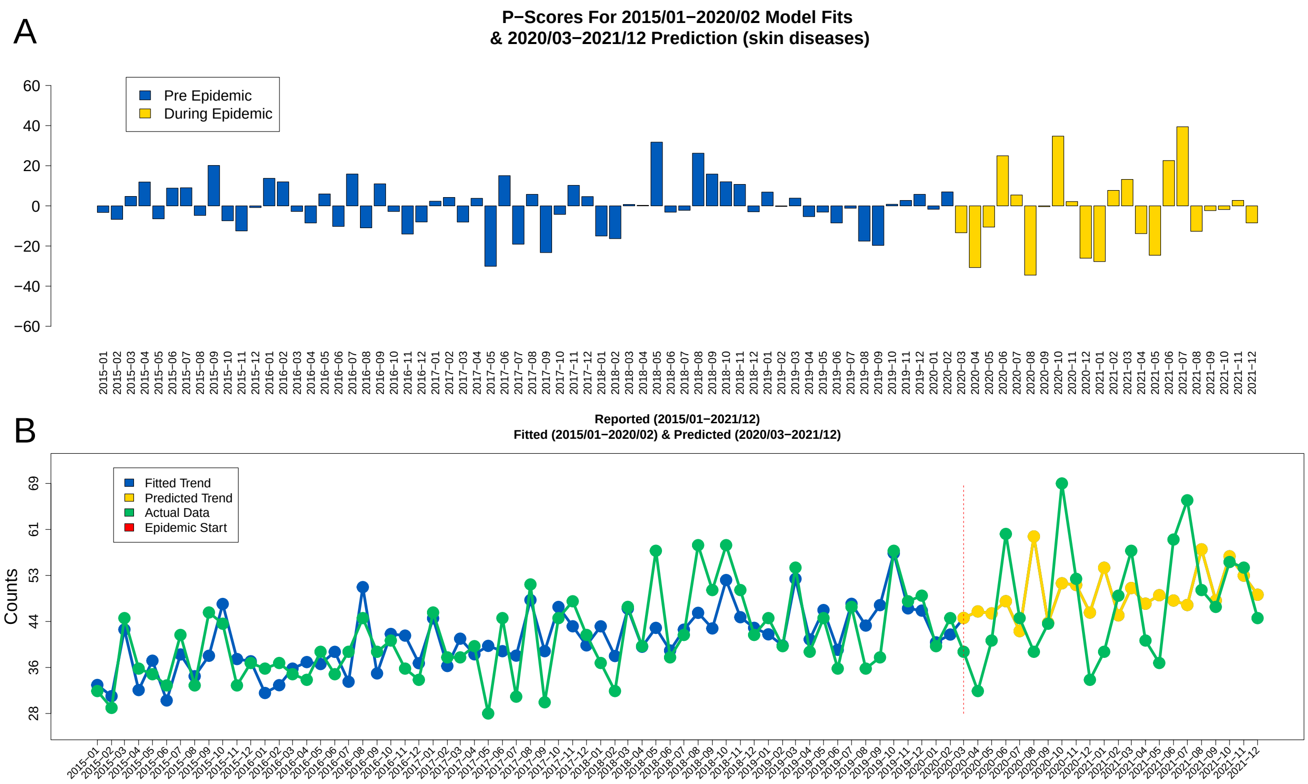

Figure S67. The visual summaries of parametric "skin diseases" mortality $\mathscr{P}$-scores based on the Prophet model *without* demographic characteristics are presented on panel A. The yellow bars represent the epidemic period. The corresponding Prophet model fits (blue) and predictions (yellow) based on the 2015-2020 data (panel B) are presented along the reported data (green). Vertical red line indicate the epidemic start period.

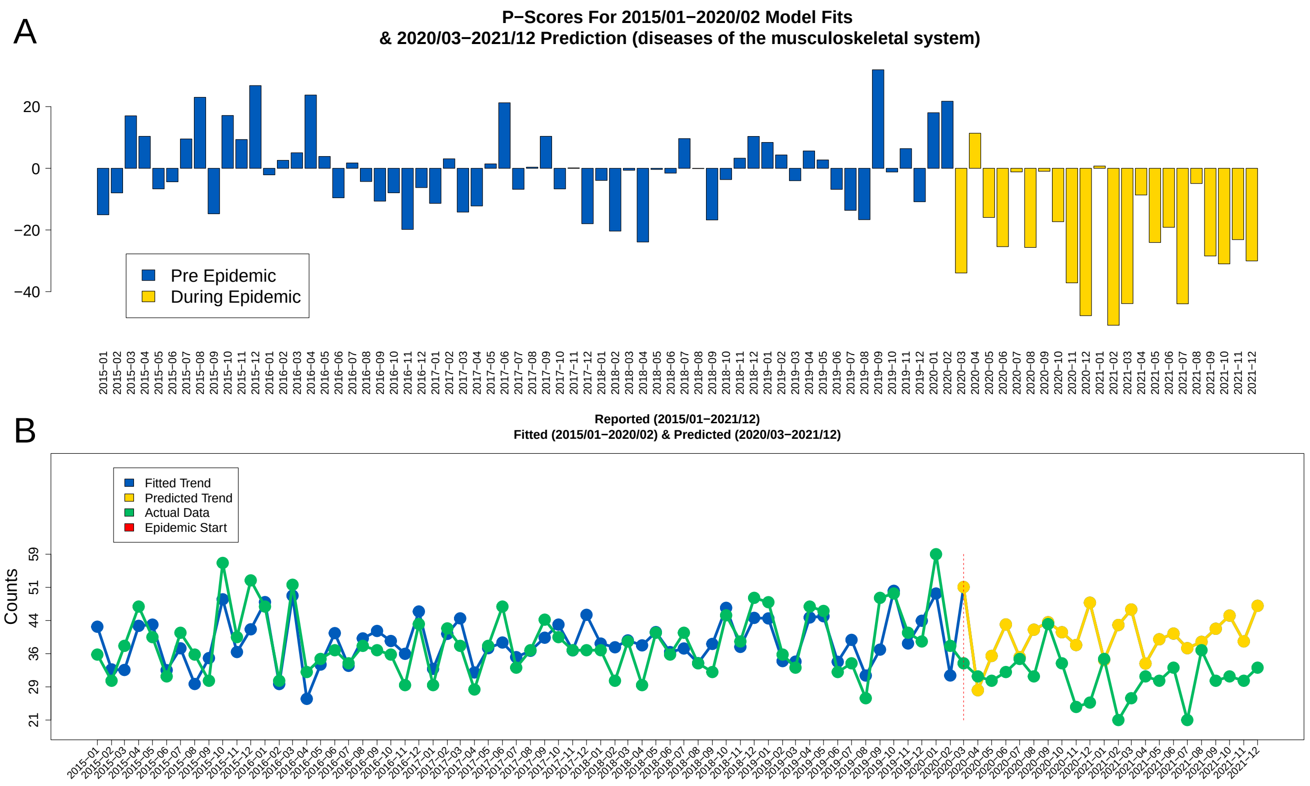

Figure S68. The visual summaries of parametric "diseases of the musculoskeletal system" mortality $\mathscr{P}$-scores based on the Prophet model *without* demographic characteristics are presented on panel A. The yellow bars represent the epidemic period. The corresponding Prophet model fits (blue) and predictions (yellow) based on the 2015-2020 data (panel B) are presented along the reported data (green). Vertical red line indicate the epidemic start period.

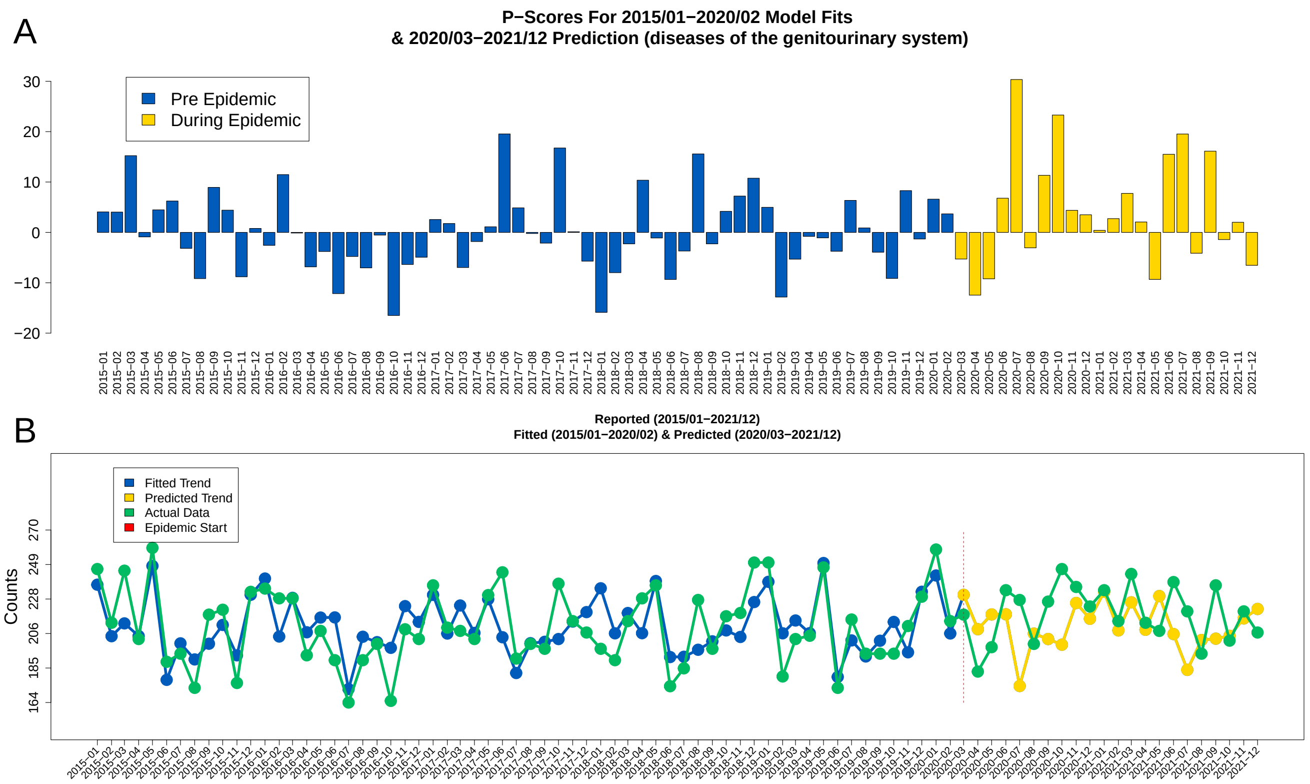

Figure S69. The visual summaries of parametric "diseases of the genitourinary system" mortality $\mathscr{P}$-scores based on the Prophet model *without* demographic characteristics are presented on panel A. The yellow bars represent the epidemic period. The corresponding Prophet model fits (blue) and predictions (yellow) based on the 2015-2020 data (panel B) are presented along the reported data (green). Vertical red line indicate the epidemic start period.

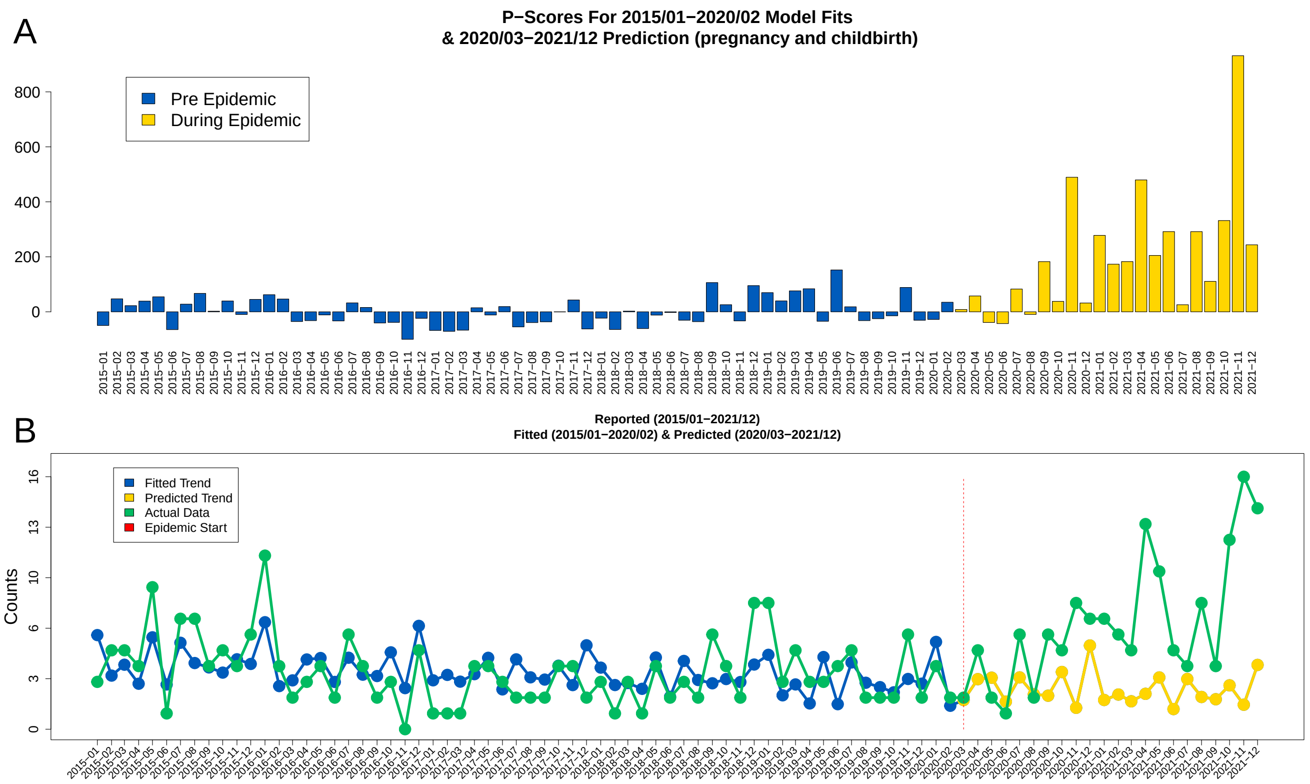

Figure S70. The visual summaries of parametric "pregnancy and childbirth" mortality $\mathscr{P}$-scores based on the Prophet model *without* demographic characteristics are presented on panel A. The yellow bars represent the epidemic period. The corresponding Prophet model fits (blue) and predictions (yellow) based on the 2015-2020 data (panel B) are presented along the reported data (green). Vertical red line indicate the epidemic start period.

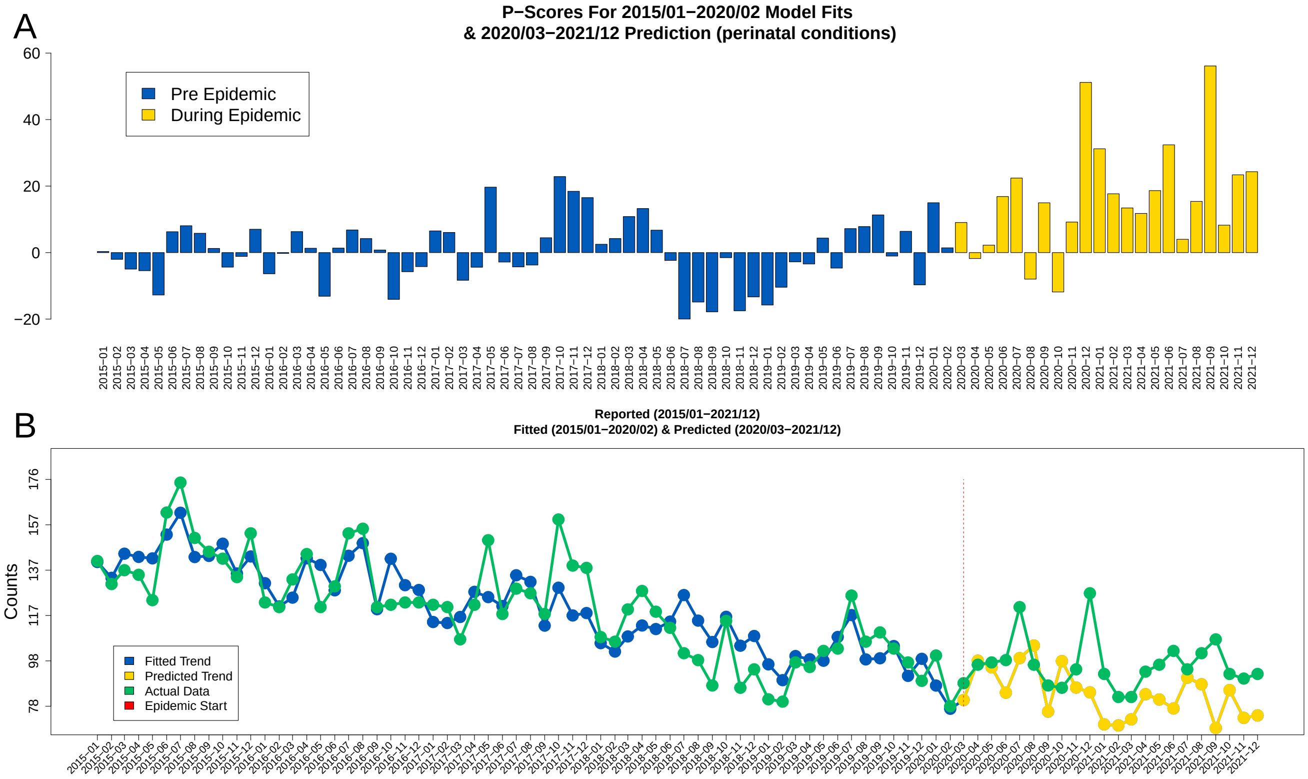

Figure S71. The visual summaries of parametric "perinatal conditions" mortality $\mathscr{P}$-scores based on the Prophet model *without* demographic characteristics are presented on panel A. The yellow bars represent the epidemic period. The corresponding Prophet model fits (blue) and predictions (yellow) based on the 2015-2020 data (panel B) are presented along the reported data (green). Vertical red line indicate the epidemic start period.

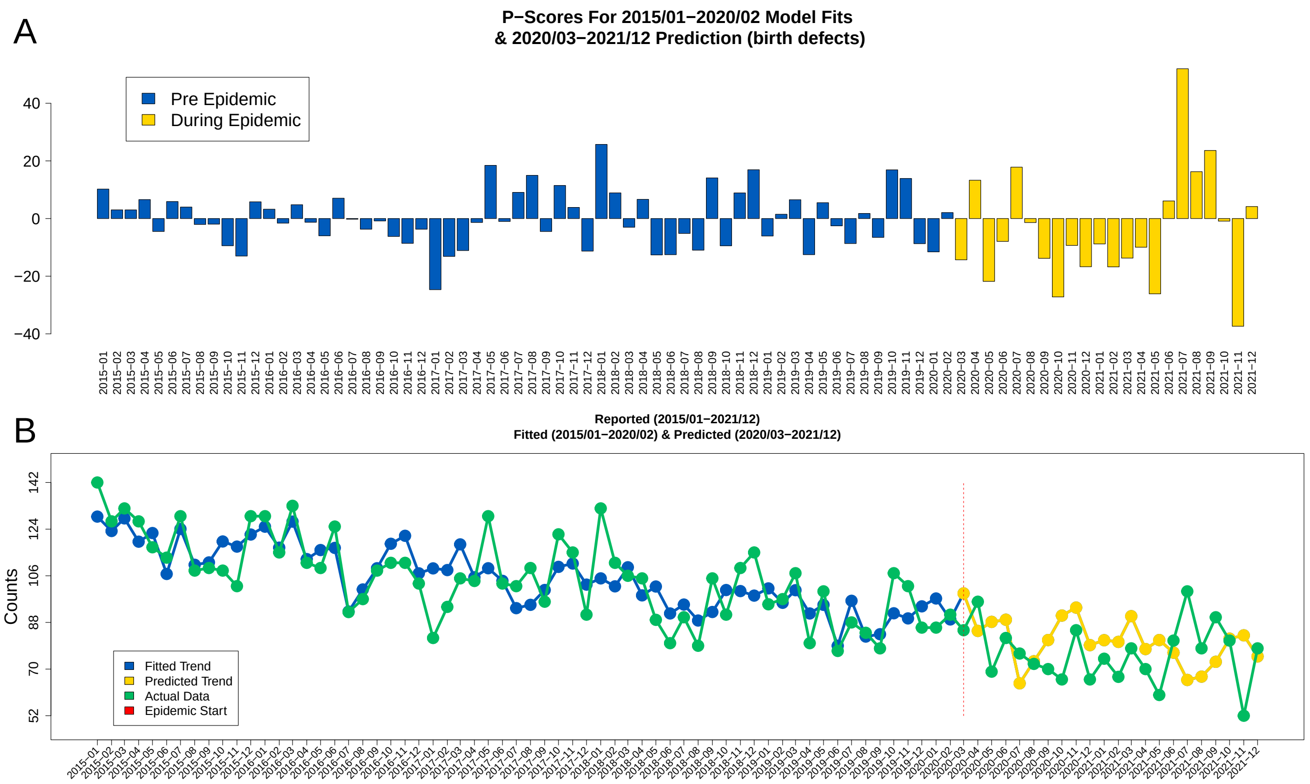

Figure S72. The visual summaries of parametric "birth defects and chromosome anomalies" mortality $\mathscr{P}$-scores based on the Prophet model *without* demographic characteristics are presented on panel A. The yellow bars represent the epidemic period. The corresponding Prophet model fits (blue) and predictions (yellow) based on the 2015-2020 data (panel B) are presented along the reported data (green). Vertical red line indicate the epidemic start period.

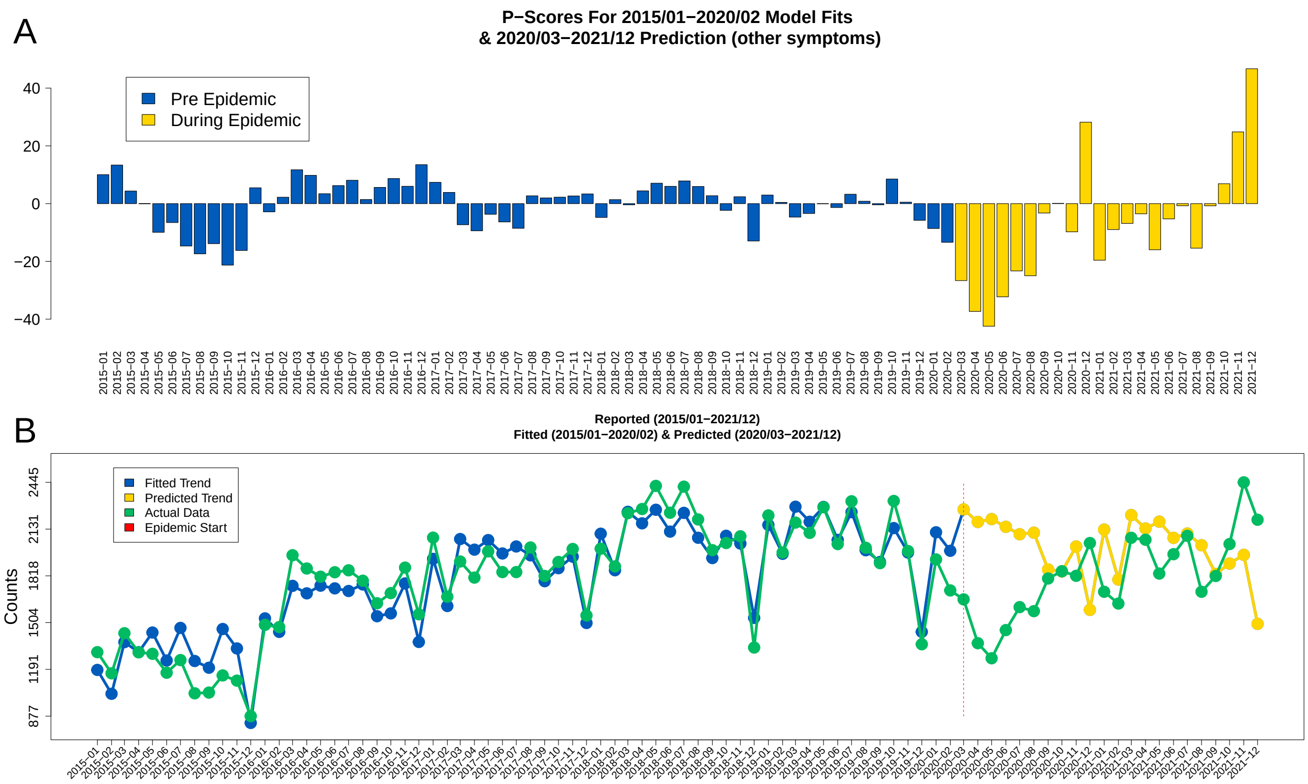

Figure S73. The visual summaries of parametric "other symptoms not classified as any above" mortality $\mathscr{P}$-scores based on the Prophet model *without* demographic characteristics are presented on panel A. The yellow bars represent the epidemic period. The corresponding Prophet model fits (blue) and predictions (yellow) based on the 2015-2020 data (panel B) are presented along the reported data (green). Vertical red line indicate the epidemic start period.

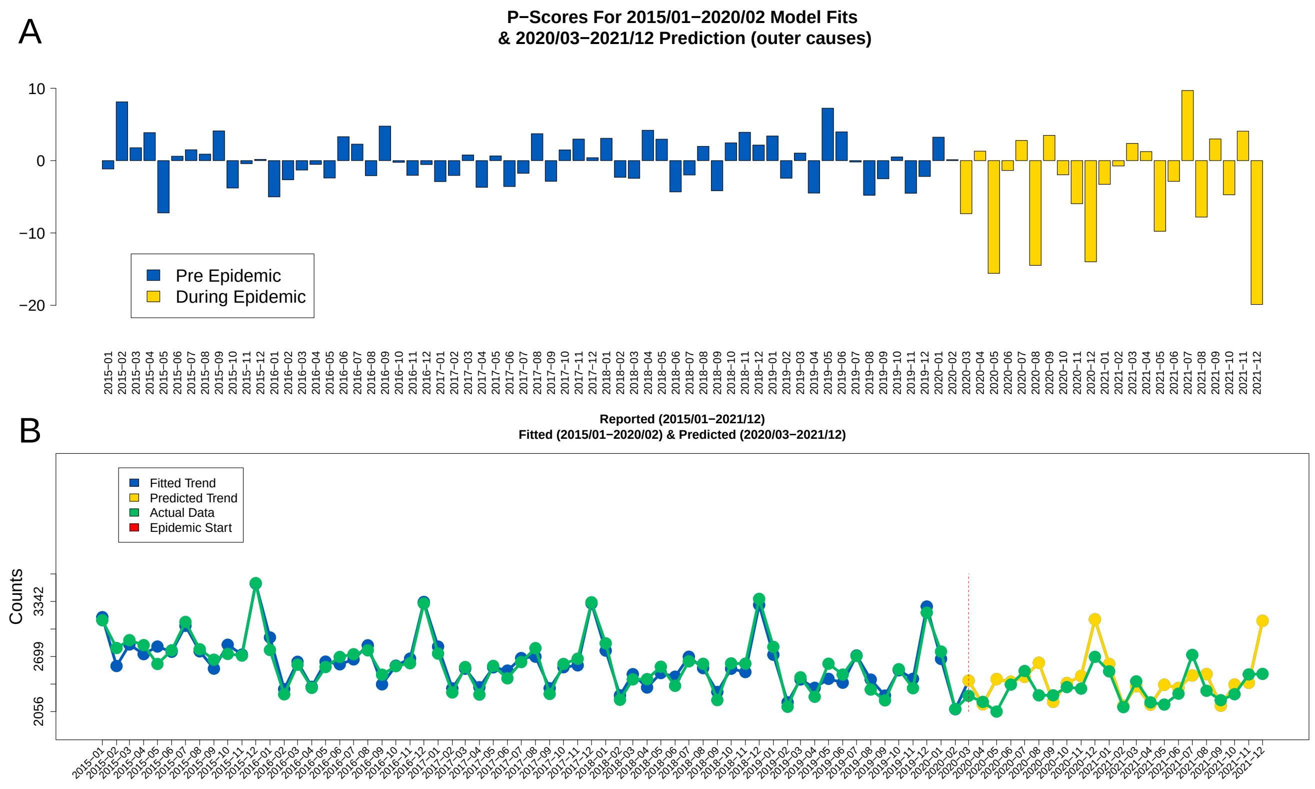

Figure S74. The visual summaries of parametric "outer causes" mortality $\mathscr{P}$-scores based on the Prophet model *without* demographic characteristics are presented on panel A. The yellow bars represent the epidemic period. The corresponding Prophet model fits (blue) and predictions (yellow) based on the 2015-2020 data (panel B) are presented along the reported data (green). Vertical red line indicate the epidemic start period.

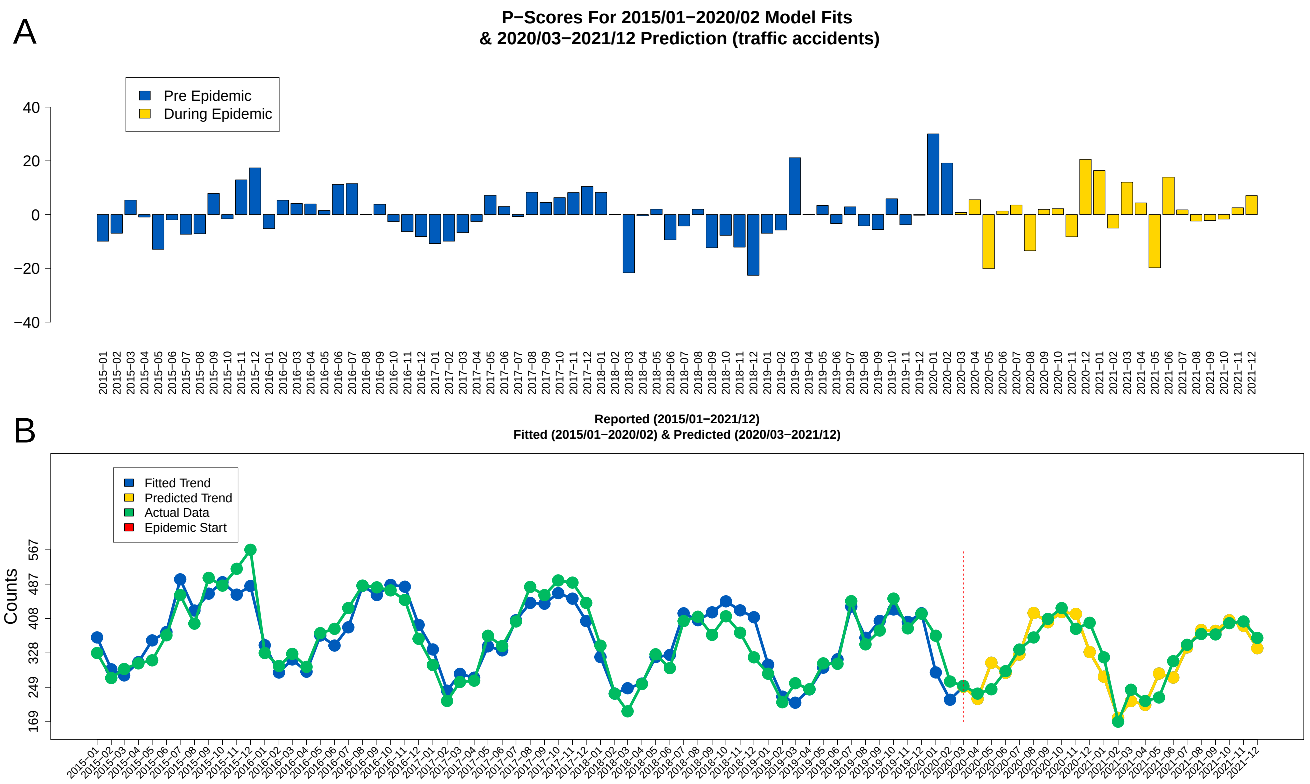

Figure S75. The visual summaries of parametric "traffic accidents" mortality $\mathscr{P}$-scores based on the Prophet model *without* demographic characteristics are presented on panel A. The yellow bars represent the epidemic period. The corresponding Prophet model fits (blue) and predictions (yellow) based on the 2015-2020 data (panel B) are presented along the reported data (green). Vertical red line indicate the epidemic start period.

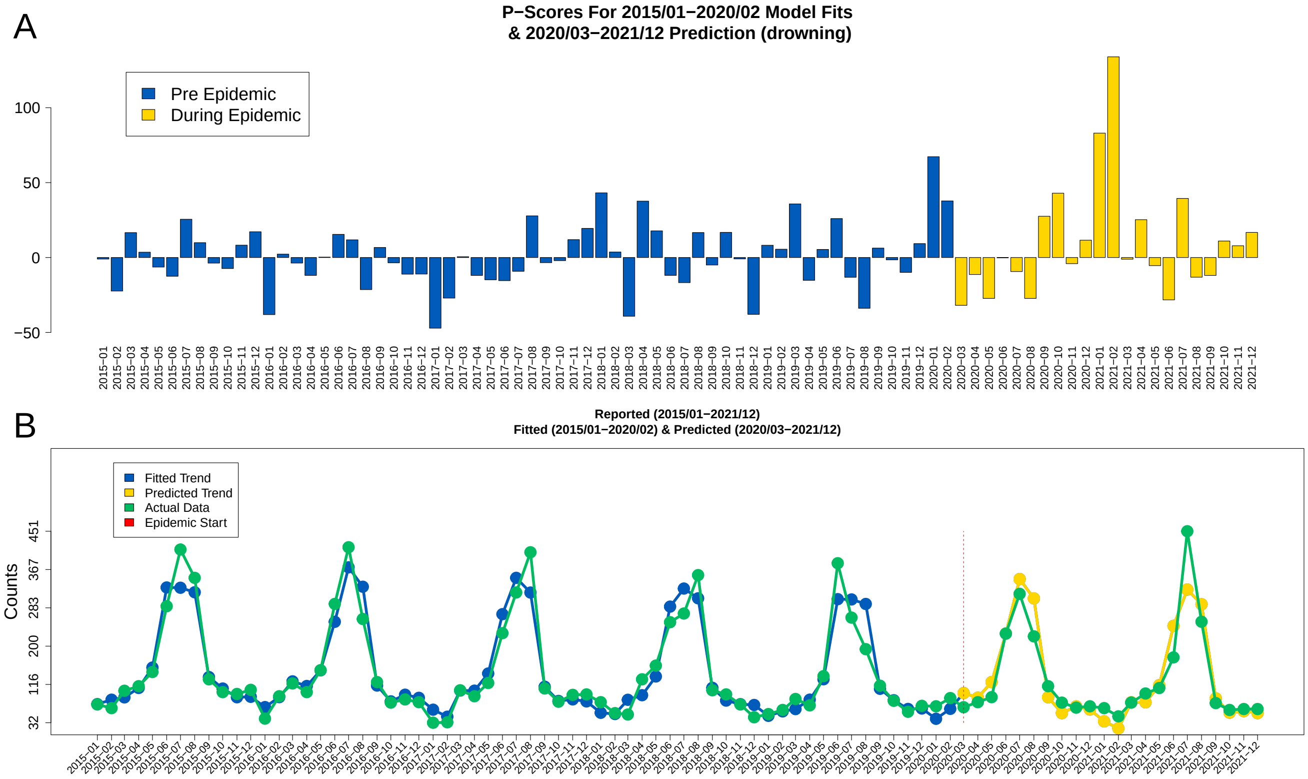

Figure S76. The visual summaries of parametric "drowning" mortality $\mathscr{P}$-scores based on the Prophet model *without* demographic characteristics are presented on panel A. The yellow bars represent the epidemic period. The corresponding Prophet model fits (blue) and predictions (yellow) based on the 2015-2020 data (panel B) are presented along the reported data (green). Vertical red line indicate the epidemic start period.

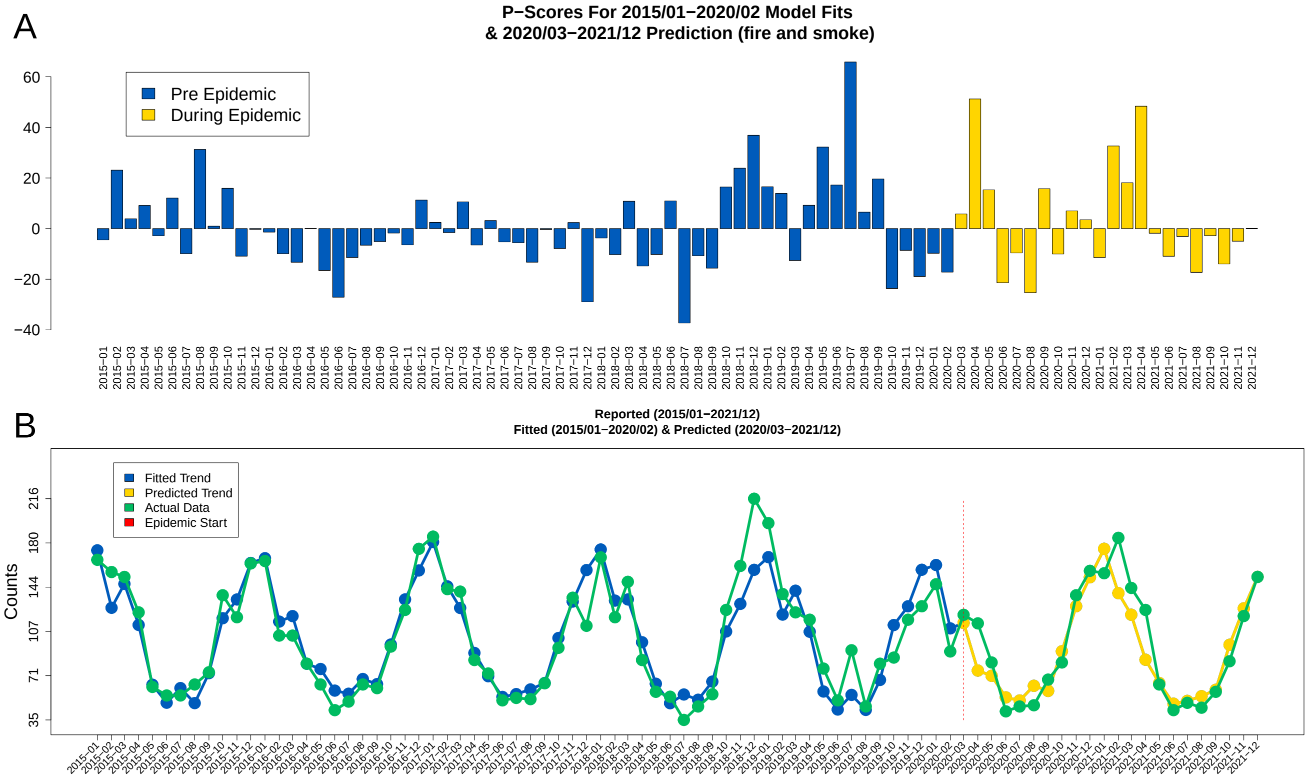

Figure S77. The visual summaries of parametric "fire and smoke" mortality $\mathscr{P}$-scores based on the Prophet model *without* demographic characteristics are presented on panel A. The yellow bars represent the epidemic period. The corresponding Prophet model fits (blue) and predictions (yellow) based on the 2015-2020 data (panel B) are presented along the reported data (green). Vertical red line indicate the epidemic start period.

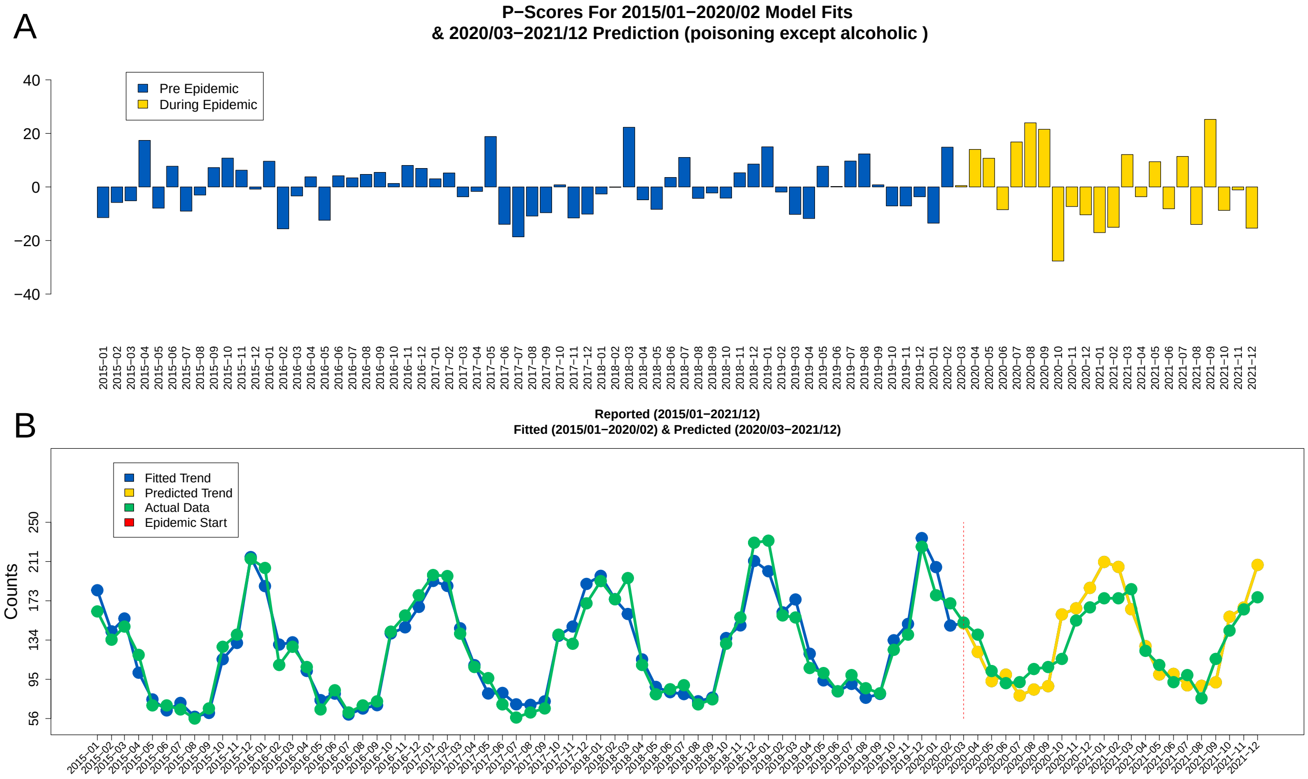

Figure S78. The visual summaries of parametric "poisoning, except alcoholic poisoning" mortality $\mathscr{P}$-scores based on the Prophet model *without* demographic characteristics are presented on panel A. The yellow bars represent the epidemic period. The corresponding Prophet model fits (blue) and predictions (yellow) based on the 2015-2020 data (panel B) are presented along the reported data (green). Vertical red line indicate the epidemic start period.

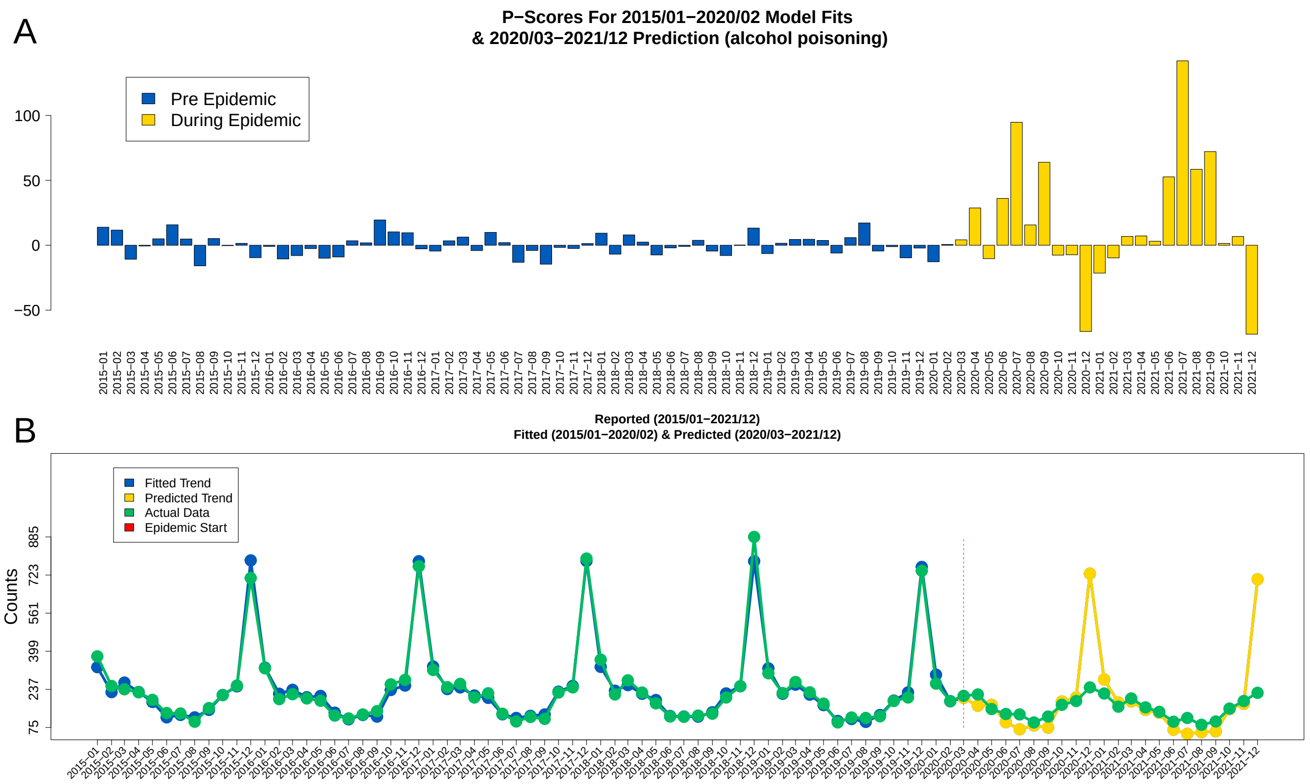

Figure S79. The visual summaries of parametric "alcoholic poisoning" mortality $\mathscr{P}$-scores based on the Prophet model *without* demographic characteristics are presented on panel A. The yellow bars represent the epidemic period. The corresponding Prophet model fits (blue) and predictions (yellow) based on the 2015-2020 data (panel B) are presented along the reported data (green). Vertical red line indicate the epidemic start period.

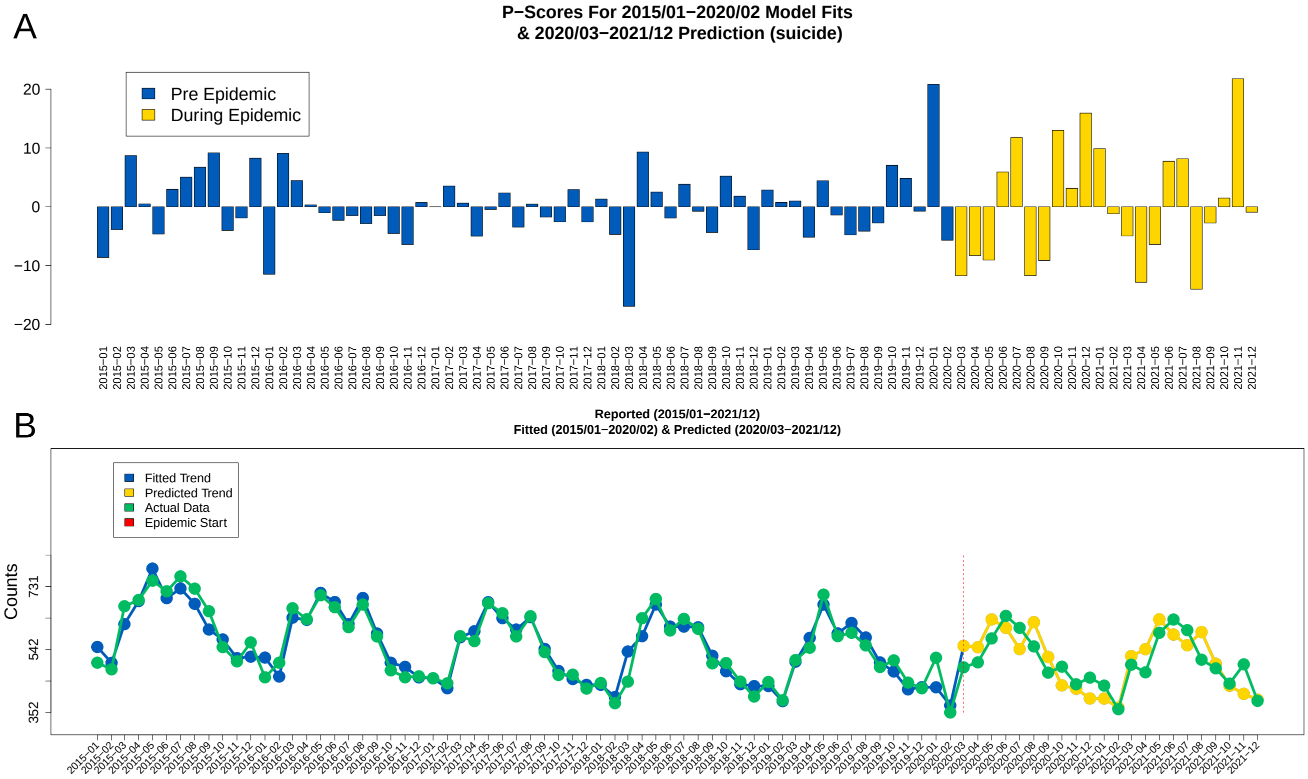

Figure S80. The visual summaries of parametric "suicide" mortality $\mathscr{P}$-scores based on the Prophet model *without* demographic characteristics are presented on panel A. The yellow bars represent the epidemic period. The corresponding Prophet model fits (blue) and predictions (yellow) based on the 2015-2020 data (panel B) are presented along the reported data (green). Vertical red line indicate the epidemic start period.

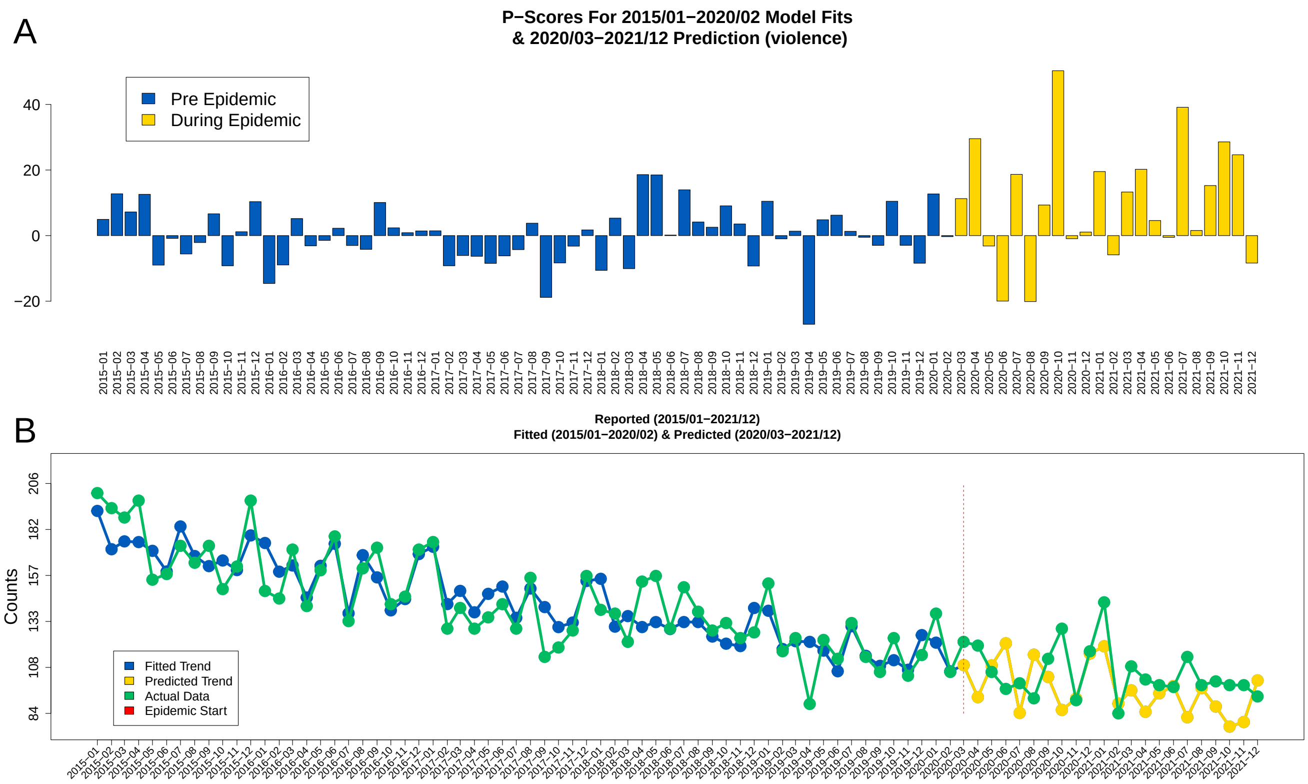

Figure S81. The visual summaries of parametric "violence" mortality $\mathscr{P}$-scores based on the Prophet model *without* demographic characteristics are presented on panel A. The yellow bars represent the epidemic period. The corresponding Prophet model fits (blue) and predictions (yellow) based on the 2015-2020 data (panel B) are presented along the reported data (green). Vertical red line indicate the epidemic start period.
